# Methodological quality of systematic reviews and meta-analyses of rheumatoid arthritis pharmacotherapy: A protocol for a meta-epidemiological study using AMSTAR 2

**DOI:** 10.1371/journal.pone.0359531

**Published:** 2026-10-06

**Authors:** Maoyi Yang, Qifan Cao, Delin Chen, Zhaoxin Yang, Zhipeng Hu

**Affiliations:** 1 Hospital of Chengdu University of Traditional Chinese Medicine, Chengdu, Sichuan, China; 2 Bazhong Hospital of Traditional Chinese Medicine, Bazhong, Sichuan, China; Athens Medical Group, Psychiko Clinic, GREECE

## Abstract

**Background:**

With the exponential growth of systematic reviews and meta-analyses on RA pharmacotherapy, a comprehensive and up-to-date assessment of methodological quality is urgently needed to gauge the credibility of this evidence base.

**Objective:**

This protocol describes a meta-epidemiological study that will systematically appraise the methodological quality of published systematic reviews and meta-analyses of RA pharmacotherapy (csDMARDs, bDMARDs, and tsDMARDs) using the AMSTAR 2 instrument and explore study-level factors associated with methodological quality.

**Methods:**

We will systematically search PubMed, Embase, the Cochrane Library and three Chinese databases-China National Knowledge Infrastructure (CNKI), Wanfang Data and China Science and Technology Journal Database-from inception to June 1, 2026. Two independent reviewers will screen studies, extract data and assess methodological quality using the AMSTAR 2 instrument; an overall rating (high, moderate, low, critically low) will be derived for each review. Binary logistic regression comparing “Adequate” (High/Moderate) versus “Inadequate” (Low/Critically Low) reviews will be used for the primary analysis, with ordinal logistic regression fitted as a pre-specified exploratory analysis; pre-specified sensitivity and robustness analyses (including penalized and Bayesian estimation for sparse categories) will test the stability of findings.

**Results:**

The results will provide a descriptive overview of the methodological quality landscape, highlighting common strengths and critical weaknesses across published RA meta-analyses. The regression analysis will quantify the relationship between specific study characteristics and methodological quality.

**Conclusion:**

This study will provide a comprehensive, cross-drug-class AMSTAR 2-based methodological assessment of the RA pharmacotherapy evidence-synthesis field, complementing prior appraisals limited to specific complementary or single-agent interventions. The findings will offer clinicians and guideline developers a graded framework for judging evidence credibility and will provide empirical, actionable guidance for researchers to enhance the design, conduct, and reporting of future systematic reviews in RA.

## 1. Introduction

Rheumatoid arthritis (RA) is a chronic systemic autoimmune disease characterized primarily by symmetric erosive arthritis and represents one of the most common chronic inflammatory arthropathies responsible for joint deformity [1]. A meta-analysis that included 67 studies showed that the pooled prevalence of RA was about 0.46% (95% CI 0.37–0.57%) for the period 1986–2014 [2]. According to the results of 2017 GBD (Global Burden of Disease) study, the global age-standardized prevalence of RA increased by 7.4% (95% CI 5.3–9.4%) between 1990 and 2017 [3]. Significant economic costs are incurred annually due to RA. In China, the average annual total cost per patient is approximately 41,971 RMB [4].

Over the past three decades, the treatment of RA has advanced rapidly. Therapeutic agents, including conventional synthetic disease-modifying antirheumatic drugs (csDMARDs), biological DMARDs (bDMARDs), and targeted synthetic DMARDs (tsDMARDs), have delivered more individualized treatment options for patients with RA [5]. Meanwhile, the number of randomized controlled trials (RCTs) evaluating the efficacy and safety of distinct drug classes across different treatment settings and patient subgroups has risen exponentially. As the highest level of evidence in the evidence-based medicine hierarchy, systematic reviews and meta-analyses derived from these RCTs serve as the core evidence base for the development of global RA clinical practice guidelines, insurance reimbursement, and clinical prescribing decisions, directly shaping the care outcomes of tens of millions of RA patients worldwide.

Systematic reviews must provide a precise presentation of current knowledge. This precision is critical because such reviews directly inform clinical and health policy decisions that affect patient outcomes. They also serve as a key evidence base for regulatory approval and insurance coverage decisions. The clinical value and credibility of systematic review and meta-analysis conclusions depend entirely on the rigor of study design and the standardization of methodological performance. Accordingly, methodological appraisal tools (AMSTAR 2) have been developed to ensure transparent, standardized, and rigorous conduct of systematic reviews [6].

In recent years, alongside the rapid accumulation of evidence in RA treatment, the number of published meta-analyses on RA pharmacotherapy has continued to increase. To our knowledge, while previous AMSTAR 2-based appraisals have focused on specific complementary/alternative therapies [7], single-agent JAK inhibitors [8], or intra-articular therapies [9], no prior study has provided a cross-drug-class methodological assessment covering the full spectrum of csDMARDs, bDMARDs, and tsDMARDs with regression-based factor analysis. Our study therefore addresses a distinct gap by offering a broader, more inclusive evidence-quality map for RA pharmacotherapy. This gap prevents clinicians, guideline panels, and health policymakers from comprehensively and reliably evaluating the overall quality and credibility of the current evidence base for RA pharmacotherapy, potentially leading to misguided clinical decision-making. For researchers, the absence of comprehensive methodological appraisal hinders the identification of methodological flaws and biases in existing evidence, resulting in research waste and the potential perpetuation of misguided research priorities.

In this study, we aim to comprehensively and rigorously assess the methodological quality of meta-analyses on RA pharmacotherapy using the AMSTAR 2 tool and point out the shortcomings in the current field. On this basis, regression analysis will be performed to further explore factors associated with methodological quality. The findings of this study will provide a graded reference framework for evidence credibility to support the development of RA clinical guidelines and the optimization of clinical decision-making. Furthermore, this work will identify clear improvement directions for the standardized design and conduct of future systematic reviews and meta-analyses in RA research, ultimately promoting the high-quality development of evidence-based RA care systems and improving long-term clinical outcomes for patients with RA globally.

To bridge this critical evidence gap, we will conduct a comprehensive methodological appraisal of published SRs/MAs on RA pharmacotherapy using the AMSTAR 2 tool. Crucially, this is a meta-epidemiological investigation: the unit of analysis is the systematic review itself, and the outcome of interest is methodological quality, rather than the synthesis of pooled clinical effect estimates from primary trials [10]. Specifically, we address three core questions: (i) the overall quality profile of existing evidence, (ii) study-level determinants (e.g., registration, protocol, funding) associated with higher rigor, and (iii) temporal trends following the 2017 dissemination of AMSTAR 2. By shifting the focus from what the treatment does to how trustworthy the aggregated evidence is, our findings will provide a graded credibility framework for guideline developers and payers, while offering actionable methodological benchmarks to reduce research waste and enhance the reproducibility of future evidence syntheses in RA.

## 2. Methods

### 2.1. Design and registration

This protocol was reported according to Preferred Reporting Items for Systematic Review and Meta-Analysis Protocols [11] (S1 File). The meta-epidemiological research will be reported according to corresponding reporting guideline [10]. This protocol has been registered in PROSPERO (CRD420261359521). Available at https://www.crd.york.ac.uk/PROSPERO/view/CRD420261359521. The registration record was updated during peer review to reflect the revised methods presented in this version of the protocol; all amendments are documented in the registration history.

### 2.2. Database searching

Relevant literature will be searched in three English databases including PubMed, Embase and the Cochrane Library and three Chinese databases including China National Knowledge Infrastructure (CNKI), Wanfang Data Knowledge Service Platform and China Science and Technology Journal Database from its inception to June 1st, 2026. We will use a combined strategy of MeSH terms and free text words and literature related to systematic reviews will be searched using specifically developed and validated search filters without restrictions on publication date or language [12]. To further identify eligible studies, we will also search the reference lists of included studies, conference articles, review articles on this topic, and clinical guidelines. To ensure the inclusion of the most up-to-date evidence, an updated search will be performed towards the end of the review process to avoid omitting newly published studies that emerged after the initial search. The implementation and reporting of the literature search will be conducted in accordance with the Preferred Reporting Items for Systematic Reviews and Meta-Analyses for Abstracts and Search Strategies (PRISMA‑S) checklist [13]. The full search strategy for PubMed, including exact MeSH terms, free-text terms, Boolean operators, filters, and search dates, is provided in S2 File, in accordance with PRISMA-S. Literature searching will be designed and conducted by Maoyi Yang and Qifan Cao.

### 2.3. Study selection

Records will be imported into EndNote for de-duplication and into Rayyan for screening. The Rayyan blind-on mode will be enabled to ensure that reviewers are masked to each other’s decisions throughout the screening process; two reviewers (Maoyi Yang, Qifan Cao) will independently screen titles/abstracts and full texts, with disagreements resolved by a third reviewer (Zhipeng Hu). A comprehensive inventory of all included studies, along with details of studies excluded after full-text appraisal and the corresponding exclusion reason, will be reported transparently in the final article and detailed data will be available. The complete literature selection workflow will be visualized in a standard PRISMA flow diagram.

### 2.4. Inclusion and exclusion criteria

Participants: Eligible reviews are those whose included population is patients with rheumatoid arthritis as defined by the review authors, irrespective of the specific classification criteria (e.g., 1987 ACR or 2010 ACR/EULAR) used by the primary studies. We frame this criterion at the review level: a review is eligible if its authors state that they included patients with RA, regardless of whether the underlying primary studies used the 1987 or 2010 criteria. Reviews of RA-related complications (e.g., hepatic impairment, interstitial lung disease) remain eligible if the primary intervention is pharmacotherapy.

Intervention: reviews whose primary aim is to evaluate RA pharmacotherapy (all csDMARDs, bDMARDs, and tsDMARDs) will be included. Reviews evaluating exclusively complementary/alternative therapies, lifestyle interventions, physiotherapy, or surgical procedures will be excluded. For reviews that assess both pharmacological and non-pharmacological interventions, inclusion will be considered only if pharmacotherapy constitutes the primary comparison and the majority of included primary studies evaluate drug interventions; such decisions will be recorded and tested in a sensitivity analysis (Section 2.7, analysis 5). To ensure conceptual consistency with our title and objective, all included reviews must have pharmacotherapy (csDMARDs, bDMARDs, or tsDMARDs) as the central intervention of interest. For scope clarity, biosimilars of biological DMARDs will be classified under the corresponding bDMARD category and remain eligible; reviews whose primary aim is exclusively the evaluation of glucocorticoids, non-steroidal anti-inflammatory drugs, or intra-articular pharmacological agents in RA will be excluded, as these lie outside the csDMARDs/bDMARDs/tsDMARDs framework central to this study.

Comparators: This study will not impose any restrictions on the type of control group.

Outcomes: Reviews reporting any health-related outcome of RA patients are eligible. To ensure consistency across reviewers, we have predefined the following expected primary outcomes to be extracted: disease activity (DAS28, ACR20/50/70 response), physical function (HAQ), pain (VAS or other validated scales), radiographic progression (Sharp/van der Heijde-modified Sharp scores), and serious adverse events. These correspond to the OMERACT RA core outcome set. Reviews reporting only health-economic outcomes without clinical effectiveness data will be excluded unless the clinical-effectiveness component is embedded within the same review.

Study design: pairwise meta-analyses of RCTs or non-randomized intervention studies that have undergone quantitative synthesis will be included. Specifically, meta-analyses synthesizing observational intervention studies (e.g., cohort studies, case-control studies) are eligible, provided they have conducted a quantitative synthesis. This decision will be recorded and tested in a sensitivity analysis by comparing reviews based on RCTs only vs. those including observational studies (Section 2.7, analysis 6). Systematic reviews without quantitative synthesis, scoping reviews, abstract-only papers, diagnostic-test-accuracy meta-analyses, individual-patient-data meta-analyses, network meta-analyses, and realist reviews will be excluded. While AMSTAR 2 can technically be applied to systematic reviews without meta-analysis (with certain items rated “not applicable”), we have elected as a deliberate scope decision to restrict inclusion to pairwise meta-analyses of RCTs or non-randomized intervention studies that have undergone quantitative synthesis. This restriction ensures comparability of AMSTAR 2 item ratings across reviews-particularly for Item 11 (appropriate meta-analytic methods) and Item 13 (accounting for risk of bias in interpretation)-and avoids heterogeneous “not applicable” responses that would complicate our regression analysis. We explicitly acknowledge that this is a scope decision, not a claim that AMSTAR 2 is inapplicable to non-quantitative reviews [6].

### 2.5. Data extraction

Two investigators (Zhaoxin Yang and Delin Chen) will independently extract data using a predefined form: number of authors, publication year, country and economic-development level of the corresponding author, number of primary studies and participants, study design(s), classification criteria, Cochrane review or not, pre-registration, intervention/comparator details, outcomes, funding source reporting, and protocol-publication status, reporting completeness according to the PRISMA 2020 checklist (adherence proportion), and use of GRADE for evidence certainty grading (yes/no/unclear). Extracted outcomes will be categorized according to the OMERACT RA core outcome set to ensure harmonized classification across included reviews. Reporting completeness will be quantified as the proportion of the 27 PRISMA 2020 checklist items satisfied by each review, with each item scored as Yes (1), Partial Yes (0.5), or No (0), and the adherence proportion calculated as the summed score divided by 27. Use of GRADE will be defined as explicit GRADE-based rating of evidence certainty, operationalized as the presence of GRADE evidence profiles or summary-of-findings tables with certainty ratings, or an explicit statement of GRADE-based grading; reviews with none of these will be classified as “unclear” and treated conservatively as “No” in the regression analyses, with the proportion of “unclear” classifications reported. Inter-rater agreement for these assessments will be quantified using the intraclass correlation coefficient (PRISMA adherence proportion) and Cohen’s kappa (GRADE use). For unreported or unclear data, corresponding authors will be contacted by email (up to two reminders over four weeks); if data remain unavailable, the relevant item will be rated conservatively (i.e., scored as “No” for AMSTAR 2 items or as “unclear” for extracted characteristics) and the absence will be explicitly recorded. A sensitivity analysis will be conducted excluding reviews with >20% missing data to assess the robustness of the primary findings to missing data assumptions (Section 2.7, analysis 7). All missing data will be reported transparently in the final article with clear annotations [14]. Disagreements will be resolved by discussion with a third reviewer (Zhipeng Hu).

### 2.6. Methodological quality assessment using AMSTAR 2

The methodological quality of included reviews will be assessed using AMSTAR 2, a critical-appraisal instrument for systematic reviews of healthcare interventions (16 items, 7 critical); AMSTAR 2 is not a risk-of-bias tool in the RoB 2/ROBINS-I sense. The unit of analysis is the individual systematic review/meta-analysis; for duplicate or updated versions of the same review, the most recent and most complete version will be retained; for reviews containing multiple meta-analyses, AMSTAR 2 will be applied at the review level. To derive a single valid rating for a review containing multiple meta-analyses, review-level items (e.g., Items 2, 4, 7, 9) will be rated for the review as a whole, whereas meta-analysis-level items (e.g., Items 11, 13, 14, 15) will be rated conservatively: when the included meta-analyses differ, the item will be rated according to the meta-analysis of the lowest quality, to avoid operator discretion and to ensure comparability across reviews. The number of meta-analyses within each review and any within-review inconsistency will be recorded in the extraction form and will be reported in the final publication of the planned meta-epidemiological study. Each item will be judged as “Yes”, “Partial Yes”, or “No”; an overall rating of High, Moderate, Low, or Critically Low will be derived using the AMSTAR 2 algorithm.

Two reviewers (Zhaoxin Yang and Delin Chen) will independently apply AMSTAR 2. Inter-rater reliability will be quantified using Cohen’s kappa for each item and a weighted kappa (or intraclass correlation coefficient, ICC) for the overall four-level rating, interpreted as: poor <0.40, moderate 0.40–0.60, good 0.61–0.80, very good >0.80. Disagreements will be resolved by a third reviewer (Zhipeng Hu). In accordance with the AMSTAR 2 algorithm [6], a “Partial Yes” on a critical item does not constitute a critical weakness and therefore does not by itself trigger downgrading; only “No” responses are counted as weaknesses. The overall rating is derived as follows: High-no or only one non-critical weakness; Moderate-more than one non-critical weakness; Low-one critical weakness with or without non-critical weaknesses; Critically Low-more than one critical weakness with or without non-critical weaknesses.

The seven critical AMSTAR 2 items are: Item 2 (registered protocol), Item 4 (adequacy of search), Item 7 (list of excluded studies with justification), Item 9 (risk-of-bias assessment of individual studies), Item 11 (appropriate meta-analytic methods), Item 13 (accounting for risk of bias in interpretation), and Item 15 (assessment of publication bias). We will report detailed justification for each item rating transparently in the final review publication, following the structured format recommended by Shea et al.

### 2.7. Data analysis

Continuous variables will be summarized as mean ± standard deviation or median with interquartile range depending on distribution of data. Dichotomous variables will be summarized using frequencies and percentages. The distribution of item ratings (“Yes/Partial Yes/No”) will be displayed using stacked bar charts (replacing the radar chart). Item-level adherence before vs after the 2017 publication of AMSTAR 2 will be compared using odds ratios (or risk differences) with 95% confidence intervals (CIs).

According to previously published results, we predefined the following exposure variables: the adherence to the PRISMA 2020 statement (continuous, measured as the adherence proportion), prospective registration or study protocols (PROSPERO or other registration platforms; binary), and use of GRADE for evidence certainty grading (binary variable). The model also includes the following covariates to close backdoor paths: Cochrane systematic review source (binary variable), type of included studies (only RCTs vs. including non-RCTs), funding source (industry/non-profit/none/not reported), publication year (continuous variable), and geographical region of the corresponding author, categorized as developed vs. developing countries using the World Bank income classification (high-income vs. non-high-income economies) based on the country’s classification in the year of publication [15–17]. The model also includes intervention class (csDMARDs only/ bDMARDs only/ tsDMARDs only/ mixed, where “mixed” denotes reviews evaluating more than one DMARD class) as a covariate, added to account for differing regulatory and reporting standards across drug classes, which may influence the methodological rigor of reviews. To avoid incorporation bias, pre-registration will not be entered as a predictor of the composite AMSTAR 2 rating in the primary model. Instead, in a pre-specified secondary analysis, the overall rating will be recalculated after excluding Item 2 (registered protocol) from the algorithm; pre-registration will then be reintroduced as a predictor in this secondary model. This approach assesses the association between pre-registration and the non-protocol components of methodological quality without circularity. The exclusion of Item 2 from the primary model is explicitly acknowledged as a limitation, and results from both models will be reported transparently. For ease of interpretation, we will also report the odds ratio for each variable per one standard-deviation increase. Multicollinearity between covariates will be assessed by calculating the variance inflation factor (VIF), and VIF values for all variables will be reported. A VIF ≥ 5 will be defined as the presence of significant multicollinearity, and ridge regression will be performed as an alternative model if multicollinearity is detected.

Our primary analysis will model methodological quality as a binary outcome- “Adequate” (overall rating High or Moderate) versus “Inadequate” (Low or Critically Low)-using binary logistic regression. This dichotomy was pre-specified because published methodological audits in analogous fields indicate that 70–90% of included reviews are expected to fall into the Low or Critically Low categories; the binary outcome maximizes model stability under this expected skewed distribution and provides the clinically most interpretable split. The primary model will contain three theoretically important covariates-Cochrane systematic review source, intervention class, and PRISMA adherence-chosen a priori; the remaining covariates will be examined in secondary models rather than entered in a stepwise manner, to keep the primary model parsimonious (Section 2.8). Adjusted odds ratios (OR) with 95% CIs will be reported for each predictor, together with model performance assessed by the C statistic (area under the ROC curve) for discrimination and by calibration. We will first conduct univariate analyses of each independent variable against the outcome, and then the multiple regression, comparing unadjusted and adjusted results. If the event count in the smaller outcome group is insufficient (events-per-parameter ratio < 10) or complete/quasi-complete separation occurs, Firth’s bias-reduced penalized logistic regression will be used, and penalized profile-likelihood confidence intervals will be reported [18].

As a pre-specified exploratory analysis, a four-level ordinal logistic regression (OLR) will be fitted to the original AMSTAR 2 rating (High/Moderate/Low/Critically Low), but only if each collapsed category contains at least 10 reviews (see Section 2.8); otherwise, the ordinal model will not be fitted, to avoid overfitting. When fitted, the proportional odds assumption will be verified using the Brant test; if the assumption is violated (*P* < 0.05), a generalized ordered logit model will be used. Overall model significance will be assessed by the likelihood ratio test, and McFadden’s pseudo R^2^ will be calculated to quantify explanatory power. As a robustness check for sparse ordinal categories, a Bayesian ordinal regression with weakly informative priors (brms, cumulative family) will additionally be fitted, providing shrinkage against overfitting [19].

Reviews using Bayesian or hierarchical pairwise meta-analytic models remain eligible, and AMSTAR 2 items will be applied as applicable: Item 11 (appropriate meta-analytic methods) will be evaluated based on whether the Bayesian/hierarchical model was appropriately specified and justified, and Item 13 (accounting for risk of bias in interpretation) using the same criteria as for frequentist meta-analyses. These reviews will not be excluded solely on the basis of their statistical approach.

To test the robustness of the results, seven pre-specified sensitivity analyses will be conducted: (1) the four-level outcome will be collapsed alternatively into three categories (High/Moderate vs. Low vs. Critically Low) and re-analyzed where cell counts permit; (2) publication year will be dichotomized (before vs. after the 2017 publication of AMSTAR 2); (3) the analysis will be restricted to reviews evaluating a single DMARD class (csDMARDs only, bDMARDs only, or tsDMARDs only), excluding reviews evaluating multiple DMARD classes (“mixed”), to test whether the observed associations are driven by reviews spanning multiple DMARD classes; (4) reviews containing multiple meta-analyses will be excluded to test the robustness of the conservative rating rule; (5) reviews including both pharmacological and non-pharmacological interventions will be excluded to test the robustness of the boundary inclusion decisions recorded in Section 2.4; (6) reviews synthesizing only RCTs will be compared with reviews also including non-randomized intervention studies; and (7) reviews with more than 20% missing data will be excluded to test the robustness of the findings to the conservative missing-data handling described in Section 2.5. All sensitivity analyses will be subject to the same estimation and sample-size safeguards pre-specified above and in Section 2.8.

All statistical analyses will be conducted using R (Version 4.3.1; R Foundation for Statistical Computing, Vienna, Austria) with the RStudio integrated development environment (Version 2023.06.2; RStudio, PBC, Boston, Massachusetts, USA). The statistical significance level will be set at α = 0.05 (two-tailed).

### 2.8. Model feasibility and sample size

Based on published methodological audits in analogous fields [15,16], we anticipate that 70–90% of included reviews will fall into the Low or Critically Low categories. The evidence base is of sufficient scale for the target sample size: a single, conservative PubMed search (title/abstract) combining “rheumatoid arthritis” with “meta-analysis” and representative drug terms retrieves more than 2400 records, and our six-database, language-unrestricted search draws from a substantially larger candidate pool. We therefore anticipate more than 300 reviews will be included, and we will estimate the required minimum sample size formally using the pmsampsize package [20], with the Peduzzi rule (N = 10k/p, where k is the number of predictors and p the proportion in the smaller outcome group) as a cross-check [21].

The primary model will contain three theoretically important covariates-Cochrane systematic review source (binary), intervention class (categorical, four levels), and PRISMA adherence (continuous). Counting model parameters rather than variables, the four-level intervention class contributes three indicator variables, so the model contains five parameters in total; with N ≥ 300 and a 10–15% expected proportion of Adequate reviews (30–45 events in the smaller outcome group), the events-per-parameter ratio is 30–45 ÷ 5 = 6–9, below the conventional threshold of 10 [21]. The primary analysis will therefore be estimated using Firth’s bias-reduced penalized logistic regression with penalized profile-likelihood confidence intervals, as pre-specified in Section 2.7; unpenalized maximum-likelihood estimates will additionally be reported if the realized events-per-parameter ratio reaches 10. The remaining six pre-specified exposures and covariates will be examined only in secondary models, and no stepwise variable selection will be performed, to avoid overfitting and multiple-testing inflation. Prospective registration will not be entered as a predictor in the primary model (see Section 2.7) and will be retained only in the secondary model after exclusion of Item 2.

To avoid dependence on any single realized sample size, we pre-specify the following analysis ladder. If 200–299 reviews are included, the three-predictor primary model is retained, with the events-per-parameter ratio recomputed for the observed distribution (Firth estimation applying as above). If 100–199 reviews are included, the primary model is reduced to the two most theoretically important covariates (Cochrane source and PRISMA adherence), and the remaining covariates are analyzed univariately. If fewer than 100 reviews are included, regression is abandoned in favor of the descriptive methodological-quality appraisal. In addition, if any of the four AMSTAR 2 rating categories contains fewer than 10 reviews, the ordinal outcome will be collapsed (e.g., High/Moderate vs. Low vs. Critically Low, or Adequate vs. Inadequate) and modeled accordingly; the four-level ordinal model will be fitted only when each collapsed category contains at least 10 reviews. The realized sample size and the analysis tier adopted will be reported with the PRISMA flow diagram, and a priori power calculations based on the anticipated event rate in the least frequent rating category will be reported to demonstrate whether the final model is adequately powered, with the number of predictors adjusted to the available sample. If the realized proportion of Adequate reviews is very low (e.g., < 10%), the regression results will be interpreted as exploratory, as acknowledged in the Limitations section.

### 2.9. Ethics statement

This study is a protocol for meta-epidemiological research that will synthesize data from previously published studies. No new human participants will be recruited, and no original human data or biological samples will be collected. The study will use publicly available aggregated, anonymized data. Therefore, ethical approval is not required, and informed consent from participants is not applicable.

## 3. Discussion

Currently, the number of meta-analyses of RA treatment has increased sharply, and these meta-analyses form the core basis for guideline development and clinical decision-making. However, there is a lack of comprehensive, rigorous, and in-depth evaluation of the methodological quality of this body of evidence, which limits the overall understanding of the methodological landscape of the evidence. To date, no study has explored potential factors influencing methodological quality. Understanding these associated factors is crucial for systematically improving the quality of future research.

Based on these limitations, this study aims to: 1. conduct a comprehensive, large-scale, standardized assessment of the methodological quality of systematic reviews and meta-analyses related to drug treatments for RA using the AMSTAR 2 tool, presenting an overall quality map of evidence production in this field and identifying common methodological flaws; and to explore the associations between pre-specified factors and the methodological quality, thereby pointing out specific directions for improving the design and implementation of higher quality systematic reviews. This meta-epidemiological study holds significant importance for clarifying the current state of evidence, guiding future research practices, and optimizing clinical decision-making.

### 3.1. Strengths of this study

This study provides a comprehensive, cross-drug-class methodological assessment focusing on DMARD pharmacotherapy with regression-based factor analysis, complementing prior appraisals limited to complementary/alternative or single-drug interventions [7,9,22]. This analysis can provide strong data support for further improvement.

### 3.2. Limitations of this study

Although this study was designed rigorously, there are some potential limitations. First, the results of regression analysis represent associations rather than causation, and therefore conclusions should be interpreted with caution. Second, according to the original text of the AMSTAR 2 tool, this tool is not suitable for network meta-analyses and individual participant data meta-analysis-the former is an important component increasingly influencing the evidence for RA drug treatments, while the latter is a significant source of high-quality meta-analyses. Excluding NMAs and IPD-MAs may limit the comprehensiveness of our evidence quality map, particularly for RA pharmacotherapy where NMAs are increasingly influential in informing comparative effectiveness among multiple targeted therapies (e.g., head-to-head comparisons of JAK inhibitors vs. biologics). While we acknowledge that AMSTAR 2 is not designed for these complex synthesis designs, this omission means our overview does not capture the methodological rigor of a substantial and growing segment of the evidence base. Future methodological investigations should apply NMA-specific appraisal instruments (e.g., the ISPOR checklist or CINeMA) to address this gap and provide a more complete picture of the evidence landscape. Third, because Item 2 (protocol registration) is itself a component of the AMSTAR 2 overall rating, pre-registration is excluded from the primary model to avoid incorporation bias; the association between registration and methodological quality can therefore only be assessed in the pre-specified secondary analysis, in which the overall rating is recalculated after excluding Item 2. Fourth, if the realized proportion of “Adequate” (High/Moderate) reviews is very low (e.g., < 10%), the number of events will be insufficient to support stable regression inference, and the regression results will be interpreted as exploratory; in that case, the descriptive methodological-quality appraisal will remain the principal contribution of the study.

## 4. Conclusion

This research protocol outlines a comprehensive, in-depth, and methodologically rigorous meta-epidemiological study aimed at revealing the status of methodological quality in core treatment evidence for RA and its influencing factors. It is expected to fill critical knowledge gaps in this field, with results that can serve as a reference for clinical practice and decision-making, as well as provide guidance for future systematic review and meta-analysis researchers. We look forward to the implementation of this study to promote the advancement of evidence-based practice in RA to higher quality and standards.

## Supporting information

S1 File PRISMA-P 2015 Checklist. (DOCX)

S2 File Search strategies for PubMed. (DOCX)
