# Supplementary material for: Methodological quality of systematic reviews and meta-analyses of rheumatoid arthritis pharmacotherapy: A protocol for a meta-epidemiological study using AMSTAR 2
Source: PLoS One. 2026 Oct 6;21(10):e0359531. doi: 10.1371/journal.pone.0359531 (PMC13641359; doi:10.1371/journal.pone.0359531)
Supplement: Supplementary file 1 [file pone.0359531.s003.pdf]

Title:

Methodological Assessment of Systematic Review and Meta-analysis in Rheumatoid Arthritis: A Protocol for Meta-epidemiological Research

Recommendation: Minor Revision

Overall Evaluation

This manuscript presents a protocol for a meta-epidemiological study aiming to assess the methodological quality of systematic reviews and meta-analyses on rheumatoid arthritis (RA) pharmacotherapy using the AMSTAR 2 tool. The topic is timely and important given the rapid expansion of evidence synthesis studies in RA treatment. The protocol demonstrates several methodological strengths, including protocol registration, predefined covariates, and planned sensitivity analyses. However, improvements are needed regarding methodological clarity, conceptual consistency, grammar, reporting precision, and statistical explanation.

Major Strengths

- Timely and relevant topic addressing methodological quality in RA evidence synthesis
- Protocol registered in PROSPERO
- Use of AMSTAR 2 as an established methodological appraisal tool
- Planned multivariable and sensitivity analyses
- Independent screening and data extraction procedures clearly described

Major Weaknesses

- Several grammatical and language inconsistencies throughout the manuscript
- Methodological details require greater precision and clarity
- Insufficient justification for some exclusion criteria
- Conceptual overlap between methodological quality and risk of bias terminology
- Limited explanation of planned regression assumptions and sample size considerations
- Data availability statement may not fully align with PLOS protocol standards

Section-by-Section Comments

- *Abstract:* Clear structure but contains grammatical inconsistencies and overly broad claims.
- *Introduction:* Strong rationale but somewhat repetitive and occasionally overstated.
- *Methods:* Generally rigorous but requires clearer justification for exclusions and statistical procedures.
- *Database Searching:* Well described, though exact search strings should be provided in supplementary materials.
- *Study Selection*: Appropriate dual-reviewer approach.
- *Risk of Bias Assessment*: AMSTAR 2 procedures are appropriate but terminology should distinguish methodological quality from risk of bias.
- *Data Analysis*: Comprehensive approach, but regression assumptions and model stability require additional clarification.
- *Discussion:* Reasonable overview of study importance, though somewhat repetitive.
- *Conclusion:* Appropriate but should avoid overstating future impact.

Examples of Line-by-Line Shortcomings

- Line 20: 'associations between covariate and the methodological quality' should read 'associations between covariates and methodological quality.'
- Line 85: Missing space before '(PRISMA-P Group et al. 2015)'.
- Line 92: Section heading 'database searching' should be capitalized consistently.

• Line 149: 'detailed reason for judgement' should read 'detailed reasons for judgment.'
• Line 184: 'we will analysis association' should read 'we will analyze associations.'
• Line 193: 'R studio' should be standardized as 'RStudio.'
• Line 242: Data availability wording may not fully comply with PLOS protocol transparency requirements.

Recommendations for Authors

- Conduct thorough professional English-language editing
- Clarify regression assumptions and model justification
- Provide stronger rationale for exclusion criteria
- Standardize terminology related to methodological quality and risk of bias
- Improve consistency of formatting, capitalization, and reporting style
- Expand details on planned handling of missing data and model diagnostics
- Ensure data availability statement aligns with PLOS protocol requirements

Minor Revision.

This protocol addresses an important methodological gap in rheumatoid arthritis evidence synthesis and demonstrates several strengths in design and planning. However, revisions are required to improve methodological clarity, statistical explanation, language quality, and reporting consistency before publication.
