# Supplementary material for: Methodological quality of systematic reviews and meta-analyses of rheumatoid arthritis pharmacotherapy: A protocol for a meta-epidemiological study using AMSTAR 2
Source: PLoS One. 2026 Oct 6;21(10):e0359531. doi: 10.1371/journal.pone.0359531 (PMC13641359; doi:10.1371/journal.pone.0359531)
Supplement: Supplementary file 3 [file pone.0359531.s002.docx]

| **Search** | **Actions** | **Details** | **Query** | **Results** | **Time** |
| --- | --- | --- | --- | --- | --- |
| #4 |  |  | Search: **((("Arthritis, Rheumatoid"[MeSH] OR "rheumatoid arthritis"[tiab] OR "RA"[tiab])) AND (("Antirheumatic Agents"[MeSH] OR "drug therapy"[Subheading] OR "Methotrexate"[tiab] OR "Sulfasalazine"[tiab] OR "Leflunomide"[tiab] OR "Hydroxychloroquine"[tiab] OR "Adalimumab"[tiab] OR "Etanercept"[tiab] OR "Infliximab"[tiab] OR "Golimumab"[tiab] OR "Certolizumab"[tiab] OR "Tocilizumab"[tiab] OR "Anakinra"[tiab] OR "Abatacept"[tiab] OR "Rituximab"[tiab] OR "Tofacitinib"[tiab] OR "Baricitinib"[tiab] OR "Upadacitinib"[tiab] OR "Filgotinib"[tiab] OR "TNF inhibitors"[tiab] OR "JAK inhibitors"[tiab] OR "disease-modifying antirheumatic drugs"[tiab] OR "DMARDs"[tiab] OR "csDMARDs"[tiab] OR "bDMARDs"[tiab] OR "tsDMARDs"[tiab]))) AND (("Meta-Analysis"[Publication Type] OR "Systematic Review"[Publication Type] OR "meta-analysis"[tiab] OR "systematic review"[tiab] OR "meta-analyses"[tiab] OR "quantitative synthesis"[tiab] OR "pairwise meta-analysis"[tiab]))** | [2,375](https://pubmed.ncbi.nlm.nih.gov/?term=%28%28%28%22Arthritis%2C+Rheumatoid%22%5BMeSH%5D+OR+%22rheumatoid+arthritis%22%5Btiab%5D+OR+%22RA%22%5Btiab%5D%29%29+AND+%28%28%22Antirheumatic+Agents%22%5BMeSH%5D+OR+%22drug+therapy%22%5BSubheading%5D+OR+%22Methotrexate%22%5Btiab%5D+OR+%22Sulfasalazine%22%5Btiab%5D+OR+%22Leflunomide%22%5Btiab%5D+OR+%22Hydroxychloroquine%22%5Btiab%5D+OR+%22Adalimumab%22%5Btiab%5D+OR+%22Etanercept%22%5Btiab%5D+OR+%22Infliximab%22%5Btiab%5D+OR+%22Golimumab%22%5Btiab%5D+OR+%22Certolizumab%22%5Btiab%5D+OR+%22Tocilizumab%22%5Btiab%5D+OR+%22Anakinra%22%5Btiab%5D+OR+%22Abatacept%22%5Btiab%5D+OR+%22Rituximab%22%5Btiab%5D+OR+%22Tofacitinib%22%5Btiab%5D+OR+%22Baricitinib%22%5Btiab%5D+OR+%22Upadacitinib%22%5Btiab%5D+OR+%22Filgotinib%22%5Btiab%5D+OR+%22TNF+inhibitors%22%5Btiab%5D+OR+%22JAK+inhibitors%22%5Btiab%5D+OR+%22disease-modifying+antirheumatic+drugs%22%5Btiab%5D+OR+%22DMARDs%22%5Btiab%5D+OR+%22csDMARDs%22%5Btiab%5D+OR+%22bDMARDs%22%5Btiab%5D+OR+%22tsDMARDs%22%5Btiab%5D%29%29%29+AND+%28%28%22Meta-Analysis%22%5BPublication+Type%5D+OR+%22Systematic+Review%22%5BPublication+Type%5D+OR+%22meta-analysis%22%5Btiab%5D+OR+%22systematic+review%22%5Btiab%5D+OR+%22meta-analyses%22%5Btiab%5D+OR+%22quantitative+synthesis%22%5Btiab%5D+OR+%22pairwise+meta-analysis%22%5Btiab%5D%29%29&sort=) | 07:22:24 |
| #3 |  |  | Search: **("Meta-Analysis"[Publication Type] OR "Systematic Review"[Publication Type] OR "meta-analysis"[tiab] OR "systematic review"[tiab] OR "meta-analyses"[tiab] OR "quantitative synthesis"[tiab] OR "pairwise meta-analysis"[tiab])** | [630,734](https://pubmed.ncbi.nlm.nih.gov/?term=%28%22Meta-Analysis%22%5BPublication+Type%5D+OR+%22Systematic+Review%22%5BPublication+Type%5D+OR+%22meta-analysis%22%5Btiab%5D+OR+%22systematic+review%22%5Btiab%5D+OR+%22meta-analyses%22%5Btiab%5D+OR+%22quantitative+synthesis%22%5Btiab%5D+OR+%22pairwise+meta-analysis%22%5Btiab%5D%29&sort=) | 07:21:58 |
| #2 |  |  | Search: **("Antirheumatic Agents"[MeSH] OR "drug therapy"[Subheading] OR "Methotrexate"[tiab] OR "Sulfasalazine"[tiab] OR "Leflunomide"[tiab] OR "Hydroxychloroquine"[tiab] OR "Adalimumab"[tiab] OR "Etanercept"[tiab] OR "Infliximab"[tiab] OR "Golimumab"[tiab] OR "Certolizumab"[tiab] OR "Tocilizumab"[tiab] OR "Anakinra"[tiab] OR "Abatacept"[tiab] OR "Rituximab"[tiab] OR "Tofacitinib"[tiab] OR "Baricitinib"[tiab] OR "Upadacitinib"[tiab] OR "Filgotinib"[tiab] OR "TNF inhibitors"[tiab] OR "JAK inhibitors"[tiab] OR "disease-modifying antirheumatic drugs"[tiab] OR "DMARDs"[tiab] OR "csDMARDs"[tiab] OR "bDMARDs"[tiab] OR "tsDMARDs"[tiab])** | [3,081,456](https://pubmed.ncbi.nlm.nih.gov/?term=%28%22Antirheumatic+Agents%22%5BMeSH%5D+OR+%22drug+therapy%22%5BSubheading%5D+OR+%22Methotrexate%22%5Btiab%5D+OR+%22Sulfasalazine%22%5Btiab%5D+OR+%22Leflunomide%22%5Btiab%5D+OR+%22Hydroxychloroquine%22%5Btiab%5D+OR+%22Adalimumab%22%5Btiab%5D+OR+%22Etanercept%22%5Btiab%5D+OR+%22Infliximab%22%5Btiab%5D+OR+%22Golimumab%22%5Btiab%5D+OR+%22Certolizumab%22%5Btiab%5D+OR+%22Tocilizumab%22%5Btiab%5D+OR+%22Anakinra%22%5Btiab%5D+OR+%22Abatacept%22%5Btiab%5D+OR+%22Rituximab%22%5Btiab%5D+OR+%22Tofacitinib%22%5Btiab%5D+OR+%22Baricitinib%22%5Btiab%5D+OR+%22Upadacitinib%22%5Btiab%5D+OR+%22Filgotinib%22%5Btiab%5D+OR+%22TNF+inhibitors%22%5Btiab%5D+OR+%22JAK+inhibitors%22%5Btiab%5D+OR+%22disease-modifying+antirheumatic+drugs%22%5Btiab%5D+OR+%22DMARDs%22%5Btiab%5D+OR+%22csDMARDs%22%5Btiab%5D+OR+%22bDMARDs%22%5Btiab%5D+OR+%22tsDMARDs%22%5Btiab%5D%29+&sort=) | 07:21:30 |
| #1 |  |  | Search: **("Arthritis, Rheumatoid"[MeSH] OR "rheumatoid arthritis"[tiab] OR "RA"[tiab])** | [231,359](https://pubmed.ncbi.nlm.nih.gov/?term=%28%22Arthritis%2C+Rheumatoid%22%5BMeSH%5D+OR+%22rheumatoid+arthritis%22%5Btiab%5D+OR+%22RA%22%5Btiab%5D%29&sort=) | 07:20:18 |
