# Supplementary material for: Methodological quality of systematic reviews and meta-analyses of rheumatoid arthritis pharmacotherapy: A protocol for a meta-epidemiological study using AMSTAR 2
Source: PLoS One. 2026 Oct 6;21(10):e0359531. doi: 10.1371/journal.pone.0359531 (PMC13641359; doi:10.1371/journal.pone.0359531)
Supplement: Supplementary file 4 [file pone.0359531.s004.docx]

## Dear Editor,

## We sincerely thank the Editor and the reviewers for their thoughtful and constructive comments on our manuscript. These insightful suggestions have been highly instrumental in refining both the methodological framework and the conceptual presentation of our work. We have taken every comment seriously and have carefully revised the manuscript accordingly. In the following, we provide a detailed point-by-point response to each concern. All amendments are clearly highlighted in the revised manuscript for easy identification.

## We hope that the revised version now meets the standards of *plos one* and look forward to your further consideration.

Zhipeng Hu

Hospital of Chengdu university of traditional Chinese medicine

## **Reviewer #1**

### Original Comment (R1.1 - Search Strategy Appendix)

Search strategy Appendix: Although the authors refer to using “validated search filters” it would enhance the peer review process if they provided the complete search string for at least one database (e.g., PubMed) were included as a supplementary file now to verify the comprehensiveness of the MeSH terms.

**Response:** We fully agree with this suggestion. In the revised manuscript, we have added a new Supplementary Material 2 that provides the full search strategy for PubMed including exact MeSH/Emtree terms, free-text terms, Boolean operators, filters, and search dates. This ensures full compliance with the PRISMA-S checklist.

### Original Comment (R1.2 — Screening Software)

Screening Software: Could the authors specify which software they used for screening and deduplication, such as Rayyan, Covidence, or Excel? This information would illustrate how blinding was achieved between the two independent reviewers.

**Response:** Thank you for this practical suggestion. We have now specified in Section 2.3 (Study Selection) that records will be imported into EndNote for de-duplication and into Rayyan for screening. We also explicitly state that the Rayyan blind-on mode will be enabled to ensure that reviewers are masked to each other's decisions throughout the screening process.

### Original Comment (R1.3 — Inclusion of NMA)

Inclusion of NMA: The authors chose to exclude Network Meta-Analyses (NMA). While I understand the limitations of AMSTAR 2 in this regard, NMAs represent a significant portion of high-level evidence in RA. Therefore, a brief justification in the discussion regarding how this exclusion might affect the "comprehensiveness" of the quality map would be helpful.

**Response:** We appreciate this insightful comment. In the revised Limitations section (Discussion), we now provide a more substantive discussion of this issue. We explicitly acknowledge that excluding NMAs may limit the comprehensiveness of our evidence quality map, particularly for RA pharmacotherapy where NMAs are increasingly influential for comparative effectiveness assessments (e.g., head-to-head comparisons of JAK inhibitors vs. biologics). We also note that future methodological investigations should apply NMA-specific appraisal instruments (e.g., the ISPOR checklist or CINeMA) to address this gap.

### Original Comment (R1.4 - Core Outcome Sets)

Core Outcome Sets: It may be advantageous to identify if the data extraction would classify outcomes using recognized core sets (e.g., OMERACT) in order to ensure consistency across various reviews.

**Response:** This is a very helpful suggestion. We have now incorporated the OMERACT RA core outcome set into our protocol. In Section 2.4 (Inclusion and Exclusion Criteria, Outcomes), we have predefined the following expected primary outcomes: disease activity (DAS28, ACR20/50/70 response), physical function (HAQ), pain (VAS or other validated scales), radiographic progression (Sharp/van der Heijde-modified Sharp scores), and serious adverse events, explicitly noting that these correspond to the OMERACT RA core outcome set. Additionally, Section 2.5 (Data Extraction) states that extracted outcomes will be categorized according to the OMERACT RA core outcome set to ensure harmonized classification across included reviews.

## Reviewer #2

### Original Comment (R2.1 - Inconsistent Scope)

1. Inconsistent scope

The title, abstract, and objective focus on RA pharmacotherapy (csDMARDs, bDMARDs, tsDMARDs), while the inclusion criteria admit complementary therapies and lifestyle interventions without restriction. This is an internal contradiction that propagates throughout the manuscript. The authors must make an explicit choice — restrict to pharmacological interventions with a clear operational definition, or expand to all therapeutic interventions — and apply it consistently to the title, objective, abstract, search strategy, and sensitivity analyses.

**Response:** We fully agree and thank the reviewer for identifying this critical inconsistency. We have made the following revisions throughout the manuscript:

1. **Title** has been revised from “Methodological Assessment of Systematic Review and Meta-analysis in Rheumatoid Arthritis: A Protocol for Meta-epidemiological Research” to **“Methodological Quality of Systematic Reviews and Meta-analyses of Rheumatoid Arthritis Pharmacotherapy: A Protocol for a Meta-epidemiological Study Using AMSTAR 2”**, which now explicitly focuses on pharmacotherapy.
2. **Intervention criteria** (Section 2.4) now restrict inclusion to systematic reviews/meta-analyses whose primary aim is to evaluate RA pharmacotherapy (csDMARDs, bDMARDs, and tsDMARDs). Reviews evaluating exclusively complementary/alternative therapies, lifestyle interventions, physiotherapy, or surgical procedures are explicitly excluded.
3. For reviews that assess both pharmacological and non-pharmacological interventions, we have added a decision rule: inclusion will be considered only if pharmacotherapy constitutes the primary comparison and the majority of included primary studies evaluate drug interventions; such decisions will be recorded and tested in a sensitivity analysis.
4. The title, abstract, objective, search strategy, and sensitivity analyses have all been revised to consistently reflect this pharmacotherapy-focused scope.

### Original Comment (R2.2 - Mischaracterization of AMSTAR 2 Applicability)

1. Mischaracterization of AMSTAR 2 applicability

The exclusion of systematic reviews without quantitative synthesis, network meta-analyses, and IPD meta-analyses is justified by stating AMSTAR 2 is “not applicable.” This is partly incorrect: AMSTAR 2 can be applied to reviews without meta-analysis, with certain items treated as not applicable. The authors may legitimately restrict to pairwise quantitative syntheses as a *scope* decision, but must reframe the justification accordingly. The exclusion of network meta-analyses — the most policy-influential segment of RA pharmacotherapy evidence — demands explicit justification and a more substantive discussion of its implications.

**Response:** We sincerely thank the reviewer for this important correction. We have revised Section 2.4 (Study Design) to explicitly acknowledge that AMSTAR 2 can technically be applied to systematic reviews without meta-analysis (with certain items rated “not applicable”). We have reframed the exclusion of non-quantitative reviews as a **deliberate scope decision** rather than a claim of inapplicability. The revised text states:

“While AMSTAR 2 can technically be applied to systematic reviews without meta-analysis (with certain items rated 'not applicable'), we have elected as a deliberate scope decision to restrict inclusion to pairwise meta-analyses of RCTs or non-randomized intervention studies that have undergone quantitative synthesis. This restriction ensures comparability of AMSTAR 2 item ratings across reviews — particularly for Item 11 (appropriate meta-analytic methods) and Item 13 (accounting for risk of bias in interpretation) — and avoids heterogeneous 'not applicable' responses that would complicate our regression analysis. We explicitly acknowledge that this is a scope decision, not a claim that AMSTAR 2 is inapplicable to non-quantitative reviews.”

Regarding NMAs, we have also strengthened the discussion in the Limitations section acknowledging their importance and the implications of their exclusion, noting that “Future methodological investigations should apply NMA-specific appraisal instruments (e.g., the ISPOR checklist or CINeMA) to address this gap.”

### Original Comment (R2.3 - Circularity in the Regression Model)

1. Circularity in the regression model

Pre-registration is both an independent variable in the regression model and a direct contributor to AMSTAR 2 Item 2, which, in turn, feeds into the overall rating used as the dependent variable. This is incorporation bias. The authors should either remove pre-registration as a predictor of the composite AMSTAR 2 rating, conduct a secondary analysis using an AMSTAR 2 score recalculated after excluding Item 2, or, at a minimum, acknowledge this as a named limitation.

**Response:** We greatly appreciate this methodologically astute observation. We have redesigned our analytical approach in Section 2.7 (Data Analysis) to address this circularity. Specifically:

- Pre-registration will **not** be entered as a predictor of the composite AMSTAR 2 rating in the **primary model**.
- Instead, we will conduct a **pre-specified secondary analysis** in which the overall AMSTAR 2 rating is recalculated after excluding Item 2 (registered protocol) from the algorithm; pre-registration will then be reintroduced as a predictor in this secondary model.
- The exclusion of Item 2 from the primary model is explicitly acknowledged as a limitation, and results from both models will be reported transparently.

This two-model approach allows us to assess the association between pre-registration and non-protocol components of methodological quality without incorporation bias.

### Original Comment (R2.4 — Missing Inter-rater Reliability Plan and AMSTAR 2 Operationalization)

1. Missing inter-rater reliability plan and AMSTAR 2 operationalization

The protocol describes a dual-reviewer AMSTAR 2 assessment but provides no plan to quantify agreement (Cohen's kappa or ICC). This is the primary measurement of the study and cannot be omitted. Additionally, the handling of “partial yes” responses is unspecified, the seven critical AMSTAR 2 items are never named explicitly, and the overall rating terminology is inconsistent (“very low” is GRADE language; the correct AMSTAR 2 term is “critically low”). A dedicated paragraph should define the downgrading rules and list the seven critical items.

**Response:** We appreciate this thorough review of our methodological reporting. We have made the following additions in Section 2.6 (Methodological Quality Assessment Using AMSTAR 2):

1. **Inter-rater reliability**: We now specify that Cohen's kappa will be used for each item and a weighted kappa (or intraclass correlation coefficient, ICC) for the overall four-level rating, with interpretation thresholds (poor <0.40, moderate 0.40–0.60, good 0.61–0.80, very good >0.80).
2. **“Partial Yes” handling**: We now explicitly state that “Partial Yes” responses will be handled per Shea et al. (2017), counting as ‘Yes’ for the purpose of overall rating downgrading when the algorithm permits.
3. **Seven critical items**: We have explicitly listed all seven critical AMSTAR 2 items: Item 2 (registered protocol), Item 4 (adequacy of search), Item 7 (list of excluded studies with justification), Item 9 (risk-of-bias assessment of individual studies), Item 11 (appropriate meta-analytic methods), Item 13 (accounting for risk of bias in interpretation), and Item 15 (assessment of publication bias). A dedicated paragraph now describes the downgrading algorithm.
4. **Terminology**: We have corrected “Very Low” to **“Critically Low”** throughout the manuscript, consistent with AMSTAR 2 terminology.

### Original Comment (R2.5 - No Search Strategy Provided)

1. No search strategy provided

The complete search string for at least one database is absent, violating PRISMA-P Item 17. Citing Escobar Liquitay et al. as a filter source is not a substitute. Additionally, given the unrestricted language policy and the authors' institutional context (China), the absence of Chinese databases (CNKI, Wanfang) warrants justification, particularly if complementary therapies remain in scope.

**Response:** We fully agree. The following revisions have been made:

1. **Full search strategy**: The complete search strategy for PubMed, including exact terms, Boolean operators, filters, and search dates, is now provided in Supplementary Material 2.
2. **Chinese databases**: We have expanded our search to include three Chinese databases: China National Knowledge Infrastructure (CNKI), Wanfang Data Knowledge Service Platform, and China Science and Technology Journal Database (VIP). Additionally, given the revised scope focusing on pharmacotherapy, the inclusion of Chinese databases ensures comprehensive coverage of the RA pharmacotherapy literature published in Chinese-language journals.

### Original Comment (R2.6 - Regression Model Feasibility Not Addressed)

1. Regression model feasibility not addressed (both reviewers)

With nine covariates and a dependent variable expected to cluster heavily in the “critically low” category (72–90% in analogous published audits), the ordinal model risks instability and sparse cells. No sample-size estimate or power justification is provided. The authors must estimate the expected distribution of AMSTAR 2 ratings, pre-specify a rule for collapsing categories if cell counts are inadequate, and consider pre-specifying a hierarchy of predictors.

**Response:** We thank the reviewer for this important methodological concern. We have added a new section, **“2.8 Model Feasibility and Sample Size,”** which includes:

1. **Expected distribution**: We explicitly state, based on published methodological audits (Hammel et al. 2022; Karakasis et al. 2024), that we anticipate 70–90% of reviews will fall into the ‘Low’ or ‘Critically Low’ categories.
2. **Events-per-variable rule**: We apply the ≥10 reviews per predictor rule to the nine pre-specified covariates, targeting no fewer than 200 reviews for model stability.
3. **Collapsing rule**: We pre-specify that if any of the four rating categories contains <10 reviews, the dependent variable will be collapsed (e.g., ‘High/Moderate’ vs. ‘Low’ vs. ‘Critically Low’, or ‘Adequate’ [High/Moderate] vs. ‘Inadequate’ [Low/Critically Low]) and modeled with ordinal or binary logistic regression accordingly.
4. **Predictor hierarchy**: We pre-specify that the most theoretically important covariates (Cochrane source, intervention class, prospective registration, PRISMA adherence) will be entered in the primary model, with additional covariates added in a stepwise manner to assess model robustness.

### Original Comment (R2.7 - Eligibility Criteria Framed at the Wrong Level)

1. Eligibility criteria framed at the wrong level (Reviewer 2)

The PICO eligibility criteria describe *patients* (1987/2010 RA classification criteria), but the unit of analysis is *systematic reviews*. Many otherwise eligible reviews will include primary studies that use variable or outdated diagnostic definitions. Criteria should be reframed at the review level: “eligible reviews must have included patients with rheumatoid arthritis as defined by the review authors.”

**Response:** This is an excellent point. We have revised the Participants criterion (Section 2.4) to be framed at the review level:

**“Eligible reviews are those whose included population is patients with rheumatoid arthritis as defined by the review authors, irrespective of the specific classification criteria (e.g., 1987 ACR or 2010 ACR/EULAR) used by the primary studies. We frame this criterion at the review level: a review is eligible if its authors state that they included patients with RA, regardless of whether the underlying primary studies used the 1987 or 2010 criteria.”**

### Original Comment (R2 Additional Issues)

Additional Issues

- Section 2.6 title: “Risk of bias assessment” should be renamed “Methodological quality assessment using AMSTAR 2.” AMSTAR 2 is a critical appraisal tool, not a risk-of-bias instrument in the RoB 2/ROBINS-I sense.
- Forest plot: inappropriate for comparing item-level counts before/after AMSTAR 2 publication; replace with odds ratios or risk differences with confidence intervals. Replace the radar chart with stacked bar charts.
- Tense inconsistencies: several passages use past tense (“data were extracted,” “quality was assessed”), suggesting work may have already begun. If so, this must be disclosed transparently. The full methods section should be in the future tense for an unstarted protocol.
- Novelty claim: the assertion of being “the first” AMSTAR 2-based assessment of RA pharmacotherapy SRs is refuted by Shen et al. (2024, Front Pharmacol) and Ramalho et al. (2026, J Clin Med), neither of which is cited. The introduction requires revision.
- PROSPERO ID: CRD420261359521 appears to contain an extra digit; this should be verified.
- Minor: standardize “AMSTAR 2” spelling; correct “Cochrane origin”; resolve data availability inconsistency between the submission form and manuscript; align author contributions with the methods sections.

**Response — Section 2.6 title:** Agreed. We have renamed Section 2.6 to **“Methodological Quality Assessment Using AMSTAR 2”** and clarified that AMSTAR 2 is a critical-appraisal instrument, not a risk-of-bias tool in the RoB 2/ROBINS-I sense.

**Response — Forest plot / radar chart:** We have revised the analysis plan in Section 2.7 accordingly. Radar charts have been replaced with **stacked bar charts** for displaying the distribution of item ratings. Item-level adherence before vs. after the 2017 publication of AMSTAR 2 will be compared using **odds ratios (or risk differences) with 95% CIs**, rather than forest plots.

**Response — Tense inconsistencies:** We have carefully reviewed the entire manuscript and revised all methods sections to use the **future tense**, consistent with protocol reporting standards. The data extraction, quality assessment, and analysis sections now consistently use future-tense verbs (e.g., “will extract,” “will assess”).

**Response — Novelty claim:** We appreciate this important correction. We have revised the novelty claim throughout the manuscript. The Conclusion now states: “This study will provide a **comprehensive, cross-drug-class** AMSTAR 2-based methodological assessment of the RA pharmacotherapy evidence-synthesis field, **complementing prior appraisals limited to specific complementary or single-drug interventions**.” The Introduction has been similarly revised to acknowledge rather than dismiss prior work.

We have now added the following citations to the revised manuscript:

- Shen X, Liu X, Guo X, Hou X, Huang H, Feng Z. Systematic review of Janus kinases inhibitors for rheumatoid arthritis: methodology, reporting, and quality of evidence evaluation. Front Pharmacol. 2024;15:1459511. DOI: 10.3389/fphar.2024.1459511

- Ramalho B, Penedones A, Mendes D, Alves C. Quality of Systematic Reviews with Network Meta-Analyses on JAK Inhibitors in the Treatment of Rheumatoid Arthritis: Application of the AMSTAR 2 Scale. J Clin Med. 2026;15(2):725. DOI: 10.3390/jcm15020725

**Response — PROSPERO ID:**

Response: We have verified the PROSPERO registration and confirm that CRD420261359521 is correct. The registration is available at: https://www.crd.york.ac.uk/PROSPERO/view/CRD420261359521. We appreciate the reviewer's attention to detail.

**Response — Minor items:**

- “AMSTAR 2” spelling has been **standardized** throughout.
- “Cochrane origine” has been corrected to **“Cochrane review or not”** in the data extraction section.
- **Data availability** has been revised to: “No datasets were generated or analyzed during the current study. All relevant data from this study will be made available upon study completion and deposited in a public repository (Dryad).”
- **Author contributions** have been updated to reflect the revised team composition, including the addition of Delin Chen.

## Reviewer #3

### Original Comment (R3.1 - Rationale and Research Questions)

1. Rationale and Research Questions

The manuscript provides a valid rationale for conducting this study, and the need for a methodological assessment in this field is well justified. However, the research questions are not explicitly stated. While they can be inferred from the objectives, articulating them clearly would improve clarity and transparency.

**Response:** We have added a dedicated paragraph at the end of the Introduction explicitly stating **three research questions**:

(1) What is the overall AMSTAR 2 methodological quality of SRs/MAs of RA pharmacotherapy?
(2) Which study-level characteristics are associated with quality?
(3) Has quality changed after the 2017 publication of AMSTAR 2?

We also clarify that “as a meta-epidemiological study, the unit of analysis is the research study itself, examining associations between study-level characteristics and a study-level quality outcome rather than synthesizing clinical effects.”

### Original Comment (R3.2 - Technical Soundness and Study Design)

1. Technical Soundness and Study Design

The protocol is generally well designed and technically sound. The planned analyses, including ordinal logistic regression, assessment of proportional odds, multicollinearity diagnostics, and sensitivity analyses, are appropriate for the stated objectives.
However, several methodological aspects require clarification to ensure full robustness:

- The unit of analysis is not defined. It is unclear how updated versions of the same review or duplicate publications will be handled.
- The protocol does not describe how the team will manage systematic reviews containing multiple meta‑analyses or complex analytic structures.
- The rationale for selecting specific covariates in the regression model should be more explicitly justified within a conceptual framework.
- These issues do not undermine the feasibility of the study but should be addressed to strengthen methodological rigor.

**Response:** We thank Reviewer #3 for these detailed methodological suggestions. All three points have been addressed:

(a) The **unit of analysis** is now explicitly defined in Section 2.6: “The unit of analysis is the individual systematic review/meta-analysis.”

(b) We have added handling rules in Section 2.6: “For duplicate or updated versions of the same review, the most recent and most complete version will be retained; for reviews containing multiple meta-analyses, AMSTAR 2 will be applied at the review level.”

(c) The **rationale for covariate selection** is now more explicitly justified within a conceptual framework in Section 2.7. We have added intervention class as a covariate specifically to account for differing regulatory and reporting standards across drug classes (csDMARDs, bDMARDs, tsDMARDs), which may influence the methodological rigor of reviews. The addition of PRISMA adherence as an exposure variable was also informed by the published conceptual framework of review quality determinants.

### Original Comment (R3.3 - Replicability and Methodological Detail)

1. Replicability and Methodological Detail

Although the methodology is feasible, some elements are not described in sufficient detail to ensure full replicability:

- The protocol does not include a plan for assessing inter‑rater reliability (e.g., kappa or ICC) for AMSTAR 2 ratings, which is standard in methodological quality assessments.
- The handling of missing data is not fully described beyond contacting authors.
- The approach to reviews using non‑traditional meta‑analytic methods (e.g., Bayesian models, hierarchical models) is not specified.
- Providing additional detail in these areas would enhance reproducibility.

**Response:** All three points have been addressed:

(a) **Inter-rater reliability**: See response to R2.4. We now specify Cohen's kappa for each item and weighted kappa/ICC for the overall rating, with interpretation thresholds.

(b) **Missing data**: Section 2.5 now details a comprehensive missing data strategy: contact corresponding authors by email (up to two reminders over four weeks); if data remain unavailable, items will be scored conservatively with explicit documentation; a sensitivity analysis will be conducted excluding reviews with >20% missing data to assess robustness.

(c) **Non-traditional meta-analytic methods**: Section 2.7 now states: “Reviews using Bayesian or hierarchical pairwise meta-analytic models remain eligible. For such reviews, AMSTAR 2 items will be applied as applicable: Item 11 (appropriate meta-analytic methods) will be evaluated based on whether the Bayesian/hierarchical model was appropriately specified and justified; Item 13 (accounting for risk of bias) will be assessed using the same criteria as for frequentist meta-analyses. We will not exclude these reviews solely on the basis of their statistical approach.”

### Original Comment (R3.4 - Data Availability)

1. Data Availability

The manuscript states that all relevant data will be made available upon study completion. This is acceptable for a protocol, although the authors may consider specifying the intended repository or platform to improve transparency.

**Response:** We have revised the Data Availability section to specify: “All relevant data from this study will be made available upon study completion and deposited in a public repository **(Dryad)**.”

## Reviewer #4

### Original Comment (R4.1 - English Language Revision)

INTRODUCTION
The introduction is well-structured and provides a clear rationale for the study's objectives. However, the manuscript would benefit from a thorough English language revision to improve grammatical accuracy and clarity. There are several instances of subject-verb disagreement and missing relative pronouns that detract from the professional quality of the work.

**Response:** We have carefully revised the language throughout the manuscript. Specific grammatical issues identified by the reviewer have been corrected (see below), and a full language review has been performed to improve consistency and professional quality.

### Original Comment (R4.2 - Line 36)

Line 36
The phrase “A meta-analysis included 67 studies showed...” is grammatically incomplete. It should be revised to “A meta-analysis including 67 studies showed...” or “A meta-analysis that included 67 studies showed...”.

**Response:** Corrected to **“A meta-analysis that included 67 studies showed...”** (Introduction, first paragraph).

### Original Comment (R4.3 — Line 45)

Line 45
The subject “Therapeutic agents” is plural, but the verb used is “has”. This should be corrected to “have delivered”.

**Response:** Corrected to **“have delivered”** (Introduction, second paragraph).

### Original Comment (R4.4 - Line 38)

Line 38
The text mentions the “GDB study”; please confirm if this refers to the GBD (Global Burden of Disease) study, as this is a common typographical error.

**Response:** Thank you for catching this typographical error. Corrected to **“GBD (Global Burden of Disease) study”** (Introduction, first paragraph).

### Original Comment (R4.5 - Line 54)

Line 54
“precision presentation” would be more idiomatic as “precise presentation.”

**Response:** Corrected to **“precise presentation”** (Introduction). The revised text now reads: “Systematic reviews must provide a precise presentation of current knowledge.”

### Original Comment (R4.6 - Line 66)

Line 66
“To-date” should be written as “to date” when used in this context.

**Response:** The sentence containing this instance has been restructured in the revision. The revised Introduction now reads: “no prior study has provided a comprehensive, cross-drug-class methodological assessment” - removing the “to-date” phrasing entirely.

### Original Comment (R4.7 - Database Searching)

DATABASE SEARCHING
According to PRISMA-P, the authors should present a full search strategy for at least one database. Please include the full search strategy for at least PubMed (in the main text or as an appendix), with exact terms, operators, and filters applied.

**Response:** Addressed - see response to R1.1 and R2.5. PubMed strategy was provided in Supplementary Material 2 as an example.

### Original Comment (R4.8 - Study Selection)

STUDY SELECTION
The authors should specify the software used for duplicate removal and for the screening process (e.g., EndNote, Rayyan, Covidence). Additionally, the protocol should describe the procedure to be followed if contacting study authors is necessary, for instance, to retrieve missing data or clarify eligibility.

**Response:** Both points addressed. Screening software (EndNote for deduplication, Rayyan with blind-on mode for screening) is specified in Section 2.3. The contacting-authors procedure is described in Section 2.5: up to two email reminders over four weeks, with a conservative scoring rule for unreported data and a sensitivity analysis excluding reviews with >20% missing data.

### Original Comment (R4.9 - Inclusion/Exclusion Criteria: Non-pharmacological Treatments)

INCLUSION AND EXCLUSION CRITERIA

1. The manuscript explicitly defines its objective in the abstract and introduction as assessing the methodological quality of systematic reviews and meta-analyses regarding “RA pharmacotherapy.” However, the inclusion criteria permit non-pharmacological treatments, which contradicts the stated aim of the study and introduces significant clinical and methodological heterogeneity. Systematic reviews of lifestyle or alternative therapies often possess fundamentally different baseline methodological qualities, funding structures, and publication standards compared to those evaluating targeted synthetic or biological DMARDs. I recommend either revising the study objective to encompass all RA interventions or restricting the inclusion criteria strictly to pharmacotherapy to ensure the logical coherence of the analyzed data.

**Response:** Addressed - see response to R2.1. We have restricted inclusion criteria strictly to pharmacotherapy (csDMARDs, bDMARDs, tsDMARDs) and revised title, abstract, objective, and all subsequent sections to consistently reflect this scope.

### Original Comment (R4.10 - Inclusion/Exclusion Criteria: Outcomes Vagueness)

1. The inclusion criteria related to outcomes are too vague and may lead to inconsistencies between reviewers during screening. The authors should provide at least a list of expected primary outcomes. Additionally, it should be clarified whether cost-effectiveness studies reporting clinical outcomes are also excluded.

**Response:** We have added a list of expected primary outcomes based on the OMERACT RA core outcome set (see R1.4): disease activity (DAS28, ACR20/50/70), physical function (HAQ), pain (VAS), radiographic progression (Sharp/vdH-modified Sharp scores), and serious adverse events. We have also clarified that health-economic studies reporting only economic outcomes without clinical effectiveness data are excluded, unless clinical-effectiveness data are embedded within the same review.

### Original Comment (R4.11 - Inclusion/Exclusion Criteria: Observational Studies)

1. Furthermore, the exclusion of NMA, IPD meta-analyses, and realist reviews based on AMSTAR 2 applicability is well justified (although it is a limitation and possibly undermines the study's primary objective to provide a comprehensive assessment of RA pharmacotherapy). However, the authors should clarify whether meta-analyses of observational studies are eligible for inclusion.

**Response:** We have explicitly stated in Section 2.4 (Study Design) that meta-analyses synthesizing observational intervention studies (e.g., cohort studies, case-control studies) are eligible, provided they have conducted quantitative synthesis. This decision will be recorded and tested in a sensitivity analysis by comparing reviews based on RCTs only vs. those including observational studies.

### Original Comment (R4.12 - Data Extraction: Tense and Redundancy)

DATA EXTRACTION
Two minor issues should be addressed. First, the section inconsistently mixes past tense with future tense, suggesting the text may have been partially adapted from a published article. As this is a protocol, all verbs should be consistently written in the future tense. Second, the role of the third reviewer in resolving discrepancies is mentioned twice redundantly and should be consolidated into a single statement.

**Response:** All verbs in the data extraction section now consistently use **future tense**. The redundant mention of the third reviewer has been consolidated into a single statement.

### Original Comment (R4.13 - ROB Assessment: Future Tense)

ROB ASSESSMENT
As previously outlined, all verbs should be written in the future tense.

**Response:** Addressed. Section 2.6 now consistently uses future tense throughout.

### Original Comment (R4.14 - Data Analysis: Geographic Region Criteria)

DATA ANALYSIS

1. The protocol lists the geographic region of the corresponding author, categorized as a developing or developed country, as an independent variable for the regression analysis. However, the methodology does not specify the objective criteria or classification system that will be used to define these categories.

**Response:** We have specified in Section 2.7 that this will be classified using the **World Bank income classification** (high-income vs. non-high-income economies), based on the country's classification in the year of publication.

### Original Comment (R4.15 - Data Analysis: Circularity of Preregistration)

1. The proposed regression analysis includes preregistration as an independent variable to predict the overall AMSTAR 2 quality rating. However, protocol registration is explicitly evaluated as Item 2 in the AMSTAR 2 tool and is designated as a critical domain. Because the absence of preregistration deterministically downgrades the overall AMSTAR 2 confidence rating to “Low” or “Critically Low,” including it as an independent predictor introduces severe circularity into the model. I recommend that the authors remove preregistration from the list of independent covariates, or, at least, test the model with or without this variable.

**Response:** Addressed - see response to R2.3. We have removed pre-registration from the primary model and added a secondary analysis recalculating AMSTAR 2 after excluding Item 2.

### Original Comment (R4.16 - Data Analysis: Missing Intervention Type Covariate)

1. On the other hand, the proposed model omits the type of intervention as a covariate. Given that systematic reviews of newer biological agents are often subject to more stringent recent regulatory and reporting standards compared to older conventional therapies or lifestyle interventions, the intervention type can be a major potential confounder for methodological quality. I suggest adding intervention category as an independent variable in the primary regression model.

**Response:** We fully agree and have added **intervention class** (csDMARDs only / bDMARDs only / tsDMARDs only / mixed / other pharmacotherapy) as a covariate in the regression model. The rationale is stated explicitly in Section 2.7: this accounts for differing regulatory and reporting standards across drug classes, which may influence the methodological rigor of reviews.

## Reviewer #5

### Original Comment (R5.1 - Line 184)

I found this publication well structured, clear and addresses a significant gap of literature regarding the quality of evidence in Rheumatoid Arthritis (RA) pharmacotherapy. The use of AMSTAR 2 is appropriate for this objective.

Anyhow I had found a minor correction.

On page 10, line 184: “we will analysis association”, should be corrected to “we will analyze the association.”

**Response:** We thank Reviewer #5 for the positive assessment and for noting this error. Corrected. Section 2.7 now reads: **“we will analyze the associations between each independent variable and the methodological-quality rating.”**

## Reviewer #6

### Original Comment (R6.1 - Title)

I have carefully reviewed the manuscript. The authors propose and register a protocol for a meta-epidemiological study assessing the methodological quality of systematic reviews and meta-analyses conducted on rheumatoid arthritis treatments, particularly pharmacotherapy. The study aims to identify overall methodological trends in the literature and investigate associations between predefined covariates and study quality. Overall, the protocol is well designed, and the major methodological components have been appropriately addressed. The PRISMA-P checklist has been completed carefully, providing a strong basis for a rigorous and transparent study design. I appreciate the authors' efforts and genuinely believe that this protocol is suitable for publication in its current form. However, I have a few minor suggestions:

Regarding the title, the authors may consider explicitly mentioning the concept of treatment, particularly pharmacotherapy, to better reflect the scope of the study and improve discoverability for readers.

**Response:** We sincerely thank Reviewer #6 for the positive evaluation. We agree with the suggestion regarding the title. The revised title now reads: **“Methodological Quality of Systematic Reviews and Meta-analyses of Rheumatoid Arthritis Pharmacotherapy: A Protocol for a Meta-epidemiological Study Using AMSTAR 2”**, which explicitly mentions pharmacotherapy and improves both scope reflection and discoverability.

### Original Comment (R6.2 - Long Sentences in Introduction)

Some sentences in the Introduction are excessively long and may be difficult to follow (e.g., lines 49-53).

**Response:** We have carefully revised the Introduction to improve readability. Long sentences have been broken into shorter, clearer segments. For example, the compound sentence describing the roles of systematic reviews has been restructured for clarity.

### Original Comment (R6.3 - Repetitive Statements in Methods)

In the Methods section, some statements appear repetitive, particularly in lines 97–99 and 109–110. The authors may consider revising these sections for conciseness.

**Response:** We have reviewed the Methods section and consolidated redundant statements, particularly in the data extraction section where the third reviewer's role in resolving discrepancies was mentioned twice.

## Reviewer #7

### Full Review

**Title:** Methodological Assessment of Systematic Review and Meta-analysis in Rheumatoid Arthritis: A Protocol for Meta-epidemiological Research

**Recommendation:** Minor Revision

**Overall Evaluation**

This manuscript presents a protocol for a meta-epidemiological study aiming to assess the methodological quality of systematic reviews and meta-analyses on rheumatoid arthritis (RA) pharmacotherapy using the AMSTAR 2 tool. The topic is timely and important given the rapid expansion of evidence synthesis studies in RA treatment. The protocol demonstrates several methodological strengths, including protocol registration, predefined covariates, and planned sensitivity analyses. However, improvements are needed regarding methodological clarity, conceptual consistency, grammar, reporting precision, and statistical explanation.

**Major Strengths**

- Timely and relevant topic addressing methodological quality in RA evidence synthesis
- Protocol registered in PROSPERO
- Use of AMSTAR 2 as an established methodological appraisal tool
- Planned multivariable and sensitivity analyses
- Independent screening and data extraction procedures clearly described

**Major Weaknesses**

- Several grammatical and language inconsistencies throughout the manuscript
- Methodological details require greater precision and clarity
- Insufficient justification for some exclusion criteria
- Conceptual overlap between methodological quality and risk of bias terminology
- Limited explanation of planned regression assumptions and sample size considerations
- Data availability statement may not fully align with PLOS protocol standards

**Section-by-Section Comments**

- Abstract: Clear structure but contains grammatical inconsistencies and overly broad claims.
- Introduction: Strong rationale but somewhat repetitive and occasionally overstated.
- Methods: Generally rigorous but requires clearer justification for exclusions and statistical procedures.
- Database Searching: Well described, though exact search strings should be provided in supplementary materials.
- Study Selection: Appropriate dual-reviewer approach.
- Risk of Bias Assessment: AMSTAR 2 procedures are appropriate but terminology should distinguish methodological quality from risk of bias.
- Data Analysis: Comprehensive approach, but regression assumptions and model stability require additional clarification.
- Discussion: Reasonable overview of study importance, though somewhat repetitive.
- Conclusion: Appropriate but should avoid overstating future impact.

**Examples of Line-by-Line Shortcomings**

- Line 20: ‘associations between covariate and the methodological quality’ should read ‘associations between covariates and methodological quality.’
- Line 85: Missing space before ‘(PRISMA-P Group et al. 2015)’.
- Line 92: Section heading 'database searching' should be capitalized consistently.
- Line 149: ‘detailed reason for judgement’ should read ‘detailed reasons for judgment.’
- Line 184: ‘we will analysis association’ should read ‘we will analyze associations.'’
- Line 193: ‘R studio’ should be standardized as ‘RStudio.’
- Line 242: Data availability wording may not fully comply with PLOS protocol transparency requirements.

**Recommendations for Authors**

- Conduct thorough professional English-language editing
- Clarify regression assumptions and model justification
- Provide stronger rationale for exclusion criteria
- Standardize terminology related to methodological quality and risk of bias
- Improve consistency of formatting, capitalization, and reporting style
- Expand details on planned handling of missing data and model diagnostics
- Ensure data availability statement aligns with PLOS protocol requirements

Minor Revision.

This protocol addresses an important methodological gap in rheumatoid arthritis evidence synthesis and demonstrates several strengths in design and planning. However, revisions are required to improve methodological clarity, statistical explanation, language quality, and reporting consistency before publication.

**Response:** We sincerely thank Reviewer #7 for this thorough, structured, and constructive review. Every point raised has been addressed in the revised manuscript. Below we map each weakness, line-by-line issue, and recommendation to specific revisions:

#### Major Weaknesses — Addressed

| Weakness | How Addressed |
| --- | --- |
| Several grammatical and language inconsistencies | Full language revision conducted; see responses to R4.1–R4.6 and line-by-line fixes below |
| Methodological details require greater precision and clarity | Unit of analysis defined (Section 2.6); duplicate/updated review handling added (Section 2.6); inter-rater reliability added (Section 2.6); missing data strategy added (Section 2.5); Bayesian/hierarchical model handling added (Section 2.7) |
| Insufficient justification for some exclusion criteria | Exclusion criteria reframed as scope decisions with detailed rationale (Section 2.4); NMA/IPD-MA exclusion discussed in Limitations |
| Conceptual overlap between methodological quality and risk of bias | Section 2.6 renamed to “Methodological Quality Assessment Using AMSTAR 2”; explicit distinction added between critical appraisal and risk-of-bias tools |
| Limited explanation of regression assumptions and sample size | New Section 2.8 (Model Feasibility and Sample Size) added; proportional odds assumption testing specified |
| Data availability statement alignment with PLOS standards | Updated to specify Dryad repository |

#### Line-by-Line Shortcomings — Addressed

| Line | Original Issue | Revised Wording |
| --- | --- | --- |
| Line 20: | “associations between covariate and the methodological quality” | Corrected to “associations between covariates and methodological quality” (Abstract) |
| Line 85: | Missing space before “(PRISMA-P Group et al. 2015)” | Space added: “Protocols (PRISMA-P Group et al. 2015)” |
| Line 92: | “database searching” | Capitalized to “Database Searching” (Section 2.2) |
| Line 149: | “detailed reason for judgement” | Revised to “We will provide detailed justification for each item rating in supplementary materials” (Section 2.6) |
| Line 184: | “we will analysis association” | Corrected to “we will analyze the associations” (Section 2.7) |
| Line 193: | “R studio” | Standardized to “R (Version 4.3.1) with the RStudio integrated development environment (Version 2023.06.2)” (Section 2.7) |
| Line 242: | Data availability wording | Revised to specify Dryad repository |

#### Section-by-Section Comments — Addressed

| Section | Issue | Revision |
| --- | --- | --- |
| Abstract | Grammatical inconsistencies and overly broad claims | Language corrected; claim softened from “first” to “comprehensive, cross-drug-class...complementing prior appraisals” |
| Introduction | Repetitive and occasionally overstated | Streamlined; novelty claims revised |
| Methods | Requires clearer justification | Exclusion criteria reframed; statistical procedures elaborated |
| Database Searching | Search strings needed | Full strategies added as Supplementary Material 2 |
| Study Selection | Appropriate — no change needed | Enhanced with EndNote/Rayyan details |
| Risk of Bias Assessment | Terminology overlap | Section renamed; AMSTAR 2 vs. RoB distinction clarified |
| Data Analysis | Assumptions need clarification | New Section 2.8; Brant test specified |
| Discussion | Somewhat repetitive | Streamlined for conciseness |
| Conclusion | Overstated future impact | Revised to measured language |

## Reviewer #8

### Original Comment

Respected authors, despite the fact that it is a good protocol, I fail to understand how it is a Meta-Epidemiological Study. It is only a review study assessing the strengths and weaknesses of the research methods applied in previous systematic reviews and meta-analyses.

**Response:** We appreciate this comment, which gives us an opportunity to clarify our study design. A meta-epidemiological study is defined as research that uses systematic review methods to investigate the design, conduct, analysis, or reporting of a collection of related research studies (Murad and Wang 2017). The defining characteristic is that the **unit of analysis is the research study itself** rather than the individual patient or participant.

In our protocol:

1. The unit of analysis is each systematic review/meta-analysis, not individual patients.
2. We examine associations between **study-level characteristics** (e.g., prospective registration, funding source, Cochrane origin, intervention class) and a **study-level quality outcome** (AMSTAR 2 rating).
3. We are not synthesizing clinical treatment effects but rather investigating methodological patterns across a body of evidence.

We recognize that this definition may not be universally familiar. In the revised manuscript, we have added a clarifying sentence at the end of the Introduction that explicitly states: **“Crucially, this is a meta-epidemiological investigation: the unit of analysis is the systematic review itself, and the outcome of interest is methodological quality, rather than the synthesis of pooled clinical effect estimates from primary trials (Murad and Wang 2017).”** We hope this clarifies the nature of our study.

## Manuscript Change Checklist

| # | Revision | Location |
| --- | --- | --- |
| 1 | Title revised to focus on RA pharmacotherapy | Title page |
| 2 | Author list updated (Delin Chen added) | Author list |
| 3 | Three explicit research questions added | Introduction (new paragraph) |
| 4 | Meta-epidemiological study definition clarified | Introduction |
| 5 | Inclusion criteria restricted to pharmacotherapy | Section 2.4 Intervention |
| 6 | Participants reframed at review level | Section 2.4 Participants |
| 7 | Expected outcomes predefined (OMERACT core set) | Section 2.4 Outcomes |
| 8 | Observational-study MA eligibility clarified | Section 2.4 Study design |
| 9 | NMA/IPD-MA exclusion reframed as scope decision | Section 2.4 + Discussion |
| 10 | Chinese databases added (CNKI, Wanfang, VIP) | Section 2.2 |
| 11 | Full search strategies in Supplementary Material 2 | Section 2.2 |
| 12 | EndNote + Rayyan blind-on screening specified | Section 2.3 |
| 13 | Missing data strategy with sensitivity analysis | Section 2.5 |
| 14 | Section 2.6 renamed (methodological quality, not RoB) | Section 2.6 |
| 15 | Inter-rater reliability (Cohen's kappa, ICC) added | Section 2.6 |
| 16 | Seven critical AMSTAR 2 items explicitly listed | Section 2.6 |
| 17 | “Very Low” → “Critically Low” throughout | Section 2.6 +full text |
| 18 | “Partial Yes” handling specified | Section 2.6 |
| 19 | Duplicate/updated review handling rules | Section 2.6 |
| 20 | Regression model redesigned (no incorporation bias) | Section 2.7 |
| 21 | Intervention class added as covariate | Section 2.7 |
| 22 | World Bank classification for geographic region | Section 2.7 |
| 23 | Bayesian/hierarchical model handling specified | Section 2.7 |
| 24 | Radar chart → stacked bar; forest plot → OR/RD | Section 2.7 |
| 25 | New Section 2.8: Model Feasibility and Sample Size | Section 2.8 (new) |
| 26 | Novelty claim softened throughout | Abstract, Introduction, Discussion |
| 27 | Language revision (grammar, tense, terminology) | Full text |
| 28 | Data availability statement updated (Dryad) | Data Availability |
| 29 | Author contributions updated | Author Contributions |
| 30 | All line-by-line corrections (see R7 table) | Multiple locations |
