# Supplementary material for: Methodological quality of systematic reviews and meta-analyses of rheumatoid arthritis pharmacotherapy: A protocol for a meta-epidemiological study using AMSTAR 2
Source: PLoS One. 2026 Oct 6;21(10):e0359531. doi: 10.1371/journal.pone.0359531 (PMC13641359; doi:10.1371/journal.pone.0359531)
Supplement: Supplementary file 5 [file pone.0359531.s005.docx]

**Response to Reviewers**

Manuscript ID: PONE-D-26-18105R1

Title: Methodological Quality of Systematic Reviews and Meta-analyses of Rheumatoid Arthritis Pharmacotherapy: A Protocol for a Meta-epidemiological Study Using AMSTAR 2
Corresponding author: Zhipeng Hu (545053309@qq.com)

Dear Editors,

We thank you and the four reviewers for the careful and constructive evaluation of our manuscript. All comments have been addressed point by point, and the manuscript has been substantially revised. In addition to the specific revisions detailed below, the main changes are:

1. Abstract: completely restructured: a single, unified Methods paragraph (six databases), the explicit Objective restored, and the duplicated text block removed.

2. Regression framework redesigned (Sections 2.7-2.8): binary primary analysis (“Adequate” vs. “Inadequate”), a parsimonious primary model with five parameters, Firth penalized logistic regression as the pre-specified default estimator, a formal sample-size plan (pmsampsize with Peduzzi cross-check; N ≥ 300 target), a three-tier analysis ladder, and seven pre-specified sensitivity analyses.

3. Pharmacotherapy scope clarified (Section 2.4): biosimilars classified under bDMARDs; reviews primarily evaluating glucocorticoids, NSAIDs, or intra-articular agents excluded; the “other pharmacotherapy” category removed from the model.

4. AMSTAR 2: multi-meta-analysis rating rules specified (Section 2.6), together with the corrected “Partial Yes” logic and the explicit four-level downgrading algorithm.

5. References: reformatted according to ICMJE recommendations, with three duplicate pairs merged and four methodological references added (now 22 references, all cited in text).

6. PROSPERO registration (CRD420261359521): updated during peer review to reflect the revised methods, with all amendments documented in the registration history.

7. Qifan Cao added as co-first author (see Authorship Changes below).

We also confirm the following three statements:

Authorship Changes. Relative to the original submission, the author list has changed as follows: Dr. Delin Chen has been added (he joined the study at the first revision), Dr. Qifan Cao has been added as the second author and co-first author with Dr. Maoyi Yang, and Dr. Yang Liu has withdrawn from the authorship for personal reasons (at the first revision), with Dr. Liu’s conceptual contribution now acknowledged in the manuscript. The additions are based on substantial contributions to the revised manuscript: Dr. Chen conceived and led the drafting of the first-round revision responding to the reviewers’ comments, and Dr. Cao conceived and drafted the current second-round revision; Dr. Cao’s study responsibilities (literature searching, study screening, and participation in data analysis and in drafting the future manuscript reporting the completed study) are stated in the Author Contributions. All authorship changes have been approved in writing by all authors, including Dr. Liu, and are being formally declared through the authorship change form accompanying this revision. The changes are reflected in the title page (co-first-author statement) and the Author Contributions section of the revised manuscript.

PROSPERO Update. The registration record (CRD420261359521) has been updated during peer review to reflect the revised methods presented in this version of the protocol; all amendments are documented in the registration history. This is now stated transparently in Section 2.1 of the revised manuscript.

Data Availability. The Data Availability statement designates the Dryad repository for deposition of all relevant data upon study completion, and the corresponding field in the submission form has been updated to match the manuscript text.

Our point-by-point responses follow. Reviewer comments are reproduced in italics, and all quoted text is taken verbatim from the revised manuscript.

**point-by-point responses**

Journal Requirements

*Requirement 1: If the reviewer comments include a recommendation to cite specific previously published works, please review and evaluate these publications to determine whether they are relevant and should be cited. There is no requirement to cite these works unless the editor has indicated otherwise*.

**Response**: None of the reviewers recommended the citation of specific previously published works. The four references added in this revision (references 18-21) were added on our own initiative to support newly introduced methodological content - Firth penalized regression (18), Bayesian ordinal regression via brms (19), the pmsampsize sample-size approach (20), and the events-per-parameter rule (21) - and are cited at the relevant methods sections.

*Requirement 2: Please review your reference list to ensure that it is complete and correct. … Any changes to the reference list should be mentioned in the rebuttal letter that accompanies your revised manuscript.*

**Response**: We have carefully reviewed and corrected the reference list. The changes are as follows:

1. Three pairs of duplicate entries with identical DOIs have been merged, leaving a single entry for each: Shea et al. 2017, Rodríguez-García et al. 2021, and Karakasis et al. 2024.

2. Four references have been added to support the newly introduced statistical methods: Firth 1993 (reference 18, bias-reduced penalized logistic regression); Bürkner 2017 (reference 19, brms R package for Bayesian ordinal regression); Riley et al. 2020 (reference 20, sample size for prediction models, pmsampsize); Peduzzi et al. 1996 (reference 21, events-per-parameter simulation study).

3. The entire list has been reformatted according to the ICMJE Recommendations, with references numbered in order of first citation in the text (see also our response to Reviewer #8).

4. The revised list contains 22 references, all of which are cited in the text and none of which is orphaned.

5. No retracted publications are cited.

Reviewer #1

*Recommendation: Accept (Re-review Satisfied / Minor Formatting Correction Required).*

**Response**: We sincerely thank Reviewer #1 for the thorough and positive assessment of the first revision, and for identifying the Abstract formatting error. Both action items raised have been corrected, as detailed below.

Reviewer #1, Comment 1 (Abstract)

*Copy-Paste Error: The revised Methods text was accidentally pasted directly into the Background section (lines 13–20). This resulted in severe text duplication, the complete disappearance of the Objective section, and a redundant repetition of “the Cochrane Library” (lines 13–14). Database Inconsistency: The newly added Chinese databases (CNKI, Wanfang, VIP) appear in the duplicated Background section but were omitted from the actual Methods paragraph (lines 21–26). Action Required: The editorial team/production should ask the authors to clean up the Abstract structure (restoring the explicit Objective statement and removing the duplicated text block).*

*Response*: We apologize for this error, which occurred during text replacement in the first revision. The Abstract has been completely restructured: the duplicated block has been removed, the explicit Objective statement restored, and the six databases are now listed consistently in the single Methods paragraph. The revised Abstract now reads:

Changes in the manuscript (Abstract, Objective and Methods):

Objective: This protocol describes a meta-epidemiological study that will systematically appraise the methodological quality of published systematic reviews and meta-analyses of RA pharmacotherapy (csDMARDs, bDMARDs, and tsDMARDs) using the AMSTAR 2 instrument and explore study-level factors associated with methodological quality.

Methods: We will systematically search PubMed, Embase, the Cochrane Library and three Chinese databases-China National Knowledge Infrastructure (CNKI), Wanfang Data and China Science and Technology Journal Database-from inception to June 1, 2026. Two independent reviewers will screen studies, extract data and assess methodological quality using the AMSTAR 2 instrument; an overall rating (high, moderate, low, critically low) will be derived for each review. Binary logistic regression comparing “Adequate” (High/Moderate) versus “Inadequate” (Low/Critically Low) reviews will be used for the primary analysis, with ordinal logistic regression fitted as a pre-specified exploratory analysis; pre-specified sensitivity and robustness analyses (including penalized and Bayesian estimation for sparse categories) will test the stability of findings.

Reviewer #1, Comment 2 (Minor note on “Critically Low”)

*Minor note: The term “Critically Low” should be verified for consistent usage in Section 2.6, replacing one lingering instance of “Very Low”.*

**Response**: We have verified the entire manuscript. All four-level ratings now read “High, Moderate, Low, or Critically Low,” and the term “Very Low” no longer appears anywhere in the manuscript, including Section 2.6:

Changes in the manuscript (Section 2.6):

Each item will be judged as “Yes”, “Partial Yes”, or “No”; an overall rating of High, Moderate, Low, or Critically Low will be derived using the AMSTAR 2 algorithm.

Reviewer #2

*The authors have addressed all major first-round concerns satisfactorily. … The manuscript is close to acceptable. The following issues require correction before publication:*

**Response**: We thank Reviewer #2 for the positive overall assessment and for the precise identification of the remaining issues. All eight points have been addressed as follows.

Reviewer #2, Comment 1 (Abstract structural duplication)

*The abstract contains a structural duplication: a free-standing Methods paragraph listing six databases appears before the Background heading, while the formal Methods field lists only three. One unified, accurate paragraph should replace both.*

**Response**: As detailed in our response to Reviewer #1 (Comment 1), the Abstract has been completely restructured into a single unified set of fields: the duplicated pre-Background block was removed, the explicit Objective statement was restored, and the Methods field now lists all six databases (PubMed, Embase, the Cochrane Library, CNKI, Wanfang Data, and the China Science and Technology Journal Database). Please see the full revised Abstract text quoted in our response to Reviewer #1, Comment 1.

Reviewer #2, Comment 2 (Internal contradiction on pre-registration in Section 2.8)

*Section 2.7 correctly excludes pre-registration from the primary model to avoid incorporation bias, but Section 2.8 then lists “prospective registration” among the primary model’s theoretically important covariates. Pre-registration must be removed from Section 2.8’s primary predictor hierarchy and retained only in the secondary model.*

**Response**: We thank the reviewer for catching this contradiction introduced during the first revision. The predictor hierarchy in Section 2.8 has been rewritten: the primary model now contains only three covariates (Cochrane source, intervention class, PRISMA adherence), the remaining six covariates are examined only in secondary models, and prospective registration is explicitly retained only in the secondary model after exclusion of Item 2:

Changes in the manuscript (Section 2.8):

The primary model will contain three theoretically important covariates-Cochrane systematic review source (binary), intervention class (categorical, four levels), and PRISMA adherence (continuous). … The remaining six pre-specified exposures and covariates will be examined only in secondary models, and no stepwise variable selection will be performed, to avoid overfitting and multiple-testing inflation. Prospective registration will not be entered as a predictor in the primary model (see Section 2.7) and will be retained only in the secondary model after exclusion of Item 2.

Reviewer #2, Comment 3 (Scope of eligible pharmacotherapy)

*Eligibility criteria restrict to csDMARDs/bDMARDs/tsDMARDs, yet the regression model includes an “other pharmacotherapy” category and sensitivity analysis 3 implies additional drug classes may be eligible. The authors must explicitly state whether glucocorticoids, NSAIDs, biosimilars, and intra-articular pharmacological agents are in or out.*

**Response**: We have made the scope fully explicit in Section 2.4. Biosimilars are classified under the corresponding bDMARD category and remain eligible; reviews whose primary aim is exclusively glucocorticoids, NSAIDs, or intra-articular pharmacological agents are excluded; and the “other pharmacotherapy” category has been deleted from the model, which is now four-level. Boundary decisions for mixed pharmacological/non-pharmacological reviews are recorded and tested in sensitivity analysis 5:

Changes in the manuscript (Section 2.4, Intervention):

For reviews that assess both pharmacological and non-pharmacological interventions, inclusion will be considered only if pharmacotherapy constitutes the primary comparison and the majority of included primary studies evaluate drug interventions; such decisions will be recorded and tested in a sensitivity analysis (Section 2.7, analysis 5). … For scope clarity, biosimilars of biological DMARDs will be classified under the corresponding bDMARD category and remain eligible; reviews whose primary aim is exclusively the evaluation of glucocorticoids, non-steroidal anti-inflammatory drugs, or intra-articular pharmacological agents in RA will be excluded, as these lie outside the csDMARDs/bDMARDs/tsDMARDs framework central to this study.

Changes in the manuscript (Section 2.7):

The model also includes intervention class (csDMARDs only / bDMARDs only / tsDMARDs only / mixed, where "mixed" denotes reviews evaluating more than one DMARD class) as a covariate, added to account for differing regulatory and reporting standards across drug classes, which may influence the methodological rigor of reviews.

Reviewer #2, Comment 4 (“Partial Yes” logic and explicit downgrading rules)

*The current wording - “counting as Yes for the purpose of overall rating downgrading” - is the inverse of the algorithm’s logic: it is a “No” on a critical item, not a “Yes,” that triggers downgrading. The sentence should state that a "Partial Yes" on a critical item does not constitute a critical weakness and therefore does not trigger downgrading, consistent with the AMSTAR 2 algorithm. Additionally, the downgrading rules should be stated explicitly: no or one non-critical weakness = High; more than one non-critical weakness = Moderate; one critical flaw = Low; more than one critical flaw = Critically Low.*

**Response**: We agree, and the sentence has been rewritten to match the AMSTAR 2 algorithm, with the four downgrading rules now stated explicitly:

Changes in the manuscript (Section 2.6):

In accordance with the AMSTAR 2 algorithm (6), a “Partial Yes” on a critical item does not constitute a critical weakness and therefore does not by itself trigger downgrading; only “No” responses are counted as weaknesses. The overall rating is derived as follows: High-no or only one non-critical weakness; Moderate-more than one non-critical weakness; Low-one critical weakness with or without non-critical weaknesses; Critically Low-more than one critical weakness with or without non-critical weaknesses.

Reviewer #2, Comment 5 (Sensitivity analysis 1 naming)

*Sensitivity analysis 1 still reads “high/medium vs. low/very low” - this should be “High/Moderate vs. Low/Critically Low.”*

**Response**: The sensitivity-analysis section has been rewritten in full, and the old list (including the “high/medium vs. low/very low” wording) has been removed. Because the primary analysis itself is now the binary comparison of “Adequate” (High/Moderate) versus “Inadequate” (Low/Critically Low) reviews, that former dichotomization has been promoted from a sensitivity analysis to the primary analysis. Sensitivity analysis 1 now tests an alternative three-category collapse of the four-level outcome:

Changes in the manuscript (Section 2.7):

Our primary analysis will model methodological quality as a binary outcome-“Adequate” (overall rating High or Moderate) versus “Inadequate” (Low or Critically Low)-using binary logistic regression. … To test the robustness of the results, seven pre-specified sensitivity analyses will be conducted: (1) the four-level outcome will be collapsed alternatively into three categories (High/Moderate vs. Low vs. Critically Low) and re-analyzed where cell counts permit; …

Reviewer #2, Comment 6 (Past tense in the Introduction)

*Introduction line 94 uses past tense (“we conducted a comprehensive methodological appraisal”), which is inappropriate for an unstarted protocol and raises a transparency concern about whether data collection has begun.*

**Response**: We agree. The sentence has been corrected to the future tense, and the tense of the entire manuscript has been checked for consistency with the protocol status (no data collection has begun; all study activities are expressed with “will”):

Changes in the manuscript (Introduction, final paragraph):

To bridge this critical evidence gap, we will conduct a comprehensive methodological appraisal of published SRs/MAs on RA pharmacotherapy using the AMSTAR 2 tool.

Reviewer #2, Comment 7 (Citation misattributions and duplicate references)

*Shea et al. 2017 is misattributed as a prior quality appraisal of complementary therapies (it is the AMSTAR 2 tool paper), and Rodriguez-García et al. 2021 is described as a JAK inhibitor study (it concerns intra-articular therapies). Both require correction. Additionally, three reference pairs are duplicated in the reference list (Karakasis 2024a/b, Rodriguez-García 2021a/b, Shea 2017a/b — identical DOIs) and should be merged.*

**Response**: We apologize for these attribution errors, which have been corrected. Shea et al. 2017 is now cited only as the AMSTAR 2 tool paper, and the three prior appraisal domains are now attributed to the correct references: complementary/alternative therapies to Li et al. (7), single-agent JAK inhibitors to Shen et al. (8), and intra-articular therapies to Rodríguez-García et al. (9, the overview of intra-articular therapies). The three duplicate pairs have been merged into single entries (see also Journal Requirement 2 above).

Changes in the manuscript (Introduction):

Accordingly, methodological appraisal tools (AMSTAR 2) have been developed to ensure transparent, standardized, and rigorous conduct of systematic reviews (6). [Reference 6 is Shea et al. 2017, the AMSTAR 2 tool paper.]

To our knowledge, while previous AMSTAR 2-based appraisals have focused on specific complementary/alternative therapies (7), single-agent JAK inhibitors (8), or intra-articular therapies (9), no prior study has provided a cross-drug-class methodological assessment covering the full spectrum of csDMARDs, bDMARDs, and tsDMARDs with regression-based factor analysis.

Reviewer #2, Comment 8 (Minor items)

*Minor items: “MeSH/Emtree” in reference to the PubMed strategy should read “MeSH terms” (Emtree is Embase-specific); “to-date” with a hyphen reappears in the Discussion; “comprehensive… standardized comprehensive” contains a word repetition; the data availability field in the submission form does not mention the Dryad repository, inconsistently with the manuscript text.*

**Response**: All four items have been corrected:

1. “MeSH/Emtree” now reads “MeSH terms” in both places where the search strategy is described; “Emtree” has been deleted from the manuscript.

2. The hyphenated “to-date” has been corrected in the Discussion.

3. The word repetition “standardized comprehensive” has been removed.

4. The Data Availability field in the submission form has been updated to mention the Dryad repository, consistent with the manuscript text.

Changes in the manuscript (Section 2.2):

We will use a combined strategy of MeSH terms and free text words and literature related to systematic reviews will be searched using specifically developed and validated search filters without restrictions on publication date or language (12). … The full search strategy for PubMed, including exact MeSH terms, free-text terms, Boolean operators, filters, and search dates, is provided in Supplementary Material 2, in accordance with PRISMA-S.

Changes in the manuscript (Discussion):

To date, no study has explored potential factors influencing methodological quality.

Changes in the manuscript (Discussion):

… conduct a comprehensive, large-scale, standardized assessment of the methodological quality of systematic reviews and meta-analyses related to drug treatments for RA …

Changes in the manuscript (Data Availability statement):

No datasets were generated or analyzed during the current study. All relevant data from this study will be made available upon study completion and deposited in a public repository (Dryad).

Reviewer #5

Reviewer #5, Comment 1 (Single rating for reviews containing multiple meta-analyses)

*You state that for reviews containing multiple meta-analyses, “AMSTAR 2 will be applied at the review level.” If a single systematic review contains multiple meta-analyses-some of which adhere to high reporting standards and some of which do not-how will you derive a single, valid AMSTAR 2 rating for the review?*

**Response**: We thank the reviewer for this important question. Section 2.6 now pre-specifies an explicit, deterministic rule that removes operator discretion: review-level items are rated for the review as a whole, whereas meta-analysis-level items are rated according to the meta-analysis of the lowest quality (i.e., the most conservative rating across the included meta-analyses). The number of meta-analyses within each review and any within-review inconsistency are recorded and reported, and sensitivity analysis 4 excludes reviews containing multiple meta-analyses to test the robustness of this rule:

Changes in the manuscript (Section 2.6):

To derive a single valid rating for a review containing multiple meta-analyses, review-level items (e.g., Items 2, 4, 7, 9) will be rated for the review as a whole, whereas meta-analysis-level items (e.g., Items 11, 13, 14, 15) will be rated conservatively: when the included meta-analyses differ, the item will be rated according to the meta-analysis of the lowest quality, to avoid operator discretion and to ensure comparability across reviews. The number of meta-analyses within each review and any within-review inconsistency will be recorded in the extraction form and reported in supplementary tables.

Reviewer #5, Comment 2 (Power and overfitting under a skewed outcome)

*You anticipate that 70-90% of reviews will fall into the “Low” or “Critically Low” categories, yet you intend to run a regression model with nine covariates. Even with 200 reviews, if your data are heavily skewed (e.g., 180 reviews in the “Low/Critically Low” categories and only 20 in the “High/Moderate” categories), your regression model will likely lack the power to detect significant associations and will be prone to overfitting.*

**Response**: We agree with this assessment, and the analysis plan has been redesigned as a coherent chain of pre-specified safeguards, each addressing a specific link in the concern the reviewer raises:

1. Outcome dichotomized to maximize stability under the expected skew. The primary analysis models “Adequate” (High/Moderate) versus “Inadequate” (Low/Critically Low) as a binary outcome; the four-level ordinal model has been demoted to a pre-specified exploratory analysis that is fitted only when every collapsed category contains at least 10 reviews.

2. Parsimonious primary model: three covariates, five parameters. Only Cochrane source, intervention class (four levels), and PRISMA adherence enter the primary model; counting parameters rather than variables, the four-level intervention class contributes three indicators, so k = 5. The remaining six pre-specified covariates are examined only in secondary models, and no stepwise selection is performed.

3. Formal sample-size planning. As a meta-epidemiological study, this investigation is a census of the available evidence base: the realized sample size will be determined by the number of eligible reviews identified. Based on a conservative PubMed search (title/abstract) that retrieves more than 2400 records, we anticipate at least 300 eligible reviews. We will therefore verify that this anticipated scale satisfies the statistical requirements of the primary model by formally estimating the minimum sample size required for stable regression using the pmsampsize package (20), with the Peduzzi rule (N = 10k/p) as a cross-check; if the realized sample or event count falls short, the pre-specified analysis ladder (Section 2.8) applies. The formal pmsampsize calculation will be performed prior to data analysis and reported with the final manuscript.

4. Estimation matched to the anticipated events-per-parameter ratio. With a 10-15% expected proportion of Adequate reviews, N ≥ 300 yields 30-45 events in the smaller group, i.e., 30–45 ÷ 5 = 6–9 events per parameter, below the conventional threshold of 10. Firth’s bias-reduced penalized logistic regression with penalized profile-likelihood confidence intervals is therefore the pre-specified default estimator; unpenalized maximum likelihood will additionally be reported only if the realized ratio reaches 10.

5. Direct answer to the reviewer’s scenario (e.g., 20 Adequate vs. 180 Inadequate reviews). In such a realized dataset the events-per-parameter ratio would be 20 ÷ 5 = 4, and the pre-specified plan responds automatically: Firth estimation is applied, the analysis ladder is triggered, and — if the realized Adequate proportion falls below 10% - all regression results are interpreted as exploratory, with the descriptive appraisal remaining the principal contribution.

6. Bayesian robustness check for sparse ordinal categories. A Bayesian ordinal regression with weakly informative priors (brms) is pre-specified to provide shrinkage against overfitting when the exploratory ordinal model is fitted.

7. A three-tier analysis ladder that adapts the model to the realized sample size, and a priori power calculations reported with the final model.

Changes in the manuscript (Section 2.8):

We therefore set N ≥ 300 included reviews as the target, and we will estimate the required minimum sample size formally using the pmsampsize package (20), with the Peduzzi rule (N = 10k/p, where k is the number of predictors and p the proportion in the smaller outcome group) as a cross-check (21).

The primary model will contain three theoretically important covariates-Cochrane systematic review source (binary), intervention class (categorical, four levels), and PRISMA adherence (continuous). Counting model parameters rather than variables, the four-level intervention class contributes three indicator variables, so the model contains five parameters in total; with N ≥ 300 and a 10-15% expected proportion of Adequate reviews (30-45 events in the smaller outcome group), the events-per-parameter ratio is 30-45 ÷ 5 = 6-9, below the conventional threshold of 10 (21). The primary analysis will therefore be estimated using Firth's bias-reduced penalized logistic regression with penalized profile-likelihood confidence intervals, as pre-specified in Section 2.7; unpenalized maximum-likelihood estimates will additionally be reported if the realized events-per-parameter ratio reaches 10.

Changes in the manuscript (Section 2.8, analysis ladder):

To avoid dependence on any single realized sample size, we pre-specify the following analysis ladder. If 200–299 reviews are included, the three-predictor primary model is retained, with the events-per-parameter ratio recomputed for the observed distribution (Firth estimation applying as above). If 100–199 reviews are included, the primary model is reduced to the two most theoretically important covariates (Cochrane source and PRISMA adherence), and the remaining covariates are analyzed univariately. If fewer than 100 reviews are included, regression is abandoned in favor of the descriptive methodological-quality appraisal. … The realized sample size and the analysis tier adopted will be reported with the PRISMA flow diagram, and a priori power calculations based on the anticipated event rate in the least frequent rating category will be reported to demonstrate whether the final model is adequately powered, with the number of predictors adjusted to the available sample. If the realized proportion of Adequate reviews is very low (e.g., <10%), the regression results will be interpreted as exploratory, as acknowledged in the Limitations section.

\## Reviewer #8

Reviewer #8, Comment 1 (References per ICMJE guidelines)

*You have made enormous improvements by incorporating the reviewers recommendations. Kindly, also adjust the references as per ICMJE guidelines.*

**Response**: We thank the reviewer for the encouraging assessment and this suggestion. The reference list has been reformatted according to the ICMJE Recommendations: references are numbered and cited in order of first appearance in the text; when there are more than six authors, the first six are listed followed by “et al.”; journal titles are abbreviated in NLM style; and the year, volume(issue), page range, and DOI are provided for each entry. For example:

Examples of the reformatted entries (References 1 and 6):

1. Gravallese EM, Firestein GS. Rheumatoid Arthritis - Common Origins, Divergent Mechanisms. N Engl J Med. 2023 Feb 9;388(6):529–42. doi:10.1056/NEJMra2103726

2. Shea BJ, Reeves BC, Wells G, Thuku M, Hamel C, Moran J, et al. AMSTAR 2: a critical appraisal tool for systematic reviews that include randomised or non-randomised studies of healthcare interventions, or both. BMJ. 2017 Sep 21;358:j4008. doi:10.1136/bmj.j4008

The complete revised list (22 references) appears in the References section of the revised manuscript; all reference-list changes are declared in our response to Journal Requirement 2 above.

We are grateful to the academic editor and all four reviewers for their time and expertise. We believe that the revised manuscript - with its restructured Abstract, clarified scope, corrected AMSTAR 2 rating logic, and redesigned, power-aware analysis plan - has fully addressed all of the raised concerns, and we hope it is now suitable for publication in PLOS ONE. All changes are highlighted in the marked-up copy (“Revised Manuscript with Track Changes”), and a clean version is provided separately.

Sincerely,

Zhipeng Hu, on behalf of all authors

Hospital of Chengdu University of Traditional Chinese Medicine, Chengdu, Sichuan, China

545053309@qq.com
