# Supplementary material for: Methodological quality of systematic reviews and meta-analyses of rheumatoid arthritis pharmacotherapy: A protocol for a meta-epidemiological study using AMSTAR 2
Source: PLoS One. 2026 Oct 6;21(10):e0359531. doi: 10.1371/journal.pone.0359531 (PMC13641359; doi:10.1371/journal.pone.0359531)
Supplement: Peer review history

# Peer review: Decision Letter 0

**DOI:** 10.1371/journal.pone.0359531.r001

**Authors:** Athanasios G. Pantelis

19 May 2026

Dear Dr. hu,

Thank you for submitting your manuscript to PLOS ONE. After careful consideration, we feel that it has merit but does not fully meet PLOS ONE’s publication criteria as it currently stands. Therefore, we invite you to submit a revised version of the manuscript that addresses the points raised during the review process.

==============================

**ACADEMIC EDITOR: Please address all reviewers' comments before resubmitting your manuscript for evaluation to PLOS ONE.**

==============================

Please submit your revised manuscript by Jul 03 2026 11:59PM. If you will need more time than this to complete your revisions, please reply to this message or contact the journal office at plosone@plos.org. When you're ready to submit your revision, log on to https://www.editorialmanager.com/pone/ and select the 'Submissions Needing Revision' folder to locate your manuscript file.

- A letter that responds to each point raised by the academic editor and reviewer(s). You should upload this letter as a separate file labeled 'Response to Reviewers'.
- A marked-up copy of your manuscript that highlights changes made to the original version. You should upload this as a separate file labeled 'Revised Manuscript with Track Changes'.
- An unmarked version of your revised paper without tracked changes. You should upload this as a separate file labeled 'Manuscript'.

If you would like to make changes to your financial disclosure, please include your updated statement in your cover letter. Guidelines for resubmitting your figure files are available below the reviewer comments at the end of this letter.

If applicable, we recommend that you deposit your laboratory protocols in protocols.io to enhance the reproducibility of your results. Protocols.io assigns your protocol its own identifier (DOI) so that it can be cited independently in the future. For instructions see: https://journals.plos.org/plosone/s/submission-guidelines#loc-laboratory-protocols. Additionally, PLOS ONE offers an option for publishing peer-reviewed Lab Protocol articles, which describe protocols hosted on protocols.io. Read more information on sharing protocols at https://plos.org/protocols?utm_medium=editorial-email&utm_source=authorletters&utm_campaign=protocols.

As the corresponding author, your ORCID iD is verified in the submission system and will appear in the published article. PLOS supports the use of ORCID, and we encourage all coauthors to register for an ORCID iD and use it as well. Please encourage your coauthors to verify their ORCID iD within the submission system before final acceptance, as unverified ORCID iDs will not appear in the published article. *Only*  the individual author can complete the verification step; PLOS staff *cannot*  verify ORCID iDs on behalf of authors.

We look forward to receiving your revised manuscript.

Kind regards,

Athanasios G. Pantelis

Academic Editor

PLOS One

Journal Requirements:

When submitting your revision, we need you to address these additional requirements.

1. Please ensure that your manuscript meets PLOS ONE's style requirements, including those for file naming. The PLOS ONE style templates can be found at

https://journals.plos.org/plosone/s/file?id=wjVg/PLOSOne_formatting_sample_main_body.pdf and https://journals.plos.org/plosone/s/file?id=ba62/PLOSOne_formatting_sample_title_authors_affiliations.pdf

2. PLOS requires an ORCID iD for the corresponding author in Editorial Manager on papers submitted after December 6th, 2016. Please ensure that you have an ORCID iD and that it is validated in Editorial Manager. To do this, go to ‘Update my Information’ (in the upper left-hand corner of the main menu), and click on the Fetch/Validate link next to the ORCID field. This will take you to the ORCID site and allow you to create a new iD or authenticate a pre-existing iD in Editorial Manager.

3. Please provide a complete Data Availability Statement in the submission form, ensuring you include all necessary access information or a reason for why you are unable to make your data freely accessible. If your research concerns only data provided within your submission, please write "All data are in the manuscript and/or supporting information files" as your Data Availability Statement.

4. Please include captions for your Supporting Information files at the end of your manuscript, and update any in-text citations to match accordingly. Please see our Supporting Information guidelines for more information: http://journals.plos.org/plosone/s/supporting-information.

5. If the reviewer comments include a recommendation to cite specific previously published works, please review and evaluate these publications to determine whether they are relevant and should be cited. There is no requirement to cite these works unless the editor has indicated otherwise.

[Note: HTML markup is below. Please do not edit.]

Reviewers' comments:

Reviewer's Responses to Questions

**Comments to the Author**

1. Does the manuscript provide a valid rationale for the proposed study, with clearly identified and justified research questions?

Reviewer #1: Yes

Reviewer #2: Yes

Reviewer #3: Partly

Reviewer #4: Yes

Reviewer #5: Yes

Reviewer #6: Yes

Reviewer #7: Yes

Reviewer #8: Yes

**********

2. Is the protocol technically sound and planned in a manner that will lead to a meaningful outcome and allow testing the stated hypotheses?

Reviewer #1: Yes

Reviewer #2: Partly

Reviewer #3: Partly

Reviewer #4: Yes

Reviewer #5: Yes

Reviewer #6: Yes

Reviewer #7: Yes

Reviewer #8: Yes

**********

3. Is the methodology feasible and described in sufficient detail to allow the work to be replicable?

Reviewer #1: Yes

Reviewer #2: Yes

Reviewer #3: No

Reviewer #4: Yes

Reviewer #5: Yes

Reviewer #6: Yes

Reviewer #7: Yes

Reviewer #8: Yes

**********

4. Have the authors described where all data underlying the findings will be made available when the study is complete?

The PLOS Data policy

Reviewer #1: Yes

Reviewer #2: No

Reviewer #3: Yes

Reviewer #4: Yes

Reviewer #5: Yes

Reviewer #6: No

Reviewer #7: No

Reviewer #8: Yes

**********

5. Is the manuscript presented in an intelligible fashion and written in standard English?

Reviewer #1: Yes

Reviewer #2: Yes

Reviewer #3: Yes

Reviewer #4: No

Reviewer #5: Yes

Reviewer #6: Yes

Reviewer #7: Yes

Reviewer #8: Yes

**********

Please use the space provided to explain your answers to the questions above and, if applicable, provide comments about issues authors must address before this protocol can be accepted for publication. You may also include additional comments for the author, including concerns about research or publication ethics.

You may also provide optional suggestions and comments to authors that they might find helpful in planning their study.

Reviewer #1: PEER REVIEW REPORT

Manuscript ID: PONE-D-26-18105

Title: Methodological Assessment of Systematic Review and Meta-analysis in Rheumatoid

Arthritis: A Protocol for Meta-epidemiological Research

Reviewer Recommendation: Minor Revision

1- General Comments:

The meta-epidemiological study protocol is methodologically rigorous and well-designed.

In this study, the authors focus on the methodological quality of systematic reviews (SRs) and

meta-analyses (MAs) related to rheumatoid arthritis (RA) treatments in the Rheumatology

literature.

Using AMSTAR2 and ordinal logistic regression (ORL) in conjunction with sensitivity analysis

is a robust and sophisticated statistical approach.

2- Major Strengths:

● Methodological accuracy: PRISMA-P and PRISMA-S guidelines are clearly followed

and complied with, and this is much appreciated.

● Transparency: The reproducibility of the study is enhanced through the inclusion of a

list of excluded studies along with detailed justifications for AMSTAR2 ratings in the

supplementary materials.

● Statistical Depth: A high level of statistical proficiency is demonstrated by the inclusion

of the variance inflation factor (VIF) as a measure of multicollinearity and the adoption

of Ridge Regression as an alternative model.

3- Points for Clarification (Minor Issues):

Search strategy Appendix: Although the authors refer to using "validated search filters"

it would enhance the peer review process if they provided the complete search string for

at least one database (e.g., PubMed) were included as a supplementary file now to verify

the comprehensiveness of the MeSH terms.

Screening Software: Could the authors specify which software they used for screening

and deduplication, such as Rayyan, Covidence, or Excel? This information would

illustrate how blinding was achieved between the two independent reviewers.

Inclusion of NMA: The authors chose to exclude Network Meta-Analyses (NMA).

While I understand the limitations of AMSTAR 2 in this regard, NMAs represent a

significant portion of high-level evidence in RA. Therefore, a brief justification in the discussion regarding how this exclusion might affect the "comprehensiveness" of the

quality map would be helpful.

Core Outcome Sets: It may be advantageous to identify if the data extraction would

classify outcomes using recognized core sets (e.g., OMERACT) in order to ensure

consistency across various reviews.

4- Conclusion: Overall, I believe this protocol is of superior quality and reveals a clear roadmap

for a critically needed investigation. Nevertheless, addressing the minor flaws mentioned above

would strengthen the manuscript and demonstrate its eligibility for publishing.

5. Confidential Comments to the Editor:

I have used AI-assisted tools (QuillBot and Wordtune) solely to refine the English

phrasing and organizing my technical notes, as English is my second language. All scientific evaluations,

critiques of the methodology, and final assessments of the manuscript's validity are based entirely on my

own major and professional expertise in molecular diagnostics and laboratory sciences.

Reviewer #2: Major Issues

1. Inconsistent scope

The title, abstract, and objective focus on RA pharmacotherapy (csDMARDs, bDMARDs, tsDMARDs), while the inclusion criteria admit complementary therapies and lifestyle interventions without restriction. This is an internal contradiction that propagates throughout the manuscript. The authors must make an explicit choice — restrict to pharmacological interventions with a clear operational definition, or expand to all therapeutic interventions — and apply it consistently to the title, objective, abstract, search strategy, and sensitivity analyses.

2. Mischaracterization of AMSTAR 2 applicability

The exclusion of systematic reviews without quantitative synthesis, network meta-analyses, and IPD meta-analyses is justified by stating AMSTAR 2 is "not applicable." This is partly incorrect: AMSTAR 2 can be applied to reviews without meta-analysis, with certain items treated as not applicable. The authors may legitimately restrict to pairwise quantitative syntheses as a *scope* decision, but must reframe the justification accordingly. The exclusion of network meta-analyses — the most policy-influential segment of RA pharmacotherapy evidence — demands explicit justification and a more substantive discussion of its implications.

3. Circularity in the regression model

Pre-registration is both an independent variable in the regression model and a direct contributor to AMSTAR 2 Item 2, which, in turn, feeds into the overall rating used as the dependent variable. This is incorporation bias. The authors should either remove pre-registration as a predictor of the composite AMSTAR 2 rating, conduct a secondary analysis using an AMSTAR 2 score recalculated after excluding Item 2, or, at a minimum, acknowledge this as a named limitation.

4. Missing inter-rater reliability plan and AMSTAR 2 operationalization

The protocol describes a dual-reviewer AMSTAR 2 assessment but provides no plan to quantify agreement (Cohen's kappa or ICC). This is the primary measurement of the study and cannot be omitted. Additionally, the handling of "partial yes" responses is unspecified, the seven critical AMSTAR 2 items are never named explicitly, and the overall rating terminology is inconsistent ("very low" is GRADE language; the correct AMSTAR 2 term is "critically low"). A dedicated paragraph should define the downgrading rules and list the seven critical items.

5. No search strategy provided

The complete search string for at least one database is absent, violating PRISMA-P Item 17. Citing Escobar Liquitay et al. as a filter source is not a substitute. Additionally, given the unrestricted language policy and the authors' institutional context (China), the absence of Chinese databases (CNKI, Wanfang) warrants justification, particularly if complementary therapies remain in scope.

6. Regression model feasibility not addressed (both reviewers)

With nine covariates and a dependent variable expected to cluster heavily in the "critically low" category (72–90% in analogous published audits), the ordinal model risks instability and sparse cells. No sample-size estimate or power justification is provided. The authors must estimate the expected distribution of AMSTAR 2 ratings, pre-specify a rule for collapsing categories if cell counts are inadequate, and consider pre-specifying a hierarchy of predictors.

7. Eligibility criteria framed at the wrong level (Reviewer 2)

The PICO eligibility criteria describe *patients* (1987/2010 RA classification criteria), but the unit of analysis is *systematic reviews*. Many otherwise eligible reviews will include primary studies that use variable or outdated diagnostic definitions. Criteria should be reframed at the review level: "eligible reviews must have included patients with rheumatoid arthritis as defined by the review authors."

Additional Issues

- Section 2.6 title: "Risk of bias assessment" should be renamed "Methodological quality assessment using AMSTAR 2." AMSTAR 2 is a critical appraisal tool, not a risk-of-bias instrument in the RoB 2/ROBINS-I sense.

- Forest plot: inappropriate for comparing item-level counts before/after AMSTAR 2 publication; replace with odds ratios or risk differences with confidence intervals. Replace the radar chart with stacked bar charts.

- Tense inconsistencies: several passages use past tense ("data were extracted," "quality was assessed"), suggesting work may have already begun. If so, this m:ust be disclosed transparently. The full methods section should be in the future tense for an unstarted protocol.

- Novelty claim: the assertion of being "the first" AMSTAR 2-based assessment of RA pharmacotherapy SRs is refuted by Shen et al. (2024, Front Pharmacol) and Ramalho et al. (2026, J Clin Med), neither of which is cited. The introduction requires revision.

- PROSPERO ID: CRD420261359521 appears to contain an extra digit; this should be verified.

- Minor standardize "AMSTAR 2" spelling; correct "Cochrane origin"; resolve data availability inconsistency between the submission form and manuscript; align author contributions with the methods sections.

Reviewer #3: The protocol addresses an important and timely topic: the methodological quality of systematic reviews and meta‑analyses in rheumatoid arthritis pharmacotherapy. The rapid expansion of evidence syntheses in this field makes a meta‑epidemiological assessment both relevant and valuable. The manuscript demonstrates a solid understanding of methodological standards and uses appropriate tools such as AMSTAR 2, PRISMA‑P, and PRISMA‑S.

Below I provide detailed comments to support further strengthening of the protocol.

Major Comments

1. Rationale and Research Questions

The manuscript provides a valid rationale for conducting this study, and the need for a methodological assessment in this field is well justified. However, the research questions are not explicitly stated. While they can be inferred from the objectives, articulating them clearly would improve clarity and transparency.

2. Technical Soundness and Study Design

The protocol is generally well designed and technically sound. The planned analyses, including ordinal logistic regression, assessment of proportional odds, multicollinearity diagnostics, and sensitivity analyses, are appropriate for the stated objectives.

However, several methodological aspects require clarification to ensure full robustness:

-The unit of analysis is not defined. It is unclear how updated versions of the same review or duplicate publications will be handled.

-The protocol does not describe how the team will manage systematic reviews containing multiple meta‑analyses or complex analytic structures.

-The rationale for selecting specific covariates in the regression model should be more explicitly justified within a conceptual framework.

-These issues do not undermine the feasibility of the study but should be addressed to strengthen methodological rigor.

3. Replicability and Methodological Detail

Although the methodology is feasible, some elements are not described in sufficient detail to ensure full replicability:

-The protocol does not include a plan for assessing inter‑rater reliability (e.g., kappa or ICC) for AMSTAR 2 ratings, which is standard in methodological quality assessments.

-The handling of missing data is not fully described beyond contacting authors.

-The approach to reviews using non‑traditional meta‑analytic methods (e.g., Bayesian models, hierarchical models) is not specified.

-Providing additional detail in these areas would enhance reproducibility.

4. Data Availability

The manuscript states that all relevant data will be made available upon study completion. This is acceptable for a protocol, although the authors may consider specifying the intended repository or platform to improve transparency.

Reviewer #4: This manuscript presents a protocol for a methodological quality assessment of meta-analyses on RA pharmacotherapy using the AMSTAR 2 tool, a timely and relevant contribution to the evidence synthesis field. I have a few comments that I hope the authors will find useful in strengthening the protocol.

INTRODUCTION

The introduction is well-structured and provides a clear rationale for the study’s objectives. However, the manuscript would benefit from a thorough English language revision to improve grammatical accuracy and clarity. There are several instances of subject-verb disagreement and missing relative pronouns that detract from the professional quality of the work.

Line 36

The phrase “A meta-analysis included 67 studies showed..." is grammatically incomplete. It should be revised to "A meta-analysis including 67 studies showed..." or "A meta-analysis that included 67 studies showed...".

Line 45

The subject "Therapeutic agents" is plural, but the verb used is "has". This should be corrected to "have delivered".

Line 38

The text mentions the "GDB study"; please confirm if this refers to the GBD(Global Burden of Disease) study, as this is a common typographical error.

Line 54

"precision presentation" would be more idiomatic as "precise presentation."

Line 66

“To-date" should be written as "to date" when used in this context.

DATABASE SEARCHING

According to to PRISMA-P, the authors should present a full search strategy for at least one database. Please include the full search strategy for at least PubMed (in the main text or as an appendix), with exact terms, operators, and filters applied.

STUDY SELECTION

The authors should specify the software used for duplicate removal and for the screening process (e.g., EndNote, Rayyan, Covidence). Additionally, the protocol should describe the procedure to be followed if contacting study authors is necessary, for instance, to retrieve missing data or clarify eligibility.

INCLUSION AND EXCLUSION CRITERIA

1. The manuscript explicitly defines its objective in the abstract and introduction as assessing the methodological quality of systematic reviews and meta-analyses regarding "RA pharmacotherapy." However, the inclusion criteria permit non-pharmacological treatments, which contradicts the stated aim of the study and introduces significant clinical and methodological heterogeneity. Systematic reviews of lifestyle or alternative therapies often possess fundamentally different baseline methodological qualities, funding structures, and publication standards compared to those evaluating targeted synthetic or biological DMARDs. I recommend either revising the study objective to encompass all RA interventions or restricting the inclusion criteria strictly to pharmacotherapy to ensure the logical coherence of the analyzed data

2. The inclusion criteria related to outcomes are too vague and may lead to inconsistencies between reviewers during screening. The authors should provide at least a list of expected primary outcomes. Additionally, it should be clarified whether cost-effectiveness studies reporting clinical outcomes are also excluded.

3. Furthermore, the exclusion of NMA, IPD meta-analyses, and realist reviews based on AMSTAR 2 applicability is well justified (although it is a limitation and possibly undermines the study's primary objective to provide a comprehensive assessment of RA pharmacotherapy). However, the authors should clarify whether meta-analyses of observational studies are eligible for inclusion.

DATA EXTRACTION

Two minor issues should be addressed. First, the section inconsistently mixes past tense with future tense, suggesting the text may have been partially adapted from a published article. As this is a protocol, all verbs should be consistently written in the future tense. Second, the role of the third reviewer in resolving discrepancies is mentioned twice redundantly and should be consolidated into a single statement.

ROB ASSESSMENT

As previously outlined, all the verbs should be written in the future tense

DATA ANALYSIS

1. The protocol lists the geographic region of the corresponding author, categorized as a developing or developed country, as an independent variable for the regression analysis. However, the methodology does not specify the objective criteria or classification system that will be used to define these categories.

2. The proposed regression analysis includes preregistration as an independent variable to predict the overall AMSTAR 2 quality rating. However, protocol registration is explicitly evaluated as Item 2 in the AMSTAR 2 tool and is designated as a critical domain. Because the absence of preregistration deterministically downgrades the overall AMSTAR 2 confidence rating to "Low" or "Critically Low," including it as an independent predictor introduces severe circularity into the model. I recommend that the authors remove preregistration from the list of independent covariates, or, at least, test the model with or without this variable.

3. On the other hand, the proposed model omits the type of intervention as a covariate. Given that systematic reviews of newer biological agents are often subject to more stringent recent regulatory and reporting standards compared to older conventional therapies or lifestyle interventions, the intervention type can be a major potential confounder for methodological quality. I suggest adding intervention category as an independent variable in the primary regression model.

Reviewer #5: I found this publication well structured, clear and addresses a significant gap off literature regarding the quality of evidence in Rheumatoid Arthritis (RA) pharmacotherapy. The use of AMSTAR 2 is appropriate for this objective.

any how i had found a minured correction.

On page 10, line 184: "we will analysis association", should be corrected to "we will analyze the association."

Reviewer #6: I have carefully reviewed the manuscript. The authors propose and register a protocol for a meta-epidemiological study assessing the methodological quality of systematic reviews and meta-analyses conducted on rheumatoid arthritis treatments, particularly pharmacotherapy. The study aims to identify overall methodological trends in the literature and investigate associations between predefined covariates and study quality. Overall, the protocol is well designed, and the major methodological components have been appropriately addressed. The PRISMA-P checklist has been completed carefully, providing a strong basis for a rigorous and transparent study design. I appreciate the authors’ efforts and genuinely believe that this protocol is suitable for publication in its current form. However, I have a few minor suggestions:

Regarding the title, the authors may consider explicitly mentioning the concept of treatment, particularly pharmacotherapy, to better reflect the scope of the study and improve discoverability for readers. Some sentences in the Introduction are excessively long and may be difficult to follow (e.g., lines 49–53). In the Methods section, some statements appear repetitive, particularly in lines 97–99 and 109–110. The authors may consider revising these sections for conciseness.

Sincerely,

Reviewer #7: This manuscript presents a protocol for a meta-epidemiological study aiming to assess the methodological quality of systematic reviews and meta-analyses on rheumatoid arthritis (RA) pharmacotherapy using the AMSTAR 2 tool. The topic is timely and important given the rapid expansion of evidence synthesis studies in RA treatment. The protocol demonstrates several methodological strengths, including protocol registration, predefined covariates, and planned sensitivity analyses. However, improvements are needed regarding methodological clarity, conceptual consistency, grammar, reporting precision, and statistical explanation.

Reviewer #8: Respected authors, despite the fact that it is a good protocol, I fail to understand how is it a Meta-Epidemiological Study. It is only a review study assesses the strengths and weaknesses of the research methods applied in previous systematic reviews and meta analysis.

**********

what does this mean?). If published, this will include your full peer review and any attached files.

If you choose “no”, your identity will remain anonymous but your review may still be made public.

**Do you want your identity to be public for this peer review?**  For information about this choice, including consent withdrawal, please see our Privacy Policy

Reviewer #1: No

Reviewer #2: No

Reviewer #3: No

Reviewer #4: **Yes:**  Lara Campos

Reviewer #5: **Yes:**  sri chakradhar challagali

Reviewer #6: No

Reviewer #7: No

Reviewer #8: **Yes:**  Saira Akhlaq

**********

[NOTE: If reviewer comments were submitted as an attachment file, they will be attached to this email and accessible via the submission site. Please log into your account, locate the manuscript record, and check for the action link "View Attachments". If this link does not appear, there are no attachment files.]

To ensure your figures meet our technical requirements, please review our figure guidelines: https://journals.plos.org/plosone/s/figures

You may also use PLOS’s free figure tool, NAAS, to help you prepare publication quality figures: https://journals.plos.org/plosone/s/figures#loc-tools-for-figure-preparation.

NAAS will assess whether your figures meet our technical requirements by comparing each figure against our figure specifications.

Attachment Submitted filename: Peer Reviewer Report.pdf

# Author comment: Author response to Decision Letter 1

**DOI:** 10.1371/journal.pone.0359531.r002

26 Jul 2026

Reviewer #1

Original Comment (R1.1 - Search Strategy Appendix)

Search strategy Appendix: Although the authors refer to using “validated search filters” it would enhance the peer review process if they provided the complete search string for at least one database (e.g., PubMed) were included as a supplementary file now to verify the comprehensiveness of the MeSH terms.

Response: We fully agree with this suggestion. In the revised manuscript, we have added a new Supplementary Material 2 that provides the full search strategy for PubMed including exact MeSH/Emtree terms, free-text terms, Boolean operators, filters, and search dates. This ensures full compliance with the PRISMA-S checklist.

Original Comment (R1.2 — Screening Software)

Screening Software: Could the authors specify which software they used for screening and deduplication, such as Rayyan, Covidence, or Excel? This information would illustrate how blinding was achieved between the two independent reviewers.

Response: Thank you for this practical suggestion. We have now specified in Section 2.3 (Study Selection) that records will be imported into EndNote for de-duplication and into Rayyan for screening. We also explicitly state that the Rayyan blind-on mode will be enabled to ensure that reviewers are masked to each other's decisions throughout the screening process.

Original Comment (R1.3 — Inclusion of NMA)

Inclusion of NMA: The authors chose to exclude Network Meta-Analyses (NMA). While I understand the limitations of AMSTAR 2 in this regard, NMAs represent a significant portion of high-level evidence in RA. Therefore, a brief justification in the discussion regarding how this exclusion might affect the "comprehensiveness" of the quality map would be helpful.

Response: We appreciate this insightful comment. In the revised Limitations section (Discussion), we now provide a more substantive discussion of this issue. We explicitly acknowledge that excluding NMAs may limit the comprehensiveness of our evidence quality map, particularly for RA pharmacotherapy where NMAs are increasingly influential for comparative effectiveness assessments (e.g., head-to-head comparisons of JAK inhibitors vs. biologics). We also note that future methodological investigations should apply NMA-specific appraisal instruments (e.g., the ISPOR checklist or CINeMA) to address this gap.

Original Comment (R1.4 - Core Outcome Sets)

Core Outcome Sets: It may be advantageous to identify if the data extraction would classify outcomes using recognized core sets (e.g., OMERACT) in order to ensure consistency across various reviews.

Response: This is a very helpful suggestion. We have now incorporated the OMERACT RA core outcome set into our protocol. In Section 2.4 (Inclusion and Exclusion Criteria, Outcomes), we have predefined the following expected primary outcomes: disease activity (DAS28, ACR20/50/70 response), physical function (HAQ), pain (VAS or other validated scales), radiographic progression (Sharp/van der Heijde-modified Sharp scores), and serious adverse events, explicitly noting that these correspond to the OMERACT RA core outcome set. Additionally, Section 2.5 (Data Extraction) states that extracted outcomes will be categorized according to the OMERACT RA core outcome set to ensure harmonized classification across included reviews.

Reviewer #2

Original Comment (R2.1 - Inconsistent Scope)

1. Inconsistent scope

The title, abstract, and objective focus on RA pharmacotherapy (csDMARDs, bDMARDs, tsDMARDs), while the inclusion criteria admit complementary therapies and lifestyle interventions without restriction. This is an internal contradiction that propagates throughout the manuscript. The authors must make an explicit choice — restrict to pharmacological interventions with a clear operational definition, or expand to all therapeutic interventions — and apply it consistently to the title, objective, abstract, search strategy, and sensitivity analyses.

Response: We fully agree and thank the reviewer for identifying this critical inconsistency. We have made the following revisions throughout the manuscript:

1. Title has been revised from “Methodological Assessment of Systematic Review and Meta-analysis in Rheumatoid Arthritis: A Protocol for Meta-epidemiological Research” to “Methodological Quality of Systematic Reviews and Meta-analyses of Rheumatoid Arthritis Pharmacotherapy: A Protocol for a Meta-epidemiological Study Using AMSTAR 2”, which now explicitly focuses on pharmacotherapy.

2. Intervention criteria (Section 2.4) now restrict inclusion to systematic reviews/meta-analyses whose primary aim is to evaluate RA pharmacotherapy (csDMARDs, bDMARDs, and tsDMARDs). Reviews evaluating exclusively complementary/alternative therapies, lifestyle interventions, physiotherapy, or surgical procedures are explicitly excluded.

3. For reviews that assess both pharmacological and non-pharmacological interventions, we have added a decision rule: inclusion will be considered only if pharmacotherapy constitutes the primary comparison and the majority of included primary studies evaluate drug interventions; such decisions will be recorded and tested in a sensitivity analysis.

4. The title, abstract, objective, search strategy, and sensitivity analyses have all been revised to consistently reflect this pharmacotherapy-focused scope.

Original Comment (R2.2 - Mischaracterization of AMSTAR 2 Applicability)

1. Mischaracterization of AMSTAR 2 applicability

The exclusion of systematic reviews without quantitative synthesis, network meta-analyses, and IPD meta-analyses is justified by stating AMSTAR 2 is “not applicable.” This is partly incorrect: AMSTAR 2 can be applied to reviews without meta-analysis, with certain items treated as not applicable. The authors may legitimately restrict to pairwise quantitative syntheses as a scope decision, but must reframe the justification accordingly. The exclusion of network meta-analyses — the most policy-influential segment of RA pharmacotherapy evidence — demands explicit justification and a more substantive discussion of its implications.

Response: We sincerely thank the reviewer for this important correction. We have revised Section 2.4 (Study Design) to explicitly acknowledge that AMSTAR 2 can technically be applied to systematic reviews without meta-analysis (with certain items rated “not applicable”). We have reframed the exclusion of non-quantitative reviews as a deliberate scope decision rather than a claim of inapplicability. The revised text states:

“While AMSTAR 2 can technically be applied to systematic reviews without meta-analysis (with certain items rated 'not applicable'), we have elected as a deliberate scope decision to restrict inclusion to pairwise meta-analyses of RCTs or non-randomized intervention studies that have undergone quantitative synthesis. This restriction ensures comparability of AMSTAR 2 item ratings across reviews — particularly for Item 11 (appropriate meta-analytic methods) and Item 13 (accounting for risk of bias in interpretation) — and avoids heterogeneous 'not applicable' responses that would complicate our regression analysis. We explicitly acknowledge that this is a scope decision, not a claim that AMSTAR 2 is inapplicable to non-quantitative reviews.”

Regarding NMAs, we have also strengthened the discussion in the Limitations section acknowledging their importance and the implications of their exclusion, noting that “Future methodological investigations should apply NMA-specific appraisal instruments (e.g., the ISPOR checklist or CINeMA) to address this gap.”

Original Comment (R2.3 - Circularity in the Regression Model)

1. Circularity in the regression model

Pre-registration is both an independent variable in the regression model and a direct contributor to AMSTAR 2 Item 2, which, in turn, feeds into the overall rating used as the dependent variable. This is incorporation bias. The authors should either remove pre-registration as a predictor of the composite AMSTAR 2 rating, conduct a secondary analysis using an AMSTAR 2 score recalculated after excluding Item 2, or, at a minimum, acknowledge this as a named limitation.

Response: We greatly appreciate this methodologically astute observation. We have redesigned our analytical approach in Section 2.7 (Data Analysis) to address this circularity. Specifically:

• Pre-registration will not be entered as a predictor of the composite AMSTAR 2 rating in the primary model.

• Instead, we will conduct a pre-specified secondary analysis in which the overall AMSTAR 2 rating is recalculated after excluding Item 2 (registered protocol) from the algorithm; pre-registration will then be reintroduced as a predictor in this secondary model.

• The exclusion of Item 2 from the primary model is explicitly acknowledged as a limitation, and results from both models will be reported transparently.

This two-model approach allows us to assess the association between pre-registration and non-protocol components of methodological quality without incorporation bias.

Original Comment (R2.4 — Missing Inter-rater Reliability Plan and AMSTAR 2 Operationalization)

1. Missing inter-rater reliability plan and AMSTAR 2 operationalization

The protocol describes a dual-reviewer AMSTAR 2 assessment but provides no plan to quantify agreement (Cohen's kappa or ICC). This is the primary measurement of the study and cannot be omitted. Additionally, the handling of “partial yes” responses is unspecified, the seven critical AMSTAR 2 items are never named explicitly, and the overall rating terminology is inconsistent (“very low” is GRADE language; the correct AMSTAR 2 term is “critically low”). A dedicated paragraph should define the downgrading rules and list the seven critical items.

Response: We appreciate this thorough review of our methodological reporting. We have made the following additions in Section 2.6 (Methodological Quality Assessment Using AMSTAR 2):

1. Inter-rater reliability: We now specify that Cohen's kappa will be used for each item and a weighted kappa (or intraclass correlation coefficient, ICC) for the overall four-level rating, with interpretation thresholds (poor <0.40, moderate 0.40–0.60, good 0.61–0.80, very good >0.80).

2. “Partial Yes” handling: We now explicitly state that “Partial Yes” responses will be handled per Shea et al. (2017), counting as ‘Yes’ for the purpose of overall rating downgrading when the algorithm permits.

3. Seven critical items: We have explicitly listed all seven critical AMSTAR 2 items: Item 2 (registered protocol), Item 4 (adequacy of search), Item 7 (list of excluded studies with justification), Item 9 (risk-of-bias assessment of individual studies), Item 11 (appropriate meta-analytic methods), Item 13 (accounting for risk of bias in interpretation), and Item 15 (assessment of publication bias). A dedicated paragraph now describes the downgrading algorithm.

4. Terminology: We have corrected “Very Low” to “Critically Low” throughout the manuscript, consistent with AMSTAR 2 terminology.

Original Comment (R2.5 - No Search Strategy Provided)

1. No search strategy provided

The complete search string for at least one database is absent, violating PRISMA-P Item 17. Citing Escobar Liquitay et al. as a filter source is not a substitute. Additionally, given the unrestricted language policy and the authors' institutional context (China), the absence of Chinese databases (CNKI, Wanfang) warrants justification, particularly if complementary therapies remain in scope.

Response: We fully agree. The following revisions have been made:

1. Full search strategy: The complete search strategy for PubMed, including exact terms, Boolean operators, filters, and search dates, is now provided in Supplementary Material 2.

2. Chinese databases: We have expanded our search to include three Chinese databases: China National Knowledge Infrastructure (CNKI), Wanfang Data Knowledge Service Platform, and China Science and Technology Journal Database (VIP). Additionally, given the revised scope focusing on pharmacotherapy, the inclusion of Chinese databases ensures comprehensive coverage of the RA pharmacotherapy literature published in Chinese-language journals.

Original Comment (R2.6 - Regression Model Feasibility Not Addressed)

1. Regression model feasibility not addressed (both reviewers)

With nine covariates and a dependent variable expected to cluster heavily in the “critically low” category (72–90% in analogous published audits), the ordinal model risks instability and sparse cells. No sample-size estimate or power justification is provided. The authors must estimate the expected distribution of AMSTAR 2 ratings, pre-specify a rule for collapsing categories if cell counts are inadequate, and consider pre-specifying a hierarchy of predictors.

Response: We thank the reviewer for this important methodological concern. We have added a new section, “2.8 Model Feasibility and Sample Size,” which includes:

1. Expected distribution: We explicitly state, based on published methodological audits (Hammel et al. 2022; Karakasis et al. 2024), that we anticipate 70–90% of reviews will fall into the ‘Low’ or ‘Critically Low’ categories.

2. Events-per-variable rule: We apply the ≥10 reviews per predictor rule to the nine pre-specified covariates, targeting no fewer than 200 reviews for model stability.

3. Collapsing rule: We pre-specify that if any of the four rating categories contains <10 reviews, the dependent variable will be collapsed (e.g., ‘High/Moderate’ vs. ‘Low’ vs. ‘Critically Low’, or ‘Adequate’ [High/Moderate] vs. ‘Inadequate’ [Low/Critically Low]) and modeled with ordinal or binary logistic regression accordingly.

4. Predictor hierarchy: We pre-specify that the most theoretically important covariates (Cochrane source, intervention class, prospective registration, PRISMA adherence) will be entered in the primary model, with additional covariates added in a stepwise manner to assess model robustness.

Original Comment (R2.7 - Eligibility Criteria Framed at the Wrong Level)

1. Eligibility criteria framed at the wrong level (Reviewer 2)

The PICO eligibility criteria describe patients (1987/2010 RA classification criteria), but the unit of analysis is systematic reviews. Many otherwise eligible reviews will include primary studies that use variable or outdated diagnostic definitions. Criteria should be reframed at the review level: “eligible reviews must have included patients with rheumatoid arthritis as defined by the review authors.”

Response: This is an excellent point. We have revised the Participants criterion (Section 2.4) to be framed at the review level:

“Eligible reviews are those whose included population is patients with rheumatoid arthritis as defined by the review authors, irrespective of the specific classification criteria (e.g., 1987 ACR or 2010 ACR/EULAR) used by the primary studies. We frame this criterion at the review level: a review is eligible if its authors state that they included patients with RA, regardless of whether the underlying primary studies used the 1987 or 2010 criteria.”

Original Comment (R2 Additional Issues)

Additional Issues

• Section 2.6 title: “Risk of bias assessment” should be renamed “Methodological quality assessment using AMSTAR 2.” AMSTAR 2 is a critical appraisal tool, not a risk-of-bias instrument in the RoB 2/ROBINS-I sense.

• Forest plot: inappropriate for comparing item-level counts before/after AMSTAR 2 publication; replace with odds ratios or risk differences with confidence intervals. Replace the radar chart with stacked bar charts.

• Tense inconsistencies: several passages use past tense (“data were extracted,” “quality was assessed”), suggesting work may have already begun. If so, this must be disclosed transparently. The full methods section should be in the future tense for an unstarted protocol.

• Novelty claim: the assertion of being “the first” AMSTAR 2-based assessment of RA pharmacotherapy SRs is refuted by Shen et al. (2024, Front Pharmacol) and Ramalho et al. (2026, J Clin Med), neither of which is cited. The introduction requires revision.

• PROSPERO ID: CRD420261359521 appears to contain an extra digit; this should be verified.

• Minor: standardize “AMSTAR 2” spelling; correct “Cochrane origin”; resolve data availability inconsistency between the submission form and manuscript; align author contributions with the methods sections.

Response — Section 2.6 title: Agreed. We have renamed Section 2.6 to “Methodological Quality Assessment Using AMSTAR 2” and clarified that AMSTAR 2 is a critical-appraisal instrument, not a risk-of-bias tool in the RoB 2/ROBINS-I sense.

Response — Forest plot / radar chart: We have revised the analysis plan in Section 2.7 accordingly. Radar charts have been replaced with stacked bar charts for displaying the distribution of item ratings. Item-level adherence before vs. after the 2017 publication of AMSTAR 2 will be compared using odds ratios (or risk differences) with 95% CIs, rather than forest plots.

Response — Tense inconsistencies: We have carefully reviewed the entire manuscript and revised all methods sections to use the future tense, consistent with protocol reporting standards. The data extraction, quality assessment, and analysis sections now consistently use future-tense verbs (e.g., “will extract,” “will assess”).

Response — Novelty claim: We appreciate this important correction. We have revised the novelty claim throughout the manuscript. The Conclusion now states: “This study will provide a comprehensive, cross-drug-class AMSTAR 2-based methodological assessment of the RA pharmacotherapy evidence-synthesis field, complementing prior appraisals limited to specific complementary or single-drug interventions.” The Introduction has been similarly revised to acknowledge rather than dismiss prior work.

We have now added the following citations to the revised manuscript:

- Shen X, Liu X, Guo X, Hou X, Huang H, Feng Z. Systematic review of Janus kinases inhibitors for rheumatoid arthritis: methodology, reporting, and quality of evidence evaluation. Front Pharmacol. 2024;15:1459511. DOI: 10.3389/fphar.2024.1459511

- Ramalho B, Penedones A, Mendes D, Alves C. Quality of Systematic Reviews with Network Meta-Analyses on JAK Inhibitors in the Treatment of Rheumatoid Arthritis: Application of the AMSTAR 2 Scale. J Clin Med. 2026;15(2):725. DOI: 10.3390/jcm15020725

Response — PROSPERO ID:

Response: We have verified the PROSPERO registration and confirm that CRD420261359521 is correct. The registration is available at: https://www.crd.york.ac.uk/PROSPERO/view/CRD420261359521. We appreciate the reviewer's attention to detail.

Response — Minor items:

• “AMSTAR 2” spelling has been standardized throughout.

• “Cochrane origine” has been corrected to “Cochrane review or not” in the data extraction section.

• Data availability has been revised to: “No datasets were generated or analyzed during the current study. All relevant data from this study will be made available upon study completion and deposited in a public repository (Dryad).”

• Author contributions have been updated to reflect the revised team composition, including the addition of Delin Chen.

Reviewer #3

Original Comment (R3.1 - Rationale and Research Questions)

1. Rationale and Research Questions

The manuscript provides a valid rationale for conducting this study, and the need for a methodological assessment in this field is well justified. However, the research questions are not explicitly stated. While they can be inferred from the objectives, articulating them clearly would improve clarity and transparency.

Response: We have added a dedicated paragraph at the end of the Introduction explicitly stating three research questions:

(1) What is the overall AMSTAR 2 methodological quality of SRs/MAs of RA pharmacotherapy?

(2) Which study-level characteristics are associated with quality?

(3) Has quality changed after the 2017 publication of AMSTAR 2?

We also clarify that “as a meta-epidemiological study, the unit of analysis is the research study itself, examining associations between study-level characteristics and a study-level quality outcome rather than synthesizing clinical effects.”

Original Comment (R3.2 - Technical Soundness and Study Design)

1. Technical Soundness and Study Design

The protocol is generally well designed and technically sound. The planned analyses, including ordinal logistic regression, assessment of proportional odds, multicollinearity diagnostics, and sensitivity analyses, are appropriate for the stated objectives.

However, several methodological aspects require clarification to ensure full robustness:

• The unit of analysis is not defined. It is unclear how updated versions of the same review or duplicate publications will be handled.

• The protocol does not describe how the team will manage systematic reviews containing multiple meta‑analyses or complex analytic structures.

• The rationale for selecting specific covariates in the regression model should be more explicitly justified within a conceptual framework.

• These issues do not undermine the feasibility of the study but should be addressed to strengthen methodological rigor.

Response: We thank Reviewer #3 for these detailed methodological suggestions. All three points have been addressed:

(a) The unit of analysis is now explicitly defined in Section 2.6: “The unit of analysis is the individual systematic review/meta-analysis.”

(b) We have added handling rules in Section 2.6: “For duplicate or updated versions of the same review, the most recent and most complete version will be retained; for reviews containing multiple meta-analyses, AMSTAR 2 will be applied at the review level.”

(c) The rationale for covariate selection is now more explicitly justified within a conceptual framework in Section 2.7. We have added intervention class as a covariate specifically to account for differing regulatory and reporting standards across drug classes (csDMARDs, bDMARDs, tsDMARDs), which may influence the methodological rigor of reviews. The addition of PRISMA adherence as an exposure variable was also informed by the published conceptual framework of review quality determinants.

Original Comment (R3.3 - Replicability and Methodological Detail)

1. Replicability and Methodological Detail

Although the methodology is feasible, some elements are not described in sufficient detail to ensure full replicability:

• The protocol does not include a plan for assessing inter‑rater reliability (e.g., kappa or ICC) for AMSTAR 2 ratings, which is standard in methodological quality assessments.

• The handling of missing data is not fully described beyond contacting authors.

• The approach to reviews using non‑traditional meta‑analytic methods (e.g., Bayesian models, hierarchical models) is not specified.

• Providing additional detail in these areas would enhance reproducibility.

Response: All three points have been addressed:

(a) Inter-rater reliability: See response to R2.4. We now specify Cohen's kappa for each item and weighted kappa/ICC for the overall rating, with interpretation thresholds.

(b) Missing data: Section 2.5 now details a comprehensive missing data strategy: contact corresponding authors by email (up to two reminders over four weeks); if data remain unavailable, items will be scored conservatively with explicit documentation; a sensitivity analysis will be conducted excluding reviews with >20% missing data to assess robustness.

(c) Non-traditional meta-analytic methods: Section 2.7 now states: “Reviews using Bayesian or hierarchical pairwise meta-analytic models remain eligible. For such reviews, AMSTAR 2 items will be applied as applicable: Item 11 (appropriate meta-analytic methods) will be evaluated based on whether the Bayesian/hierarchical model was appropriately specified and justified; Item 13 (accounting for risk of bias) will be assessed using the same criteria as for frequentist meta-analyses. We will not exclude these reviews solely on the basis of their statistical approach.”

Original Comment (R3.4 - Data Availability)

1. Data Availability

The manuscript states that all relevant data will be made available upon study completion. This is acceptable for a protocol, although the authors may consider specifying the intended repository or platform to improve transparency.

Response: We have revised the Data Availability section to specify: “All relevant data from this study will be made available upon study completion and deposited in a public repository (Dryad).”

Reviewer #4

Original Comment (R4.1 - English Language Revision)

INTRODUCTION

The introduction is well-structured and provides a clear rationale for the study's objectives. However, the manuscript would benefit from a thorough English language revision to improve grammatical accuracy and clarity. There are several instances of subject-verb disagreement and missing relative pronouns that detract from the professional quality of the work.

Response: We have carefully revised the language throughout the manuscript. Specific grammatical issues identified by the reviewer have been corrected (see below), and a full language review has been performed to improve consistency and professional quality.

Original Comment (R4.2 - Line 36)

Line 36

The phrase “A meta-analysis included 67 studies showed...” is grammatically incomplete. It should be revised to “A meta-analysis including 67 studies showed...” or “A meta-analysis that included 67 studies showed...”.

Response: Corrected to “A meta-analysis that included 67 studies showed...” (Introduction, first paragraph).

Original Comment (R4.3 — Line 45)

Line 45

The subject “Therapeutic agents” is plural, but the verb used is “has”. This should be corrected to “have delivered”.

Response: Corrected to “have delivered” (Introduction, second paragraph).

Original Comment (R4.4 - Line 38)

Line 38

The text mentions the “GDB study”; please confirm if this refers to the GBD (Global Burden of Disease) study, as this is a common typographical error.

Response: Thank you for catching this typographical error. Corrected to “GBD (Global Burden of Disease) study” (Introduction, first paragraph).

Original Comment (R4.5 - Line 54)

Line 54

“precision presentation” would be more idiomatic as “precise presentation.”

Response: Corrected to “precise presentation” (Introduction). The revised text now reads: “Systematic reviews must provide a precise presentation of current knowledge.”

Original Comment (R4.6 - Line 66)

Line 66

“To-date” should be written as “to date” when used in this context.

Response: The sentence containing this instance has been restructured in the revision. The revised Introduction now reads: “no prior study has provided a comprehensive, cross-drug-class methodological assessment” - removing the “to-date” phrasing entirely.

Original Comment (R4.7 - Database Searching)

DATABASE SEARCHING

According to PRISMA-P, the authors should present a full search strategy for at least one database. Please include the full search strategy for at least PubMed (in the main text or as an appendix), with exact terms, operators, and filters applied.

Response: Addressed - see response to R1.1 and R2.5. PubMed strategy was provided in Supplementary Material 2 as an example.

Original Comment (R4.8 - Study Selection)

STUDY SELECTION

The authors should specify the software used for duplicate removal and for the screening process (e.g., EndNote, Rayyan, Covidence). Additionally, the protocol should describe the procedure to be followed if contacting study authors is necessary, for instance, to retrieve missing data or clarify eligibility.

Response: Both points addressed. Screening software (EndNote for deduplication, Rayyan with blind-on mode for screening) is specified in Section 2.3. The contacting-authors procedure is described in Section 2.5: up to two email reminders over four weeks, with a conservative scoring rule for unreported data and a sensitivity analysis excluding reviews with >20% missing data.

Original Comment (R4.9 - Inclusion/Exclusion Criteria: Non-pharmacological Treatments)

INCLUSION AND EXCLUSION CRITERIA

1. The manuscript explicitly defines its objective in the abstract and introduction as assessing the methodological quality of systematic reviews and meta-analyses regarding “RA pharmacotherapy.” However, the inclusion criteria permit non-pharmacological treatments, which contradicts the stated aim of the study and introduces significant clinical and methodological heterogeneity. Systematic reviews of lifestyle or alternative therapies often possess fundamentally different baseline methodological qualities, funding structures, and publication standards compared to those evaluating targeted synthetic or biological DMARDs. I recommend either revising the study objective to encompass all RA interventions or restricting the inclusion criteria strictly to pharmacotherapy to ensure the logical coherence of the analyzed data.

Response: Addressed - see response to R2.1. We have restricted inclusion criteria strictly to pharmacotherapy (csDMARDs, bDMARDs, tsDMARDs) and revised title, abstract, objective, and all subsequent sections to consistently reflect this scope.

Original Comment (R4.10 - Inclusion/Exclusion Criteria: Outcomes Vagueness)

1. The inclusion criteria related to outcomes are too vague and may lead to inconsistencies between reviewers during screening. The authors should provide at least a list of expected primary outcomes. Additionally, it should be clarified whether cost-effectiveness studies reporting clinical outcomes are also excluded.

Response: We have added a list of expected primary outcomes based on the OMERACT RA core outcome set (see R1.4): disease activity (DAS28, ACR20/50/70), physical function (HAQ), pain (VAS), radiographic progression (Sharp/vdH-modified Sharp scores), and serious adverse events. We have also clarified that health-economic studies reporting only economic outcomes without clinical effectiveness data are excluded, unless clinical-effectiveness data are embedded within the same review.

Original Comment (R4.11 - Inclusion/Exclusion Criteria: Observational Studies)

1. Furthermore, the exclusion of NMA, IPD meta-analyses, and realist reviews based on AMSTAR 2 applicability is well justified (although it is a limitation and possibly undermines the study's primary objective to provide a comprehensive assessment of RA pharmacotherapy). However, the authors should clarify whether meta-analyses of observational studies are eligible for inclusion.

Response: We have explicitly stated in Section 2.4 (Study Design) that meta-analyses synthesizing observational intervention studies (e.g., cohort studies, case-control studies) are eligible, provided they have conducted quantitative synthesis. This decision will be recorded and tested in a sensitivity analysis by comparing reviews based on RCTs only vs. those including observational studies.

Original Comment (R4.12 - Data Extraction: Tense and Redundancy)

DATA EXTRACTION

Two minor issues should be addressed. First, the section inconsistently mixes past tense with future tense, suggesting the text may have been partially adapted from a published article. As this is a protocol, all verbs should be consistently written in the future tense. Second, the role of the third reviewer in resolving discrepancies is mentioned twice redundantly and should be consolidated into a single statement.

Response: All verbs in the data extraction section now consistently use future tense. The redundant mention of the third reviewer has been consolidated into a single statement.

Original Comment (R4.13 - ROB Assessment: Future Tense)

ROB ASSESSMENT

As previously outlined, all verbs should be written in the future tense.

Response: Addressed. Section 2.6 now consistently uses future tense throughout.

Original Comment (R4.14 - Data Analysis: Geographic Region Criteria)

DATA ANALYSIS

1. The protocol lists the geographic region of the corresponding author, categorized as a developing or developed country, as an independent variable for the regression analysis. However, the methodology does not specify the objective criteria or classification system that will be used to define these categories.

Response: We have specified in Section 2.7 that this will be classified using the World Bank income classification (high-income vs. non-high-income economies), based on the country's classification in the year of publication.

Original Comment (R4.15 - Data Analysis: Circularity of Preregistration)

1. The proposed regression analysis includes preregistration as an independent variable to predict the overall AMSTAR 2 quality rating. However, protocol registration is explicitly evaluated as Item 2 in the AMSTAR 2 tool and is designated as a critical domain. Because the absence of preregistration deterministically downgrades the overall AMSTAR 2 confidence rating to “Low” or “Critically Low,” including it as an independent predictor introduces severe circularity into the model. I recommend that the authors remove preregistration from the list of independent covariates, or, at least, test the model with or without this variable.

Response: Addressed - see response to R2.3. We have removed pre-registration from the primary model and added a secondary analysis recalculating AMSTAR 2 after excluding Item 2.

Original Comment (R4.16 - Data Analysis: Missing Intervention Type Covariate)

1. On the other hand, the proposed model omits the type of intervention as a covariate. Given that systematic reviews of newer biological agents are often subject to more stringent recent regulatory and reporting standards compared to older conventional therapies or lifestyle interventions, the intervention type can be a major potential confounder for methodological quality. I suggest adding intervention category as an independent variable in the primary regression model.

Response: We fully agree and have added intervention class (csDMARDs only / bDMARDs only / tsDMARDs only / mixed / other pharmacotherapy) as a covariate in the regression model. The rationale is stated explicitly in Section 2.7: this accounts for differing regulatory and reporting standards across drug classes, which may influence the methodological rigor of reviews.

Reviewer #5

Original Comment (R5.1 - Line 184)

I found this publication well structured, clear and addresses a significant gap of literature regarding the quality of evidence in Rheumatoid Arthritis (RA) pharmacotherapy. The use of AMSTAR 2 is appropriate for this objective.

Anyhow I had found a minor correction.

On page 10, line 184: “we will analysis association”, should be corrected to “we will analyze the association.”

Response: We thank Reviewer #5 for the positive assessment and for noting this error. Corrected. Section 2.7 now reads: “we will analyze the associations between each independent variable and the methodological-quality rating.”

Reviewer #6

Original Comment (R6.1 - Title)

I have carefully reviewed the manuscript. The authors propose and register a protocol for a meta-epidemiological study assessing the methodological quality of systematic reviews and meta-analyses conducted on rheumatoid arthritis treatments, particularly pharmacotherapy. The study aims to identify overall methodological trends in the literature and investigate associations between predefined covariates and study quality. Overall, the protocol is well designed, and the major methodological components have been appropriately addressed. The PRISMA-P checklist has been completed carefully, providing a strong basis for a rigorous and transparent study design. I appreciate the authors' efforts and genuinely believe that this protocol is suitable for publication in its current form. However, I have a few minor suggestions:

Regarding the title, the authors may consider explicitly mentioning the concept of treatment, particularly pharmacotherapy, to better reflect the scope of the study and improve discoverability for readers.

Response: We sincerely thank Reviewer #6 for the positive evaluation. We agree with the suggestion regarding the title. The revised title now reads: “Methodological Quality of Systematic Reviews and Meta-analyses of Rheumatoid Arthritis Pharmacotherapy: A Protocol for a Meta-epidemiological Study Using AMSTAR 2”, which explicitly mentions pharmacotherapy and improves both scope reflection and discoverability.

Original Comment (R6.2 - Long Sentences in Introduction)

Some sentences in the Introduction are excessively long and may be difficult to follow (e.g., lines 49-53).

Response: We have carefully revised the Introduction to improve readability. Long sentences have been broken into shorter, clearer segments. For example, the compound sentence describing the roles of systematic reviews has been restructured for clarity.

Original Comment (R6.3 - Repetitive Statements in Methods)

In the Methods section, some statements appear repetitive, particularly in lines 97–99 and 109–110. The authors may consider revising these sections for conciseness.

Response: We have reviewed the Methods section and consolidated redundant statements, particularly in the data extraction section where the third reviewer's role in resolving discrepancies was mentioned twice.

Reviewer #7

Full Review

Title: Methodological Assessment of Systematic Review and Meta-analysis in Rheumatoid Arthritis: A Protocol for Meta-epidemiological Research

Recommendation: Minor Revision

Overall Evaluation

This manuscript presents a protocol for a meta-epidemiological study aiming to assess the methodological quality of systematic reviews and meta-analyses on rheumatoid arthritis (RA) pharmacotherapy using the AMSTAR 2 tool. The topic is timely and important given the rapid expansion of evidence synthesis studies in RA treatment. The protocol demonstrates several methodological strengths, including protocol registration, predefined covariates, and planned sensitivity analyses. However, improvements are needed regarding methodological clarity, conceptual consistency, grammar, reporting precision, and statistical explanation.

Major Strengths

• Timely and relevant topic addressing methodological quality in RA evidence synthesis

• Protocol registered in PROSPERO

• Use of AMSTAR 2 as an established methodological appraisal tool

• Planned multivariable and sensitivity analyses

• Independent screening and data extraction procedures clearly described

Major Weaknesses

• Several grammatical and language inconsistencies throughout the manuscript

• Methodological details require greater precision and clarity

• Insufficient justification for some exclusion criteria

• Conceptual overlap between methodological quality and risk of bias terminology

• Limited explanation of planned regression assumptions and sample size considerations

• Data availability statement may not fully align with PLOS protocol standards

Section-by-Section Comments

• Abstract: Clear structure but contains grammatical inconsistencies and overly broad claims.

• Introduction: Strong rationale but somewhat repetitive and occasionally overstated.

• Methods: Generally rigorous but requires clearer justification for exclusions and statistical procedures.

• Database Searching: Well described, though exact search strings should be provided in supplementary materials.

• Study Selection: Appropriate dual-reviewer approach.

• Risk of Bias Assessment: AMSTAR 2 procedures are appropriate but terminology should distinguish methodological quality from risk of bias.

• Data Analysis: Comprehensive approach, but regression assumptions and model stability require additional clarification.

• Discussion: Reasonable overview of study importance, though somewhat repetitive.

• Conclusion: Appropriate but should avoid overstating future impact.

Examples of Line-by-Line Shortcomings

• Line 20: ‘associations between covariate and the methodological quality’ should read ‘associations between covariates and methodological quality.’

• Line 85: Missing space before ‘(PRISMA-P Group et al. 2015)’.

• Line 92: Section heading 'database searching' should be capitalized consistently.

• Line 149: ‘detailed reason for judgement’ should read ‘detailed reasons for judgment.’

• Line 184: ‘we will analysis association’ should read ‘we will analyze associations.'’

• Line 193: ‘R studio’ should be standardized as ‘RStudio.’

• Line 242: Data availability wording may not fully comply with PLOS protocol transparency requirements.

Recommendations for Authors

• Conduct thorough professional English-language editing

• Clarify regression assumptions and model justification

• Provide stronger rationale for exclusion criteria

• Standardize terminology related to methodological quality and risk of bias

• Improve consistency of formatting, capitalization, and reporting style

• Expand details on planned handling of missing data and model diagnostics

• Ensure data availability statement aligns with PLOS protocol requirements

Minor Revision.

This protocol addresses an important methodological gap in rheumatoid arthritis evidence synthesis and demonstrates several strengths in design and planning. However, revisions are required to improve methodological clarity, statistical explanation, language quality, and reporting consistency before publication.

Response: We sincerely thank Reviewer #7 for this thorough, structured, and constructive review. Every point raised has been addressed in the revised manuscript. Below we map each weakness, line-by-line issue, and recommendation to specific revisions:

Major Weaknesses — Addressed

Weakness How Addressed

Several grammatical and language inconsistencies Full language revision conducted; see responses to R4.1–R4.6 and line-by-line fixes below

Methodological details require greater precision and clarity Unit of analysis defined (Section 2.6); duplicate/updated review handling added (Section 2.6); inter-rater reliability added (Section 2.6); missing data strategy added (Section 2.5); Bayesian/hierarchical model handling added (Section 2.7)

Insufficient justification for some exclusion criteria Exclusion criteria reframed as scope decisions with detailed rationale (Section 2.4); NMA/IPD-MA exclusion discussed in Limitations

Conceptual overlap between methodological quality and risk of bias Section 2.6 renamed to “Methodological Quality Assessment Using AMSTAR 2”; explicit distinction added between critical appraisal and risk-of-bias tools

Limited explanation of regression assumptions and sample size New Section 2.8 (Model Feasibility and Sample Size) added; proportional odds assumption testing specified

Data availability statement alignment with PLOS standards Updated to specify Dryad repository

Line-by-Line Shortcomings — Addressed

Line Original Issue Revised Wording

Line 20: “associations between covariate and the methodological quality” Corrected to “associations between covariates and methodological quality” (Abstract)

Line 85: Missing space before “(PRISMA-P Group et al. 2015)” Space added: “Protocols (PRISMA-P Group et al. 2015)”

Line 92: “database searching” Capitalized to “Database Searching” (Section 2.2)

Line 149: “detailed reason for judgement” Revised to “We will provide detailed justification for each item rating in supplementary materials” (Section 2.6)

Line 184: “we will analysis association” Corrected to “we will analyze the associations” (Section 2.7)

Line 193: “R studio” Standardized to “R (Version 4.3.1) with the RStudio integrated development environment (Version 2023.06.2)” (Section 2.7)

Line 242: Data availability wording Revised to specify Dryad repository

Section-by-Section Comments — Addressed

Section Issue Revision

Abstract Grammatical inconsistencies and overly broad claims Language corrected; claim softened from “first” to “comprehensive, cross-drug-class...complementing prior appraisals”

Introduction Repetitive and occasionally overstated Streamlined; novelty claims revised

Methods Requires clearer justification Exclusion criteria reframed; statistical procedures elaborated

Database Searching Search strings needed Full strategies added as Supplementary Material 2

Study Selection Appropriate — no change needed Enhanced with EndNote/Rayyan details

Risk of Bias Assessment Terminology overlap Section renamed; AMSTAR 2 vs. RoB distinction clarified

Data Analysis Assumptions need clarification New Section 2.8; Brant test specified

Discussion Somewhat repetitive Streamlined for conciseness

Conclusion Overstated future impact Revised to measured language

Reviewer #8

Original Comment

Respected authors, despite the fact that it is a good protocol, I fail to understand how it is a Meta-Epidemiological Study. It is only a review study assessing the strengths and weaknesses of the research methods applied in previous systematic reviews and meta-analyses.

Response: We appreciate this comment, which gives us an opportunity to clarify our study design. A meta-epidemiological study is defined as research that uses systematic review methods to investigate the design, conduct, analysis, or reporting of a collection of related research studies (Murad and Wang 2017). The defining characteristic is that the unit of analysis is the research study itself rather than the individual patient or participant.

In our protocol:

1. The unit of analysis is each systematic review/meta-analysis, not individual patients.

2. We examine associations between study-level characteristics (e.g., prospective registration, funding source, Cochrane origin, intervention class) and a study-level quality outcome (AMSTAR 2 rating).

3. We are not synthesizing clinical treatment effects but rather investigating methodological patterns across a body of evidence.

We recognize that this definition may not be universally familiar. In the revised manuscript, we have added a clarifying sentence at the end of the Introduction that explicitly states: “Crucially, this is a meta-epidemiological investigation: the unit of analysis is the systematic review itself, and the outcome of interest is methodological quality, rather than the synthesis of pooled clinical effect estimates from primary trials (Murad and Wang 2017).” We hope this clarifies the nature of our study.

Manuscript Change Checklist

# Revision Location

1 Title revised to focus on RA pharmacotherapy Title page

2 Author list updated (Delin Chen added) Author list

3 Three explicit research questions added Introduction (new paragraph)

4 Meta-epidemiological study definition clarified Introduction

5 Inclusion criteria restricted to pharmacotherapy Section 2.4 Intervention

6 Participants reframed at review level Section 2.4 Participants

7 Expected outcomes predefined (OMERACT core set) Section 2.4 Outcomes

8 Observational-study MA eligibility clarified Section 2.4 Study design

9 NMA/IPD-MA exclusion reframed as scope decision Section 2.4 + Discussion

10 Chinese databases added (CNKI, Wanfang, VIP) Section 2.2

11 Full search strategies in Supplementary Material 2 Section 2.2

12 EndNote + Rayyan blind-on screening specified Section 2.3

13 Missing data strategy with sensitivity analysis Section 2.5

14 Section 2.6 renamed (methodological quality, not RoB) Section 2.6

15 Inter-rater reliability (Cohen's kappa, ICC) added Section 2.6

16 Seven critical AMSTAR 2 items explicitly listed Section 2.6

17 “Very Low” → “Critically Low” throughout Section 2.6 +full text

18 “Partial Yes” handling specified Section 2.6

19 Duplicate/updated review handling rules Section 2.6

20 Regression model redesigned (no incorporation bias) Section 2.7

21 Intervention class added as covariate Section 2.7

22 World Bank classification for geographic region Section 2.7

23 Bayesian/hierarchical model handling specified Section 2.7

24 Radar chart → stacked bar; forest plot → OR/RD Section 2.7

25 New Section 2.8: Model Feasibility and Sample Size Section 2.8 (new)

26 Novelty claim softened throughout Abstract, Introduction, Discussion

27 Language revision (grammar, tense, terminology) Full text

28 Data availability statement updated (Dryad) Data Availability

29 Author contributions updated Author Contributions

30 All line-by-line corrections (see R7 table) Multiple locations

Attachment Submitted filename: response to reviewers.docx

# Peer review: Decision Letter 1

**DOI:** 10.1371/journal.pone.0359531.r003

**Authors:** Athanasios G. Pantelis

10 Aug 2026

Dear Dr. hu,

Thank you for submitting your manuscript to PLOS ONE. After careful consideration, we feel that it has merit but does not fully meet PLOS ONE’s publication criteria as it currently stands. Therefore, we invite you to submit a revised version of the manuscript that addresses the points raised during the review process.

==============================

**ACADEMIC EDITOR:**  Four reviewers have recommended **Minor Revision**

Please submit your revised manuscript by Sep 24 2026 11:59PM. If you will need more time than this to complete your revisions, please reply to this message or contact the journal office at plosone@plos.org. When you're ready to submit your revision, log on to https://www.editorialmanager.com/pone/ and select the 'Submissions Needing Revision' folder to locate your manuscript file.

- A letter that responds to each point raised by the academic editor and reviewer(s). You should upload this letter as a separate file labeled 'Response to Reviewers'.
- A marked-up copy of your manuscript that highlights changes made to the original version. You should upload this as a separate file labeled 'Revised Manuscript with Track Changes'.
- An unmarked version of your revised paper without tracked changes. You should upload this as a separate file labeled 'Manuscript'.

If you would like to make changes to your financial disclosure, please include your updated statement in your cover letter. Guidelines for resubmitting your figure files are available below the reviewer comments at the end of this letter.

If applicable, we recommend that you deposit your laboratory protocols in protocols.io to enhance the reproducibility of your results. Protocols.io assigns your protocol its own identifier (DOI) so that it can be cited independently in the future. For instructions see: https://journals.plos.org/plosone/s/submission-guidelines#loc-laboratory-protocols. Additionally, PLOS ONE offers an option for publishing peer-reviewed Lab Protocol articles, which describe protocols hosted on protocols.io. Read more information on sharing protocols at https://plos.org/protocols?utm_medium=editorial-email&utm_source=authorletters&utm_campaign=protocols.

As the corresponding author, your ORCID iD is verified in the submission system and will appear in the published article. PLOS supports the use of ORCID, and we encourage all coauthors to register for an ORCID iD and use it as well. Please encourage your coauthors to verify their ORCID iD within the submission system before final acceptance, as unverified ORCID iDs will not appear in the published article. *Only*  the individual author can complete the verification step; PLOS staff *cannot*  verify ORCID iDs on behalf of authors.

We look forward to receiving your revised manuscript.

Kind regards,

Athanasios G. Pantelis

Academic Editor

PLOS One

Journal Requirements:

1. If the reviewer comments include a recommendation to cite specific previously published works, please review and evaluate these publications to determine whether they are relevant and should be cited. There is no requirement to cite these works unless the editor has indicated otherwise.

2. Please review your reference list to ensure that it is complete and correct. If you have cited papers that have been retracted, please include the rationale for doing so in the manuscript text, or remove these references and replace them with relevant current references. Any changes to the reference list should be mentioned in the rebuttal letter that accompanies your revised manuscript. If you need to cite a retracted article, indicate the article’s retracted status in the References list and also include a citation and full reference for the retraction notice.

[Note: HTML markup is below. Please do not edit.]

Reviewers' comments:

Reviewer's Responses to Questions

**Comments to the Author**

1. Does the manuscript provide a valid rationale for the proposed study, with clearly identified and justified research questions?

Reviewer #1: Yes

Reviewer #2: Yes

Reviewer #5: Yes

Reviewer #8: Yes

**********

2. Is the protocol technically sound and planned in a manner that will lead to a meaningful outcome and allow testing the stated hypotheses?

Reviewer #1: Yes

Reviewer #2: Yes

Reviewer #5: Yes

Reviewer #8: Yes

**********

3. Is the methodology feasible and described in sufficient detail to allow the work to be replicable?

Reviewer #1: Yes

Reviewer #2: Yes

Reviewer #5: Yes

Reviewer #8: Yes

**********

4. Have the authors described where all data underlying the findings will be made available when the study is complete?

The PLOS Data policy

Reviewer #1: Yes

Reviewer #2: Yes

Reviewer #5: Yes

Reviewer #8: Yes

**********

5. Is the manuscript presented in an intelligible fashion and written in standard English?

Reviewer #1: Yes

Reviewer #2: Yes

Reviewer #5: Yes

Reviewer #8: Yes

**********

Please use the space provided to explain your answers to the questions above and, if applicable, provide comments about issues authors must address before this protocol can be accepted for publication. You may also include additional comments for the author, including concerns about research or publication ethics.

You may also provide optional suggestions and comments to authors that they might find helpful in planning their study.

Reviewer #1: Recommendation: Accept (Re-review Satisfied / Minor Formatting Correction Required)

Comments to the Academic Editor:

I commend the authors for their thorough, rigorous, and highly responsive revision.

They have meticulously addressed all reviewer concerns, successfully transitioning the manuscript into a methodologically sound and transparent meta-epidemiological protocol.

Below is my detailed assessment of the revised manuscript across all major sections:

1. Scope, Title, and Introduction

Scope Reframing: The title and overall framing have been properly narrowed to focus strictly on RA pharmacotherapy (csDMARDs, bDMARDs, and tsDMARDs), ensuring conceptual clarity.

Conceptual Precision: The introduction now clearly defines the meta-epidemiological framework (specifying the systematic review as the unit of analysis).

Research Questions & Typos: The authors explicitly articulated the three core research questions and resolved minor errors (e.g., correcting "GDB" to "GBD").

Novelty Claim: The unwarranted precedence claims (e.g., "the first study") were appropriately removed and replaced with a nuanced, well-supported rationale highlighting their cross-category pharmacotherapy evaluation.

2. Abstract Assessment (Minor Production Correction Required)

While the scientific content of the abstract was successfully updated, a formatting/copy-paste oversight occurred during revision that requires correction prior to publication:

Copy-Paste Error: The revised Methods text was accidentally pasted directly into the Background section (lines 13–20). This resulted in severe text duplication, the complete disappearance of the Objective section, and a redundant repetition of "the Cochrane Library" (lines 13–14).

Database Inconsistency: The newly added Chinese databases (CNKI, Wanfang, VIP) appear in the duplicated Background section but were omitted from the actual Methods paragraph (lines 21–26).

Action Required: The editorial team/production should ask the authors to clean up the Abstract structure (restoring the explicit Objective statement and removing the duplicated text block).

3. Methodology & Statistical Rigor

The authors have executed all requested statistical and methodological enhancements impeccably:

Data Extraction & Missing Data (Section 2.5): Protocol protocols for managing missing data (email reminders and conservative scoring) and a pre-specified sensitivity analysis excluding studies with >20% missing data have been seamlessly integrated.

AMSTAR 2 Application (Section 2.6): Properly retitled to "Methodological Quality" rather than "Risk of Bias".

The authors clearly defined the unit of analysis, handling of updated reviews, inter-rater agreement metrics (Cohen’s kappa/ICC with standard threshold interpretations), handling of "Partial Yes" items, and explicitly listed all 7 critical domains. (Minor note: The term "Critically Low" should be verified for consistent usage in Section 2.6, replacing one lingering instance of "Very Low").

Mitigation of Incorporation Bias (Section 2.7): The potential circularity regarding pre-registration was brilliantly resolved. The authors wisely excluded pre-registration from the primary AMSTAR 2 model and constructed a secondary model evaluating pre-registration after omitting Item 2 from the score calculation.

Model Feasibility & Power (Section 2.8): The newly added Section 2.8 fully satisfies sample size adequacy concerns by setting a minimum target of N >= 200 reviews, enforcing Events-Per-Variable (EPV >= 10) guidelines, and pre-specifying category collapsing rules to ensure regression stability.

4. Discussion, Limitations, and Data Availability

Methodological Limitations: The authors thoughtfully addressed the exclusion of Network Meta-Analyses (NMAs) and Individual Patient Data (IPD) meta-analyses. They explicitly acknowledged this as a scope limitation (due to AMSTAR 2 structural constraints) and highlighted its implications for comparative effectiveness research (e.g., JAK inhibitors vs. biologics), while recommending specific tools (CINeMA/ISPOR) for future evaluators.

Data Policy: The data availability statement is fully compliant with PLOS standards, designating the Dryad repository for open-data deposition upon project completion.

Overall Conclusion

This manuscript has undergone a remarkable transformation and now represents a robust, transparent, and high-quality protocol. I recommend Acceptance, contingent only on the authors resolving the minor text duplication/formatting error in the Abstract.

Reviewer #2: The authors have addressed all major first-round concerns satisfactorily. The scope is now restricted to RA pharmacotherapy, the AMSTAR 2 section has been correctly renamed and operationalized, inter-rater reliability is planned, the incorporation bias issue is resolved in principle through a two-model approach, and six databases with a full search strategy are now specified. The manuscript is close to acceptable. The following issues require correction before publication:

The abstract contains a structural duplication: a free-standing Methods paragraph listing six databases appears before the Background heading, while the formal Methods field lists only three. One unified, accurate paragraph should replace both.

The regression plan contains a new internal contradiction introduced in revision: Section 2.7 correctly excludes pre-registration from the primary model to avoid incorporation bias, but Section 2.8 then lists "prospective registration" among the primary model's theoretically important covariates. Pre-registration must be removed from Section 2.8's primary predictor hierarchy and retained only in the secondary model.

The scope of eligible pharmacotherapy is still underspecified. Eligibility criteria restrict to csDMARDs/bDMARDs/tsDMARDs, yet the regression model includes an "other pharmacotherapy" category and sensitivity analysis 3 implies additional drug classes may be eligible. The authors must explicitly state whether glucocorticoids, NSAIDs, biosimilars, and intra-articular pharmacological agents are in or out.

The description of "Partial Yes" handling remains imprecise. The current wording — "counting as Yes for the purpose of overall rating downgrading" — is the inverse of the algorithm's logic: it is a "No" on a critical item, not a "Yes," that triggers downgrading. The sentence should state that a "Partial Yes" on a critical item does not constitute a critical weakness and therefore does not trigger downgrading, consistent with the AMSTAR 2 algorithm. Additionally, the downgrading rules should be stated explicitly: no or one non-critical weakness = High; more than one non-critical weakness = Moderate; one critical flaw = Low; more than one critical flaw = Critically Low.

Sensitivity analysis 1 still reads "high/medium vs. low/very low" — this should be "High/Moderate vs. Low/Critically Low."

Introduction line 94 uses past tense ("we conducted a comprehensive methodological appraisal"), which is inappropriate for an unstarted protocol and raises a transparency concern about whether data collection has begun.

Two citation errors appear in the Introduction: Shea et al. 2017 is misattributed as a prior quality appraisal of complementary therapies (it is the AMSTAR 2 tool paper), and Rodriguez-García et al. 2021 is described as a JAK inhibitor study (it concerns intra-articular therapies). Both require correction. Additionally, three reference pairs are duplicated in the reference list (Karakasis 2024a/b, Rodriguez-García 2021a/b, Shea 2017a/b — identical DOIs) and should be merged.

Minor items: "MeSH/Emtree" in reference to the PubMed strategy should read "MeSH terms" (Emtree is Embase-specific); "to-date" with a hyphen reappears in the Discussion; "comprehensive… standardized comprehensive" contains a word repetition; the data availability field in the submission form does not mention the Dryad repository, inconsistently with the manuscript text.

Reviewer #5: You state that for reviews containing multiple meta-analyses, "AMSTAR 2 will be applied at the review level." If a single systematic review contains multiple meta-analyses—some of which adhere to high reporting standards and some of which do not—how will you derive a single, valid AMSTAR 2 rating for the review?

You anticipate that 70–90% of reviews will fall into the ‘Low’ or ‘Critically Low’ categories, yet you intend to run a regression model with nine covariates. Even with 200 reviews, if your data are heavily skewed (e.g., 180 reviews in the ‘Low/Critically Low’ categories and only 20 in the ‘High/Moderate’ categories), your regression model will likely lack the power to detect significant associations and will be prone to overfitting.

Reviewer #8: Respected Authors,

You have made enormous improvements by incorporating the reviewers recommendations. Kindly, also adjust the references as per ICMJE guidelines.

**********

what does this mean?). If published, this will include your full peer review and any attached files.

If you choose “no”, your identity will remain anonymous but your review may still be made public.

**Do you want your identity to be public for this peer review?**  For information about this choice, including consent withdrawal, please see our Privacy Policy

Reviewer #1: No

Reviewer #2: No

Reviewer #5: **Yes:**  sri chakradhar challagali

Reviewer #8: **Yes:**  Saira Akhlaq

**********

[NOTE: If reviewer comments were submitted as an attachment file, they will be attached to this email and accessible via the submission site. Please log into your account, locate the manuscript record, and check for the action link "View Attachments". If this link does not appear, there are no attachment files.]

To ensure your figures meet our technical requirements, please review our figure guidelines: https://journals.plos.org/plosone/s/figures

You may also use PLOS’s free figure tool, NAAS, to help you prepare publication quality figures: https://journals.plos.org/plosone/s/figures#loc-tools-for-figure-preparation.

NAAS will assess whether your figures meet our technical requirements by comparing each figure against our figure specifications.

# Author comment: Author response to Decision Letter 2

**DOI:** 10.1371/journal.pone.0359531.r004

9 Sep 2026

Journal Requirements

Requirement 1: If the reviewer comments include a recommendation to cite specific previously published works, please review and evaluate these publications to determine whether they are relevant and should be cited. There is no requirement to cite these works unless the editor has indicated otherwise.

Response: None of the reviewers recommended the citation of specific previously published works. The four references added in this revision (references 18-21) were added on our own initiative to support newly introduced methodological content - Firth penalized regression (18), Bayesian ordinal regression via brms (19), the pmsampsize sample-size approach (20), and the events-per-parameter rule (21) - and are cited at the relevant methods sections.

Requirement 2: Please review your reference list to ensure that it is complete and correct. … Any changes to the reference list should be mentioned in the rebuttal letter that accompanies your revised manuscript.

Response: We have carefully reviewed and corrected the reference list. The changes are as follows:

1. Three pairs of duplicate entries with identical DOIs have been merged, leaving a single entry for each: Shea et al. 2017, Rodríguez-García et al. 2021, and Karakasis et al. 2024.

2. Four references have been added to support the newly introduced statistical methods: Firth 1993 (reference 18, bias-reduced penalized logistic regression); Bürkner 2017 (reference 19, brms R package for Bayesian ordinal regression); Riley et al. 2020 (reference 20, sample size for prediction models, pmsampsize); Peduzzi et al. 1996 (reference 21, events-per-parameter simulation study).

3. The entire list has been reformatted according to the ICMJE Recommendations, with references numbered in order of first citation in the text (see also our response to Reviewer #8).

4. The revised list contains 22 references, all of which are cited in the text and none of which is orphaned.

5. No retracted publications are cited.

Reviewer #1

Recommendation: Accept (Re-review Satisfied / Minor Formatting Correction Required).

Response: We sincerely thank Reviewer #1 for the thorough and positive assessment of the first revision, and for identifying the Abstract formatting error. Both action items raised have been corrected, as detailed below.

Reviewer #1, Comment 1 (Abstract)

Copy-Paste Error: The revised Methods text was accidentally pasted directly into the Background section (lines 13–20). This resulted in severe text duplication, the complete disappearance of the Objective section, and a redundant repetition of “the Cochrane Library” (lines 13–14). Database Inconsistency: The newly added Chinese databases (CNKI, Wanfang, VIP) appear in the duplicated Background section but were omitted from the actual Methods paragraph (lines 21–26). Action Required: The editorial team/production should ask the authors to clean up the Abstract structure (restoring the explicit Objective statement and removing the duplicated text block).

Response: We apologize for this error, which occurred during text replacement in the first revision. The Abstract has been completely restructured: the duplicated block has been removed, the explicit Objective statement restored, and the six databases are now listed consistently in the single Methods paragraph. The revised Abstract now reads:

Changes in the manuscript (Abstract, Objective and Methods):

Objective: This protocol describes a meta-epidemiological study that will systematically appraise the methodological quality of published systematic reviews and meta-analyses of RA pharmacotherapy (csDMARDs, bDMARDs, and tsDMARDs) using the AMSTAR 2 instrument and explore study-level factors associated with methodological quality.

Methods: We will systematically search PubMed, Embase, the Cochrane Library and three Chinese databases-China National Knowledge Infrastructure (CNKI), Wanfang Data and China Science and Technology Journal Database-from inception to June 1, 2026. Two independent reviewers will screen studies, extract data and assess methodological quality using the AMSTAR 2 instrument; an overall rating (high, moderate, low, critically low) will be derived for each review. Binary logistic regression comparing “Adequate” (High/Moderate) versus “Inadequate” (Low/Critically Low) reviews will be used for the primary analysis, with ordinal logistic regression fitted as a pre-specified exploratory analysis; pre-specified sensitivity and robustness analyses (including penalized and Bayesian estimation for sparse categories) will test the stability of findings.

Reviewer #1, Comment 2 (Minor note on “Critically Low”)

Minor note: The term “Critically Low” should be verified for consistent usage in Section 2.6, replacing one lingering instance of “Very Low”.

Response: We have verified the entire manuscript. All four-level ratings now read “High, Moderate, Low, or Critically Low,” and the term “Very Low” no longer appears anywhere in the manuscript, including Section 2.6:

Changes in the manuscript (Section 2.6):

Each item will be judged as “Yes”, “Partial Yes”, or “No”; an overall rating of High, Moderate, Low, or Critically Low will be derived using the AMSTAR 2 algorithm.

Reviewer #2

The authors have addressed all major first-round concerns satisfactorily. … The manuscript is close to acceptable. The following issues require correction before publication:

Response: We thank Reviewer #2 for the positive overall assessment and for the precise identification of the remaining issues. All eight points have been addressed as follows.

Reviewer #2, Comment 1 (Abstract structural duplication)

The abstract contains a structural duplication: a free-standing Methods paragraph listing six databases appears before the Background heading, while the formal Methods field lists only three. One unified, accurate paragraph should replace both.

Response: As detailed in our response to Reviewer #1 (Comment 1), the Abstract has been completely restructured into a single unified set of fields: the duplicated pre-Background block was removed, the explicit Objective statement was restored, and the Methods field now lists all six databases (PubMed, Embase, the Cochrane Library, CNKI, Wanfang Data, and the China Science and Technology Journal Database). Please see the full revised Abstract text quoted in our response to Reviewer #1, Comment 1.

Reviewer #2, Comment 2 (Internal contradiction on pre-registration in Section 2.8)

Section 2.7 correctly excludes pre-registration from the primary model to avoid incorporation bias, but Section 2.8 then lists “prospective registration” among the primary model’s theoretically important covariates. Pre-registration must be removed from Section 2.8’s primary predictor hierarchy and retained only in the secondary model.

Response: We thank the reviewer for catching this contradiction introduced during the first revision. The predictor hierarchy in Section 2.8 has been rewritten: the primary model now contains only three covariates (Cochrane source, intervention class, PRISMA adherence), the remaining six covariates are examined only in secondary models, and prospective registration is explicitly retained only in the secondary model after exclusion of Item 2:

Changes in the manuscript (Section 2.8):

The primary model will contain three theoretically important covariates-Cochrane systematic review source (binary), intervention class (categorical, four levels), and PRISMA adherence (continuous). … The remaining six pre-specified exposures and covariates will be examined only in secondary models, and no stepwise variable selection will be performed, to avoid overfitting and multiple-testing inflation. Prospective registration will not be entered as a predictor in the primary model (see Section 2.7) and will be retained only in the secondary model after exclusion of Item 2.

Reviewer #2, Comment 3 (Scope of eligible pharmacotherapy)

Eligibility criteria restrict to csDMARDs/bDMARDs/tsDMARDs, yet the regression model includes an “other pharmacotherapy” category and sensitivity analysis 3 implies additional drug classes may be eligible. The authors must explicitly state whether glucocorticoids, NSAIDs, biosimilars, and intra-articular pharmacological agents are in or out.

Response: We have made the scope fully explicit in Section 2.4. Biosimilars are classified under the corresponding bDMARD category and remain eligible; reviews whose primary aim is exclusively glucocorticoids, NSAIDs, or intra-articular pharmacological agents are excluded; and the “other pharmacotherapy” category has been deleted from the model, which is now four-level. Boundary decisions for mixed pharmacological/non-pharmacological reviews are recorded and tested in sensitivity analysis 5:

Changes in the manuscript (Section 2.4, Intervention):

For reviews that assess both pharmacological and non-pharmacological interventions, inclusion will be considered only if pharmacotherapy constitutes the primary comparison and the majority of included primary studies evaluate drug interventions; such decisions will be recorded and tested in a sensitivity analysis (Section 2.7, analysis 5). … For scope clarity, biosimilars of biological DMARDs will be classified under the corresponding bDMARD category and remain eligible; reviews whose primary aim is exclusively the evaluation of glucocorticoids, non-steroidal anti-inflammatory drugs, or intra-articular pharmacological agents in RA will be excluded, as these lie outside the csDMARDs/bDMARDs/tsDMARDs framework central to this study.

Changes in the manuscript (Section 2.7):

The model also includes intervention class (csDMARDs only / bDMARDs only / tsDMARDs only / mixed, where "mixed" denotes reviews evaluating more than one DMARD class) as a covariate, added to account for differing regulatory and reporting standards across drug classes, which may influence the methodological rigor of reviews.

Reviewer #2, Comment 4 (“Partial Yes” logic and explicit downgrading rules)

The current wording - “counting as Yes for the purpose of overall rating downgrading” - is the inverse of the algorithm’s logic: it is a “No” on a critical item, not a “Yes,” that triggers downgrading. The sentence should state that a "Partial Yes" on a critical item does not constitute a critical weakness and therefore does not trigger downgrading, consistent with the AMSTAR 2 algorithm. Additionally, the downgrading rules should be stated explicitly: no or one non-critical weakness = High; more than one non-critical weakness = Moderate; one critical flaw = Low; more than one critical flaw = Critically Low.

Response: We agree, and the sentence has been rewritten to match the AMSTAR 2 algorithm, with the four downgrading rules now stated explicitly:

Changes in the manuscript (Section 2.6):

In accordance with the AMSTAR 2 algorithm (6), a “Partial Yes” on a critical item does not constitute a critical weakness and therefore does not by itself trigger downgrading; only “No” responses are counted as weaknesses. The overall rating is derived as follows: High-no or only one non-critical weakness; Moderate-more than one non-critical weakness; Low-one critical weakness with or without non-critical weaknesses; Critically Low-more than one critical weakness with or without non-critical weaknesses.

Reviewer #2, Comment 5 (Sensitivity analysis 1 naming)

Sensitivity analysis 1 still reads “high/medium vs. low/very low” - this should be “High/Moderate vs. Low/Critically Low.”

Response: The sensitivity-analysis section has been rewritten in full, and the old list (including the “high/medium vs. low/very low” wording) has been removed. Because the primary analysis itself is now the binary comparison of “Adequate” (High/Moderate) versus “Inadequate” (Low/Critically Low) reviews, that former dichotomization has been promoted from a sensitivity analysis to the primary analysis. Sensitivity analysis 1 now tests an alternative three-category collapse of the four-level outcome:

Changes in the manuscript (Section 2.7):

Our primary analysis will model methodological quality as a binary outcome-“Adequate” (overall rating High or Moderate) versus “Inadequate” (Low or Critically Low)-using binary logistic regression. … To test the robustness of the results, seven pre-specified sensitivity analyses will be conducted: (1) the four-level outcome will be collapsed alternatively into three categories (High/Moderate vs. Low vs. Critically Low) and re-analyzed where cell counts permit; …

Reviewer #2, Comment 6 (Past tense in the Introduction)

Introduction line 94 uses past tense (“we conducted a comprehensive methodological appraisal”), which is inappropriate for an unstarted protocol and raises a transparency concern about whether data collection has begun.

Response: We agree. The sentence has been corrected to the future tense, and the tense of the entire manuscript has been checked for consistency with the protocol status (no data collection has begun; all study activities are expressed with “will”):

Changes in the manuscript (Introduction, final paragraph):

To bridge this critical evidence gap, we will conduct a comprehensive methodological appraisal of published SRs/MAs on RA pharmacotherapy using the AMSTAR 2 tool.

Reviewer #2, Comment 7 (Citation misattributions and duplicate references)

Shea et al. 2017 is misattributed as a prior quality appraisal of complementary therapies (it is the AMSTAR 2 tool paper), and Rodriguez-García et al. 2021 is described as a JAK inhibitor study (it concerns intra-articular therapies). Both require correction. Additionally, three reference pairs are duplicated in the reference list (Karakasis 2024a/b, Rodriguez-García 2021a/b, Shea 2017a/b — identical DOIs) and should be merged.

Response: We apologize for these attribution errors, which have been corrected. Shea et al. 2017 is now cited only as the AMSTAR 2 tool paper, and the three prior appraisal domains are now attributed to the correct references: complementary/alternative therapies to Li et al. (7), single-agent JAK inhibitors to Shen et al. (8), and intra-articular therapies to Rodríguez-García et al. (9, the overview of intra-articular therapies). The three duplicate pairs have been merged into single entries (see also Journal Requirement 2 above).

Changes in the manuscript (Introduction):

Accordingly, methodological appraisal tools (AMSTAR 2) have been developed to ensure transparent, standardized, and rigorous conduct of systematic reviews (6). [Reference 6 is Shea et al. 2017, the AMSTAR 2 tool paper.]

To our knowledge, while previous AMSTAR 2-based appraisals have focused on specific complementary/alternative therapies (7), single-agent JAK inhibitors (8), or intra-articular therapies (9), no prior study has provided a cross-drug-class methodological assessment covering the full spectrum of csDMARDs, bDMARDs, and tsDMARDs with regression-based factor analysis.

Reviewer #2, Comment 8 (Minor items)

Minor items: “MeSH/Emtree” in reference to the PubMed strategy should read “MeSH terms” (Emtree is Embase-specific); “to-date” with a hyphen reappears in the Discussion; “comprehensive… standardized comprehensive” contains a word repetition; the data availability field in the submission form does not mention the Dryad repository, inconsistently with the manuscript text.

Response: All four items have been corrected:

1. “MeSH/Emtree” now reads “MeSH terms” in both places where the search strategy is described; “Emtree” has been deleted from the manuscript.

2. The hyphenated “to-date” has been corrected in the Discussion.

3. The word repetition “standardized comprehensive” has been removed.

4. The Data Availability field in the submission form has been updated to mention the Dryad repository, consistent with the manuscript text.

Changes in the manuscript (Section 2.2):

We will use a combined strategy of MeSH terms and free text words and literature related to systematic reviews will be searched using specifically developed and validated search filters without restrictions on publication date or language (12). … The full search strategy for PubMed, including exact MeSH terms, free-text terms, Boolean operators, filters, and search dates, is provided in Supplementary Material 2, in accordance with PRISMA-S.

Changes in the manuscript (Discussion):

To date, no study has explored potential factors influencing methodological quality.

Changes in the manuscript (Discussion):

… conduct a comprehensive, large-scale, standardized assessment of the methodological quality of systematic reviews and meta-analyses related to drug treatments for RA …

Changes in the manuscript (Data Availability statement):

No datasets were generated or analyzed during the current study. All relevant data from this study will be made available upon study completion and deposited in a public repository (Dryad).

Reviewer #5

Reviewer #5, Comment 1 (Single rating for reviews containing multiple meta-analyses)

You state that for reviews containing multiple meta-analyses, “AMSTAR 2 will be applied at the review level.” If a single systematic review contains multiple meta-analyses-some of which adhere to high reporting standards and some of which do not-how will you derive a single, valid AMSTAR 2 rating for the review?

Response: We thank the reviewer for this important question. Section 2.6 now pre-specifies an explicit, deterministic rule that removes operator discretion: review-level items are rated for the review as a whole, whereas meta-analysis-level items are rated according to the meta-analysis of the lowest quality (i.e., the most conservative rating across the included meta-analyses). The number of meta-analyses within each review and any within-review inconsistency are recorded and reported, and sensitivity analysis 4 excludes reviews containing multiple meta-analyses to test the robustness of this rule:

Changes in the manuscript (Section 2.6):

To derive a single valid rating for a review containing multiple meta-analyses, review-level items (e.g., Items 2, 4, 7, 9) will be rated for the review as a whole, whereas meta-analysis-level items (e.g., Items 11, 13, 14, 15) will be rated conservatively: when the included meta-analyses differ, the item will be rated according to the meta-analysis of the lowest quality, to avoid operator discretion and to ensure comparability across reviews. The number of meta-analyses within each review and any within-review inconsistency will be recorded in the extraction form and reported in supplementary tables.

Reviewer #5, Comment 2 (Power and overfitting under a skewed outcome)

You anticipate that 70-90% of reviews will fall into the “Low” or “Critically Low” categories, yet you intend to run a regression model with nine covariates. Even with 200 reviews, if your data are heavily skewed (e.g., 180 reviews in the “Low/Critically Low” categories and only 20 in the “High/Moderate” categories), your regression model will likely lack the power to detect significant associations and will be prone to overfitting.

Response: We agree with this assessment, and the analysis plan has been redesigned as a coherent chain of pre-specified safeguards, each addressing a specific link in the concern the reviewer raises:

1. Outcome dichotomized to maximize stability under the expected skew. The primary analysis models “Adequate” (High/Moderate) versus “Inadequate” (Low/Critically Low) as a binary outcome; the four-level ordinal model has been demoted to a pre-specified exploratory analysis that is fitted only when every collapsed category contains at least 10 reviews.

2. Parsimonious primary model: three covariates, five parameters. Only Cochrane source, intervention class (four levels), and PRISMA adherence enter the primary model; counting parameters rather than variables, the four-level intervention class contributes three indicators, so k = 5. The remaining six pre-specified covariates are examined only in secondary models, and no stepwise selection is performed.

3. Formal sample-size planning. As a meta-epidemiological study, this investigation is a census of the available evidence base: the realized sample size will be determined by the number of eligible reviews identified. Based on a conservative PubMed search (title/abstract) that retrieves more than 2400 records, we anticipate at least 300 eligible reviews. We will therefore verify that this anticipated scale satisfies the statistical requirements of the primary model by formally estimating the minimum sample size required for stable regression using the pmsampsize package (20), with the Peduzzi rule (N = 10k/p) as a cross-check; if the realized sample or event count falls short, the pre-specified analysis ladder (Section 2.8) applies. The formal pmsampsize calculation will be performed prior to data analysis and reported with the final manuscript.

4. Estimation matched to the anticipated events-per-parameter ratio. With a 10-15% expected proportion of Adequate reviews, N ≥ 300 yields 30-45 events in the smaller group, i.e., 30–45 ÷ 5 = 6–9 events per parameter, below the conventional threshold of 10. Firth’s bias-reduced penalized logistic regression with penalized profile-likelihood confidence intervals is therefore the pre-specified default estimator; unpenalized maximum likelihood will additionally be reported only if the realized ratio reaches 10.

5. Direct answer to the reviewer’s scenario (e.g., 20 Adequate vs. 180 Inadequate reviews). In such a realized dataset the events-per-parameter ratio would be 20 ÷ 5 = 4, and the pre-specified plan responds automatically: Firth estimation is applied, the analysis ladder is triggered, and — if the realized Adequate proportion falls below 10% - all regression results are interpreted as exploratory, with the descriptive appraisal remaining the principal contribution.

6. Bayesian robustness check for sparse ordinal categories. A Bayesian ordinal regression with weakly informative priors (brms) is pre-specified to provide shrinkage against overfitting when the exploratory ordinal model is fitted.

7. A three-tier analysis ladder that adapts the model to the realized sample size, and a priori power calculations reported with the final model.

Changes in the manuscript (Section 2.8):

We therefore set N ≥ 300 included reviews as the target, and we will estimate the required minimum sample size formally using the pmsampsize package (20), with the Peduzzi rule (N = 10k/p, where k is the number of predictors and p the proportion in the smaller outcome group) as a cross-check (21).

The primary model will contain three theoretically important covariates-Cochrane systematic review source (binary), intervention class (categorical, four levels), and PRISMA adherence (continuous). Counting model parameters rather than variables, the four-level intervention class contributes three indicator variables, so the model contains five parameters in total; with N ≥ 300 and a 10-15% expected proportion of Adequate reviews (30-45 events in the smaller outcome group), the events-per-parameter ratio is 30-45 ÷ 5 = 6-9, below the conventional threshold of 10 (21). The primary analysis will therefore be estimated using Firth's bias-reduced penalized logistic regression with penalized profile-likelihood confidence intervals, as pre-specified in Section 2.7; unpenalized maximum-likelihood estimates will additionally be reported if the realized events-per-parameter ratio reaches 10.

Changes in the manuscript (Section 2.8, analysis ladder):

To avoid dependence on any single realized sample size, we pre-specify the following analysis ladder. If 200–299 reviews are included, the three-predictor primary model is retained, with the events-per-parameter ratio recomputed for the observed distribution (Firth estimation applying as above). If 100–199 reviews are included, the primary model is reduced to the two most theoretically important covariates (Cochrane source and PRISMA adherence), and the remaining covariates are analyzed univariately. If fewer than 100 reviews are included, regression is abandoned in favor of the descriptive methodological-quality appraisal. … The realized sample size and the analysis tier adopted will be reported with the PRISMA flow diagram, and a priori power calculations based on the anticipated event rate in the least frequent rating category will be reported to demonstrate whether the final model is adequately powered, with the number of predictors adjusted to the available sample. If the realized proportion of Adequate reviews is very low (e.g., <10%), the regression results will be interpreted as exploratory, as acknowledged in the Limitations section.

## Reviewer #8

Reviewer #8, Comment 1 (References per ICMJE guidelines)

You have made enormous improvements by incorporating the reviewers recommendations. Kindly, also adjust the references as per ICMJE guidelines.

Response: We thank the reviewer for the encouraging assessment and this suggestion. The reference list has been reformatted according to the ICMJE Recommendations: references are numbered and cited in order of first appearance in the text; when there are more than six authors, the first six are listed followed by “et al.”; journal titles are abbreviated in NLM style; and the year, volume(issue), page range, and DOI are provided for each entry. For example:

Examples of the reformatted entries (References 1 and 6):

1. Gravallese EM, Firestein GS. Rheumatoid Arthritis - Common Origins, Divergent Mechanisms. N Engl J Med. 2023 Feb 9;388(6):529–42. doi:10.1056/NEJMra2103726

2. Shea BJ, Reeves BC, Wells G, Thuku M, Hamel C, Moran J, et al. AMSTAR 2: a critical appraisal tool for systematic reviews that include randomised or non-randomised studies of healthcare interventions, or both. BMJ. 2017 Sep 21;358:j4008. doi:10.1136/bmj.j4008

The complete revised list (22 references) appears in the References section of the revised manuscript; all reference-list changes are declared in our response to Journal Requirement 2 above.

Attachment Submitted filename: Response_to_Reviewers_auresp_2.docx

# Editor report: Decision Letter 2

**DOI:** 10.1371/journal.pone.0359531.r005

**Authors:** Athanasios G. Pantelis

15 Sep 2026

Methodological Quality of Systematic Reviews and Meta-analyses of Rheumatoid Arthritis Pharmacotherapy: A Protocol for a Meta-epidemiological Study Using AMSTAR 2

PONE-D-26-18105R2

Dear Dr. Hu,

We’re pleased to inform you that your manuscript has been judged scientifically suitable for publication and will be formally accepted for publication once it meets all outstanding technical requirements.

Within one week, you’ll receive an e-mail detailing the required amendments. When these have been addressed, you’ll receive a formal acceptance letter and your manuscript will be scheduled for publication.

An invoice will be generated when your article is formally accepted. Please note, if your institution has a publishing partnership with PLOS and your article meets the relevant criteria, all or part of your publication costs will be covered. Please make sure your user information is up-to-date by logging into Editorial Manager at Editorial Manager® and clicking the ‘Update My Information' link at the top of the page. For questions related to billing, please contact billing support.

If your institution or institutions have a press office, please notify them about your upcoming paper to help maximize its impact. If they’ll be preparing press materials, please inform our press team as soon as possible -- no later than 48 hours after receiving the formal acceptance. Your manuscript will remain under strict press embargo until 2 pm Eastern Time on the date of publication. For more information, please contact onepress@plos.org.

Kind regards,

Athanasios G. Pantelis

Academic Editor

PLOS One

Additional Editor Comments (optional):

Reviewers' comments:

# Editor report: Acceptance letter

**DOI:** 10.1371/journal.pone.0359531.r006

**Authors:** Athanasios G. Pantelis

PONE-D-26-18105R2

PLOS One

Dear Dr. hu,

I'm pleased to inform you that your manuscript has been deemed suitable for publication in PLOS One. Congratulations! Your manuscript is now being handed over to our production team.

At this stage, our production department will prepare your paper for publication. This includes ensuring the following:

* All references, tables, and figures are properly cited

* All relevant supporting information is included in the manuscript submission,

* There are no issues that prevent the paper from being properly typeset

You will receive further instructions from the production team, including instructions on how to review your proof when it is ready. Please keep in mind that we are working through a large volume of accepted articles, so please give us a few days to review your paper and let you know the next and final steps.

Lastly, if your institution or institutions have a press office, please let them know about your upcoming paper now to help maximize its impact. If they'll be preparing press materials, please inform our press team within the next 48 hours. Your manuscript will remain under strict press embargo until 2 pm Eastern Time on the date of publication. For more information, please contact onepress@plos.org.

You will receive an invoice from PLOS for your publication fee after your manuscript has reached the completed accept phase. If you receive an email requesting payment before acceptance or for any other service, this may be a phishing scheme. Learn how to identify phishing emails and protect your accounts at https://explore.plos.org/phishing.

If we can help with anything else, please email us at customercare@plos.org.

Thank you for submitting your work to PLOS One and supporting open access.

Kind regards,

PLOS One Editorial Office Staff

on behalf of

Dr. Athanasios G. Pantelis

Academic Editor

PLOS One
